# Plexin-B2 orchestrates collective stem cell dynamics via actomyosin contractility, cytoskeletal tension and adhesion

**DOI:** 10.1038/s41467-021-26296-7

**Published:** 2021-10-14

**Authors:** Chrystian Junqueira Alves, Rafael Dariolli, Jonathan Haydak, Sangjo Kang, Theodore Hannah, Robert J. Wiener, Stefanie DeFronzo, Rut Tejero, Gabriele L. Gusella, Aarthi Ramakrishnan, Rodrigo Alves Dias, Alexandre Wojcinski, Santosh Kesari, Li Shen, Eric A. Sobie, José Paulo Rodrigues Furtado de Mendonça, Evren U. Azeloglu, Hongyan Zou, Roland H. Friedel

**Affiliations:** 1 Nash Family Department of Neuroscience, Friedman Brain Institute, Icahn School of Medicine at Mount Sinai, New York, NY USA; 2 Department of Pharmacological Sciences, Icahn School of Medicine at Mount Sinai, New York, NY USA; 3 Division of Nephrology, Department of Medicine, Icahn School of Medicine at Mount Sinai, New York, NY USA; 4 Division of Cardiology, Department of Medicine, Icahn School of Medicine at Mount Sinai, New York, NY USA; 5 Department of Physics, Federal University of Juiz de Fora, Juiz de Fora, Brazil; 6 Pacific Neuroscience Institute and John Wayne Cancer Institute at Providence Saint John’s Health Center, Santa Monica, CA USA; 7 Department of Neurosurgery, Icahn School of Medicine at Mount Sinai, New York, NY USA

**Keywords:** Mechanotransduction, Actin, Neural stem cells, Embryonic stem cells

## Abstract

During morphogenesis, molecular mechanisms that orchestrate biomechanical dynamics across cells remain unclear. Here, we show a role of guidance receptor Plexin-B2 in organizing actomyosin network and adhesion complexes during multicellular development of human embryonic stem cells and neuroprogenitor cells. Plexin-B2 manipulations affect actomyosin contractility, leading to changes in cell stiffness and cytoskeletal tension, as well as cell-cell and cell-matrix adhesion. We have delineated the functional domains of Plexin-B2, RAP1/2 effectors, and the signaling association with ERK1/2, calcium activation, and YAP mechanosensor, thus providing a mechanistic link between Plexin-B2-mediated cytoskeletal tension and stem cell physiology. Plexin-B2-deficient stem cells exhibit premature lineage commitment, and a balanced level of Plexin-B2 activity is critical for maintaining cytoarchitectural integrity of the developing neuroepithelium, as modeled in cerebral organoids. Our studies thus establish a significant function of Plexin-B2 in orchestrating cytoskeletal tension and cell-cell/cell-matrix adhesion, therefore solidifying the importance of collective cell mechanics in governing stem cell physiology and tissue morphogenesis.

## Abstract (web summary)

Biomechanical mechanisms orchestrating stem cell dynamics in development remain unclear. Here the authors show that guidance receptor Plexin-B2 organizes actomyosin contractility, cytoskeletal tension and adhesion during multicellular development of human embryonic stem cells and neuroprogenitor cells.

## Introduction

The multicellular organization relies on biomechanical processes that mediate the generation, transmission, and sensing of mechanical force in cells^1–3^. For instance, during tissue morphogenesis, individual cells constantly rearrange against one another, which requires rapid cytoskeletal adjustment and establishment of a collective actomyosin network and adhesion complexes across cells in order to maintain cell morphology and tissue cohesion^4,5^. Cell-generated forces are transmitted through cell–cell junctions and focal adhesion sites at the interface between the cytoskeleton and extracellular matrix (ECM), which in turn provide important signals to inform cell fate decisions^6^. While a number of mechanotransduction pathways that enable cells to perceive and adapt to physical environment have been mapped^1,7^, the molecular mechanisms orchestrating collective cytoskeletal dynamics are less clear.

One informative example of multicellular dynamics is the self-assembly of cultured human embryonic stem cells (hESCs) into an epithelium-like colony, with stereotypical apical-basolateral polarization, E-cadherin-based adherens junctions, ZO-1-containing tight junctions, and integrin-based focal adhesions (FAs) at the interface between cytoskeleton and ECM^8,9^. During hESC colony formation, a network of cortical actomyosin is formed across cells in the colony interior, while continuous filamentous actin (F-actin) band emerges spanning multiple cells at the colony periphery. This leads to in-plane traction forces that are primarily localized at colony edges, as revealed by traction force microscopy^6^. Another example of complex mechanomorphogenesis is the process of neural tube closure and ventricle formation during neurodevelopment^10^. How hESCs and neuroprogenitor cells (NPCs) orchestrate cytoskeletal tension across cells and organize adhesion complexes at cell–cell and cell–matrix junctions to maintain cytoarchitectural integrity is incompletely understood.

Plexin-B2 is an axon guidance receptor widely expressed in the developing brain, in particular at the ventricular surface, supporting its role in regulating neuroprogenitors^11^. Plexin-B2 plays an essential role in neural tube closure and cerebellar granule cell migration during neurodevelopment^12–14^. Mouse mutant studies also revealed a role of Plexin-B2 in coordinating the proliferation and migration of neuroblasts in the rostral migratory stream^12,13,15,16^. Plexin-B2 is also expressed in brain tumor cells^17^ and epidermal stem cells^18^. Recent studies have begun to unravel the essential roles of Plexins in mediating cellular interactions in a variety of tissue contexts, including vascular development, immune activation, bone homeostasis, and epithelial morphogenesis and repair^18–28^. However, the underlying mechanisms remain unclear.

Interestingly, phylogenetic analyses unveiled an early evolutionary emergence of Plexins that predates the appearance of nervous systems^29,30^. Plexins have a highly conserved domain structure throughout all metazoan clades, particularly the ring-shaped extracellular domain and the intracellular Ras-GAP (GTPase activating protein) domain^31,32^. The Ras-GAP domain of Plexins can inactivate Ras or Rap small GTPases^33^, which have a pleiotropic network of effectors that control cytoskeletal dynamics and cell–cell adhesion^34,35^. Hence, we posit that Plexins may have evolved during the transition from unicellular to multicellular organisms to adjust cytoskeletal tension and adhesion forces at cell–cell and cell–matrix junctions^29^.

Here, we show that Plexin-B2 orchestrates multicellular dynamics of hESCs and hNPCs by controlling collective cytoskeletal dynamics and tensional forces. This, in turn, affects the maturation of adhesive complexes at cell–cell and cell–matrix junctions, thereby impacting membrane-association of β-catenin, focal adhesion, and integrin activation, leading to alteration of cell morphology and tissue geometry, as well as stem cell behaviors. We further delineate Plexin-B2 signaling domains, the role of RAP1/2 effectors, and the signaling relationship with the mechanosensitive transcription factor YAP (Yes-associated protein)^36,37^, thus providing a mechanistic link between Plexin-B2 signaling and stem cell physiology in a multicellular organization.

## Results

### Plexin-B2 affects the expansion and geometry of the hESC colony

**Fig. 1:**
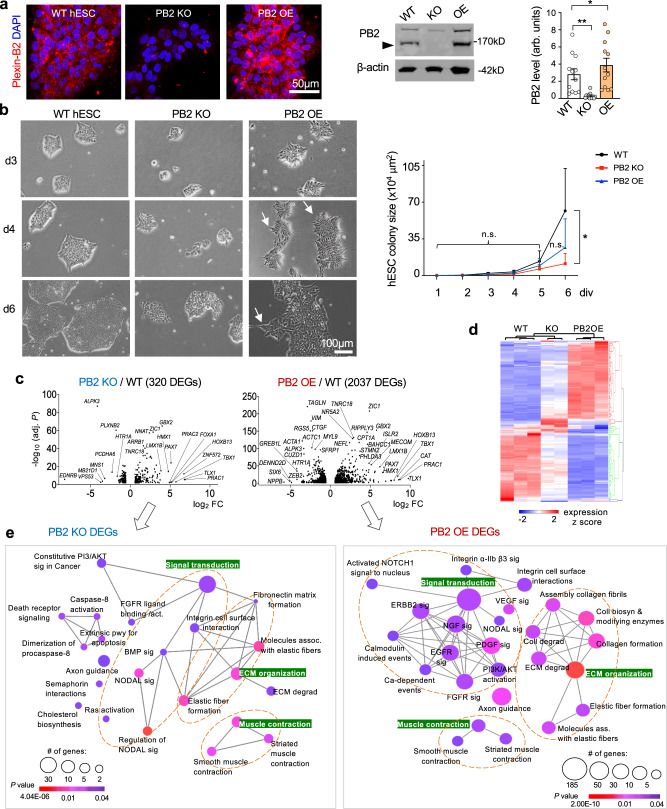
Plexin-B2 controls growth and geometry of hESC colonies via mechanoregulation. **a** IF images (left) show expression of Plexin-B2 (PB2) in wild-type hESC colony (clone WT#1), which was absent in *PLXNB2* KO (clone KO#1) and enhanced in overexpression (OE) hESCs, respectively. Right, Western blot of hESCs probed for Plexin-B2. Arrowhead points to mature Plexin-B2 band at 170 kD. β-actin as a loading control. Quantification, one-way ANOVA followed by Dunnett’s multiple comparisons test. *n* = 12 samples per group. arb.units arbitrary units. *P = 0.0231, **P = *0.0034*. Data represent mean ± SEM. **b** Phase-contrast images of the indicated hESC colonies show similar initial growth kinetics after passage, but by day 6, *PLXNB2* KO colonies appeared smaller (clone #1 for each WT and KO shown). Arrows point to spiky contours of OE colonies. Quantification of colony size is shown on right. Two-way ANOVA followed by Tukey’s post hoc test. For WT colonies, *n* = 14, 14, 9, 10, 18, 7 for day 1–6, respectively; for *PLXNB2 KO*, *n* = 19, 14, 12, 14, 29, 25; and for *PLXNB2 OE*, *n* = 25, 15, 11, 17, 26, 23 colonies, from three independent cultures. *P = 0.0261; n.s. not significant. Data represent mean ± SEM. **c** Volcano plots show differentially expressed genes (DEGs) of *PLXNB2* KO or OE hESCs relative to WT (cut-off thresholds: fold change >2 and *P* < 0.01). Top DEGs are labeled. The *P* values were computed by the DESeq2 package using the Wald test. **d** Heatmap shows unsupervised hierarchical clustering of the DEGs in different genotype conditions (fold change ≥2 between genotypes for the DEGs). **e** Geneset network view of enriched Reactome terms for DEGs in *PLXNB2* KO or OE hESCs relative to WT. Note that gene pathways related to cell biomechanics were highly represented, as were many growth factor signaling pathways, such as EGF and FGF, that are critical for stem cell proliferation and differentiation. Highlighted in green are three major enriched pathways for the DEGs. The *P* values of node connectivity were computed by the Mann–Whitney–Wilcoxon test.

To better understand the role of Plexin-B2 in a multicellular organization, we generated hESCs with *PLXNB2* knockout (KO) by CRISPR/Cas9 gene editing, or Plexin-B2 overexpressing (OE) by lentiviral transduction. Western blot (WB) and immunofluorescence (IF) confirmed loss or gain of Plexin-B2 expression, respectively (Fig. 1a), while DNA sequencing confirmed biallelic CRISPR deletion mutations (Fig. S1a). Introducing a CRISPR-resistant rescue vector restored Plexin-B2 expression in KO cells (Fig. S1b). All CRISPR engineered hESCs displayed normal karyotypes (Fig. S1c).

hESCs under standard culture conditions resemble epiblast, an epithelial layer of pluripotent stem cells (PSCs) in the egg cylinder stage of mammalian embryos^38^. We found that after passage at low density, the initial growth kinetics of hESCs appeared similar among the WT, *PLXNB2* KO, or OE conditions, but by day 6, the KO colonies became smaller than the WT or OE counterparts (Figs. 1b and S1d). Consistently, 5-ethynyl-2′-deoxyuridine (EdU) pulsing labeled fewer cells in the S phase in KO colonies than in WT (Fig. S1e). Reexpressing Plexin-B2 rescued the phenotype, confirming the specificity of the CRISPR-KO approach, while Plexin-B2 OE did not further elevate the high proliferative state of hESCs (Fig. S1e, f). Cell survival was not overtly affected by Plexin-B2 KO, shown by IF for apoptosis marker cleaved caspase 3 (Fig. S1g). Quantitative analysis of the relationship between colony size and Plexin-B2 expression levels showed that Plexin-B2 expression remained constant during colony expansion (Fig. S1h). Intriguingly, we noted that the colony geometry was affected in mutant colonies, in particular, the *PLXNB2* OE colonies displayed spiky contours along the colony edge, in drastic contrast to the smooth border typically seen in WT colonies (Fig. 1b). Together, these phenotypes indicate that Plexin-B2 critically regulates the colony expansion and geometry of hESCs.

### Plexin-B2 affects gene expression concerning cell contraction and ECM interaction

To understand how guidance receptor Plexin-B2 controls colony expansion and geometry on a molecular level, we compared the transcriptomes of *PLXNB2* KO and OE hESCs relative to WT by RNA-sequencing (RNA-seq). Bioinformatic analysis revealed 320 differentially expressed genes (DEGs) between *PLXNB2* KO and WT, and 2037 DEGs between OE and WT hESCs (Figs. 1c, d, and S2c).

Geneset network analysis showed that the enriched Reactome pathways in both sets of DEGs mainly concerned muscle contraction, elastic fiber formation, ECM organization, and integrin signaling (Fig. 1e). Pathway enrichment analyses further indicated that Plexin-B2 manipulations affected Rap1 signaling, Hippo signaling, and mechanoregulation of YAP/TAZ (Fig. S2a, b). These results suggest that Plexin-B2 may affect colony formation by controlling contractile and adhesive properties, which in turn impacts stem cell physiology. Indeed, bioinformatics analyses revealed that multiple signaling pathways critical for stem cell functions including EGFR, FGFR, MAPK, ERK1/2, Wnt/calcium, and focal adhesion/PI3K/AKT pathways, were all affected by Plexin-B2 manipulations (Figs. 1e and S2a, b).

We next intersected the two sets of DEGs (*PLXNB2* KO or *PLXNB2* OE vs. WT), which revealed 272 common DEGs, mainly concerning axon guidance, contractility, ECM interactions, Ras and Rap signaling, as well as NODAL and BMP signaling (Fig. S2c, d). In regard to nonoverlapping DEGs, Plexin-B2 OE specific DEGs (1765 genes) were enriched for receptor tyrosine kinase (RTK) signaling, including PDGF, EGFR, NGF, VEGF, FGF, and ERBB2 signaling (Fig. S2c). This agrees with the known interactions of Plexins and RTKs^39^. In contrast, Plexin-B2 KO-specific DEGs (48 genes) were enriched for genes related to neuronal functions, e.g., neurotransmitter clearance, metabolism of serotonin, or dopamine degradation, suggesting neuronal lineage commitment of KO cells.

Interestingly, a majority of the common DEGs exhibited transcriptional changes in the same direction in both KO and OE hESCs relative to WT (Fig. S2d), suggesting that the gene expression changes likely reflect secondary adaption in response to altered cell biomechanics rather than direct transcriptional regulation by Plexin-B2 signaling. A small number of DEGs displayed opposite directionality of the transcriptional changes in KO and OE cells (Fig. S2d), including genes regulating stem cell function and matrix interactions, e.g., *TNC* (Tenascin C), an ECM gene involved in neuronal differentiation^40^, and *CER1* (Cerberus1), a BMP antagonist essential for inhibiting nonneural differentiation of hESCs, as well as *CCDC141* (coiled-coil domain containing 141), which inhibits cellular adhesion to fibronectin (FN), and *TACR3* (Tachykinin receptor 3), a G protein-coupled receptor that regulates neuronal excitation and smooth muscle contraction.

### Plexin-B2 orchestrates actomyosin network and cytoskeletal tension during hESC colony formation

**Fig. 2:**
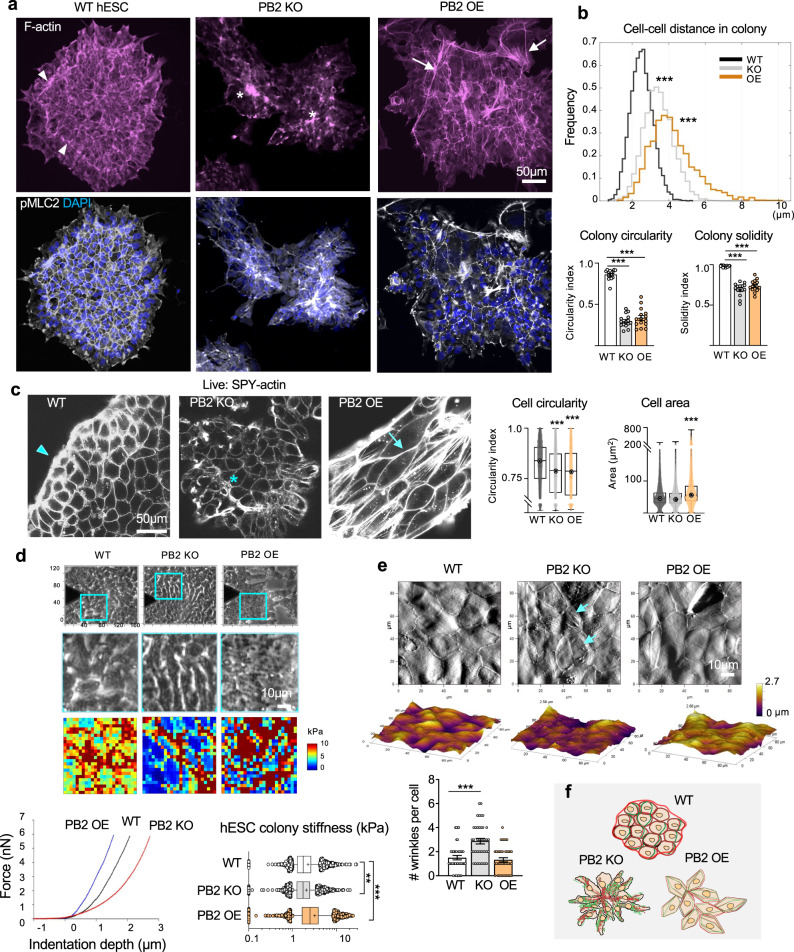
Plexin-B2 orchestrates mechanotension and actomyosin network in hESC colonies. **a** IF images of hESC colonies stained for F-actin (Alexa 568 phalloidin) and pMLC2 show differences in cell organization, colony geometry, and actomyosin network. Arrowheads point to the peripheral actomyosin band in the WT colony, which was disrupted in *PLXNB2* KO or OE mutants. Asterisks denote abnormal cell clusters in the KO colony. Arrows point to stretched contours of the OE colony, often associated with F-actin stress fibers. DAPI for nuclear staining. **b** Top, histograms show the distributions of distances between neighboring cells within hESC colonies (distances between nucleus centroids). Note the wider distribution of cellular distances in KO or OE colonies than in WT, reflective of disorganization. Bottom, quantification of colony geometry (circularity index and solidity). For cell distance histogram, Kruskal–Wallis test followed by Dunn’s multiple comparisons test, *n* = 7646 nuclei for WT, 4478 for *PLXNB2* KO, and 2964 for *PLXNB2* OE. ***P < 0.0001. For colony geometries, one-way ANOVA followed by Dunnett’s multiple comparisons test, *n* = 15 colonies per group. ***P *<* 0.0001. Data represent mean ± SEM. **c** Live-cell imaging of hESC colonies show Plexin-B2-dependent differences in cell morphology, cell organization, and cortical F-actin visualized with SPY555-Actin probe. Arrowhead points to the peripheral F-actin band in WT colony, an asterisk denotes abnormal cell cluster in *PLXNB2* KO colony (clone KO#2), and arrow points to tensed cortical F-actin and stretched junction borders in *PLXNB2* OE colony. High content quantifications of cell shape (circularity index) and cell area are shown. Box plots show 25–75% quantiles, minimal and maximal values (whiskers), and median (bullseye). Kruskal–Wallis test followed by Dunn’s multiple comparisons test. For circularity: WT, *n* = 7878 cells; *PLXNB2* KO, *n* = 5471, and *PLXNB2* OE, *n* = 3904. For area: WT, *n* = 9725 cells; *PLXNB2* KO, *n* = 5615, and *PLXNB2* OE *n* = 4051. ***P *<* 0.0001. Data represent mean ± SEM. **d** Top panel, phase-contrast images show areas in hESC colonies (cyan boxes) measured by AFM elastography. Middle panels, side-by-side magnified phase-contrast images, and corresponding AFM stiffness heatmaps. Bottom, representative AFM indentation curves from elastography measurements (left) and box plots of hESC colony stiffness (right). Box plots show median, 25–75% quantiles and top and bottom 5% data points; mean values indicated by a cross sign. Kruskal–Wallis test followed by Dunn’s multiple comparisons test. Data collected from *n* = 7 samples for WT, *n* = 6 for *PLXNB2* KO, and *n* = 7 for *PLXNB2* OE. **P = 0.0032, ***P *<* 0.0001. **e** Top, AFM topography images show increased surface wrinkles in *PLXNB2* KO cells (arrows), but more stretched surfaces of *PLXNB2 OE* cells compared to WT hESCs. Bottom, quantification of the number of surface wrinkles per cell. One-way ANOVA followed by Dunnett’s multiple comparisons test. *n* = 40 cells analyzed per group. ***P *<* 0.0001. Data represent mean ± SEM. **f** Schematic depiction of cell morphology, cell organization, actomyosin network, and colony geometry as regulated by Plexin-B2 during hESC self-assembly. Red lines indicate F-actin and green lines myosin.

The transcriptomics results indicated cell mechanics as a potential mechanism underlying Plexin-B2’s function in hESCs. We, therefore, examined the F-actin networks in hESC colonies by phalloidin staining (Fig. 2a). In WT colonies, we observed a uniform cobblestone-like cortical F-actin network in the colony interior, and a prominent F-actin band spanning multiple cells along colony periphery, consistent with higher in-plane traction forces at colony edge^6^. In comparison, the organization of F-actin networks was severely disrupted by Plexin-B2 KO or OE, albeit in different ways (Fig. 2a). In the KO colonies, we found that mutant cells tended to aggregate into small cell clusters, with F-actin accumulated at the center, while the peripheral F-actin band was absent, signifying dysregulation of cytoskeletal tension during colony formation. In the OE colonies, cells at the colony periphery were highly disorganized, forming spiky contours, and F-actin distribution was in disarray throughout the colonies, with many cells showing strengthened cortical F-actin and stretched cell borders. Indeed, quantification clearly demonstrated a more homogenous cellular arrangement in WT colonies than in mutants (Fig. 2b). As tissue tension is primarily generated by actomyosin contraction and initiated by phosphorylated myosin light chain 2 (pMLC2)^9,41^, we examined pMLC2 expression, which displayed marked disarray in mutant colonies, mirroring the F-actin patterns (Fig. 2a). Thus, Plexin-B2 has a critical role in organizing the actomyosin network and tensional forces during hESC colony formation.

To examine cell morphology and F-actin networks with a higher spatiotemporal resolution during colony formation, we performed time-lapse live-cell imaging, using SPY-actin to label F-actin^42^. We found that *PLXNB2* OE cells assumed a striking angular morphology with stretched cortical F-actin, taut cell borders, and larger cell area as compared to WT hESCs, whereas *PLXNB2* KO cells exhibited less cortical F-actin, a more rounded shape, but comparable size relative to WT (Figs. 2c and S3a). As the cytoskeleton configuration may affect the size and arrangement of organelles, we compared the shape and size of nuclei in hESC colonies and found that *PLXNB2* KO hESCs had smaller nuclei than WT cells, whereas *PLXNB2* OE cells had more stretched and larger nuclei than WT cells (Fig. S3b).

Remarkably, live videography also revealed that hESCs were highly motile, with constant collisions between small cell clusters before they coalesced into larger colonies, and during this process, cells collectively organized their cortical F-actin networks into a cobblestone-like pattern in the colony interior and a prominent circumferential F-actin band spanning multiple cells at colony periphery (Supplementary Movie 1); this orchestration was severely disrupted by Plexin-B2 perturbations. Specifically, upon collision, *PLXNB2* OE cells maintained an angular morphology and appeared sluggish in reorganizing F-actin networks, leading to stretched junctional borders and a spiky contour in nascent colonies. In contrast, *PLXNB2* KO cells tended to aggregate into smaller clusters with a central accumulation of F-actin; upon collision, cells in the colony center remained disorganized, while the peripheral F-actin band failed to form.

We further analyzed cell motility during colony expansion by live-cell imaging, which showed that cells at the colony periphery migrated faster on average than those in the colony interior (Fig. S3c). Interestingly, the movement of cells at colony edge in Plexin-B2 OE conditions was highest among the three genotypes.

To gain temporal control, we introduced two independent doxycycline (Dox)-inducible shRNAs against *PLXNB2* into hESCs (Fig. S3d). As in KO, Dox-induced Plexin-B2 knockdown (KD) resulted in disorganization of the actomyosin network and dissipation of the peripheral F-actin band (Fig. S3e, f). The altered colony geometry and cellular misalignment at the edges of KD colonies could be reversed by 24 h of Dox washout, indicating rapid cytoskeletal reorganization under the control of Plexin-B2 (Fig. S3g).

To directly gauge mechanical properties of hESC colonies in dependence of Plexin-B2, we conducted microindentation testing (elastography) using atomic force microscopy (AFM)^43^. Results showed that *PLXNB2* KO cells had lower stiffness (mean 2.0 kPa) than WT (2.2 kPa), but *PLXNB2* OE cells had higher stiffness (3.1 kPa) (Fig. 2d). We also performed 3D contact imaging by AFM to scan cell surface topologies, which revealed rougher surfaces for KO colonies, but smoother surfaces for OE colonies (Fig. 2e). Together, live-cell imaging and AFM measurements support the role of Plexin-B2 in orchestrating actomyosin organization and cytoskeletal tension during hESC colony formation (Fig. 2f).

### Plexin-B2 controls actomyosin contractility and cell stiffness in hNPCs

**Fig. 3:**
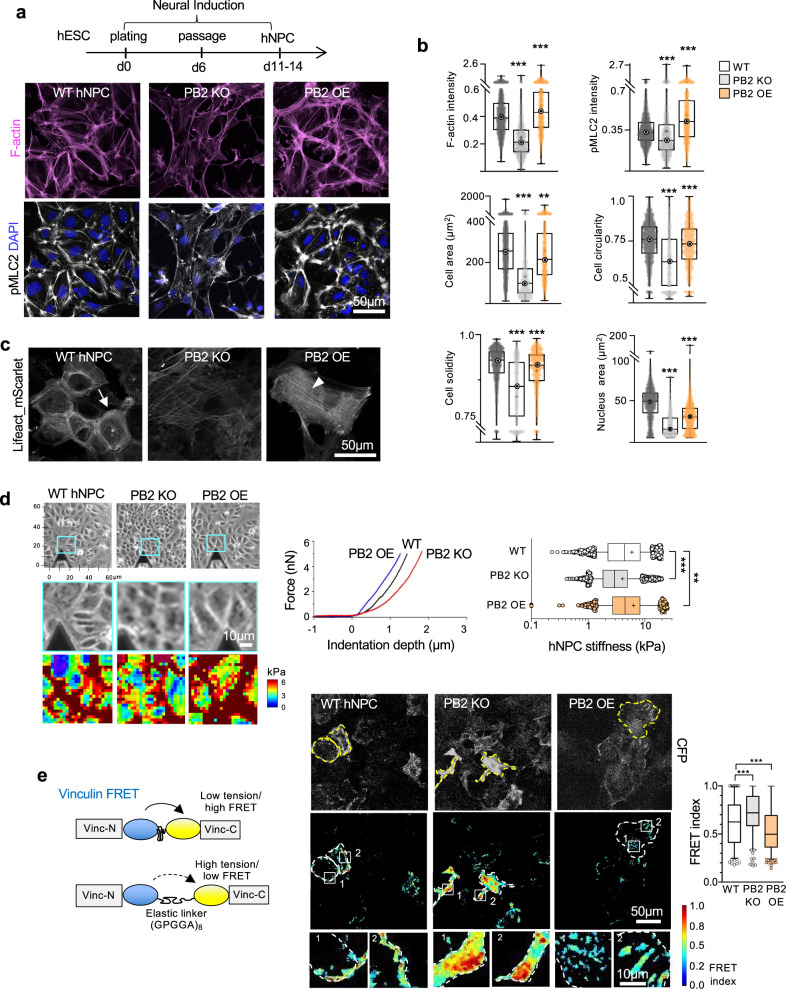
Plexin-B2 regulates actomyosin contractility and cell stiffness in hNPCs. **a** Top, timeline of neural induction to generate hESC-derived hNPCs. Bottom, confocal IF images of hNPCs show differences in F-actin (phalloidin) and pMLC2 in *PLXNB2* KO and OE compared to WT cells. **b** Box plots show high content quantifications of F-actin and pMLC2 intensity, as well as geometries (cell spreading area, circularity, and solidity) and nuclei area of WT, *PLXNB2* KO, and OE hNPCs. Box plots show 25–75% quantiles, minimal and maximal values (whiskers), median (bullseye), and mean (cross sign). For intensities and cell geometries: WT, *n* = 2123 cells; *PLXNB2* KO, *n* = 930, and *PLXNB2* OE, *n* = 1172. For nuclei area: WT, *n* = 2265 nuclei; *PLXNB2* KO, *n* = 1275, and *PLXNB2* OE, *n* = 1720. Data collected from 30 random fields per group. Kruskal–Wallis test followed by Dunn’s multiple comparisons test. **P = 0.0037 and ***P *<* 0.0001. **c** Live-cell imaging of hNPCs shows different F-actin distribution patterns in mutant hNPCs as revealed by Lifeact_mScarlet. Arrow points to cortical F-actin in WT hNPCs, which was reduced in *PLXNB2* KO cells. Arrowhead points to stress fibers in *PLXNB2* OE hNPCs. **d** Top, phase-contrast images depicting areas of hNPC cultures (cyan boxes) that were measured by AFM elastography. Bottom, side-by-side magnified phase-contrast images and corresponding AFM stiffness heatmaps. Right, representative AFM indentation curves from elastography measurements of WT, *PLXNB2* KO, and OE hNPCs (left), and right, box plots quantification of cell stiffness (median, 25–75% quantiles, median, and top and bottom 5% data points, with mean values indicated by cross sign). Kruskal–Wallis test followed by Dunn’s multiple comparisons test. Data collected from *n* = 3 samples per group. **P = 0.0013, ***P *<* 0.0001. **e** Left, diagram of vinculin-based FRET tension sensor (CFP and YFP proteins connected by elastic linker) to gauge tensile forces at focal adhesions (FAs). Stretching of the linker reduces FRET signals. Middle, representative images of normalized FRET signals in hNPCs. Note that transient transfection leads to variable expression of FRET construct in cells, as visualized by CFP baseline fluorescence. Right, quantification of FRET index shown as box plots (median, 25–75% quantiles, and top or bottom 5% data points). Kruskal–Wallis test followed by Dunn’s multiple comparisons test. Data collected from 175 FAs for WT, 191 FAs for *PLXNB2* KO, and 268 FAs for *PLXNB2* OE. ***P = 0.0001 (WT vs PB2 KO) and ***P = 0.0008 (WT vs PB2 OE).

Given a critical role of Plexin-B2 in neural tube closure and neuroprogenitor migration^12^, we next investigated whether Plexin-B2 controls biomechanical properties of hNPCs. To this end, we derived hNPCs from WT, *PLXNB2* KO, or OE hESCs and analyzed actomyosin patterns. Similar to the results from hESCs, cortical actomyosin accumulation was reduced in *PLXNB2* KO hNPCs relative to WT, but increased in OE cells (Fig. 3a, b). Morphometric quantifications confirmed alterations of cell shape as a result of Plexin-B2 manipulations (Fig. 3b). Interestingly, the nuclei of both *PLXNB2* KO and OE hNPCs were on average smaller than those of WT counterparts (Fig. 3b), which may be associated with an aberrant differentiation state.

We next performed live-cell imaging of hNPCs labeled with mScarlet-Lifeact, a fluorescent F-actin probe^44^. We detected reduced cortical F-actin in *PLXNB2* KO hNPCs, but a marked elevation in OE cells that contained actin stress-fibers and angular morphology (Fig. 3c). AFM elastography demonstrated that *PLXNB2* OE NPCs exhibited higher cell stiffness (mean 6.3 kPa) than WT hNPCs (5.9 kPa); conversely, cell stiffness was lower for KO hNPCs (4.0 kPa) (Fig. 3d).

To measure tension exerted on FAs in hNPCs, we introduced a FRET tension sensor based on vinculin, an intracellular linker of adhesion sites with cytoskeleton^45^ (Fig. 3e). Analysis of the FRET signals revealed that the forces exerted on FAs were indeed lower in *PLXNB2* KO than in WT hNPCs, but higher in OE hNPCs (Fig. 3e).

### Plexin-B2 affects the organization of adhesion complexes in epithelial colonies and 3D hESC aggregates

**Fig. 4:**
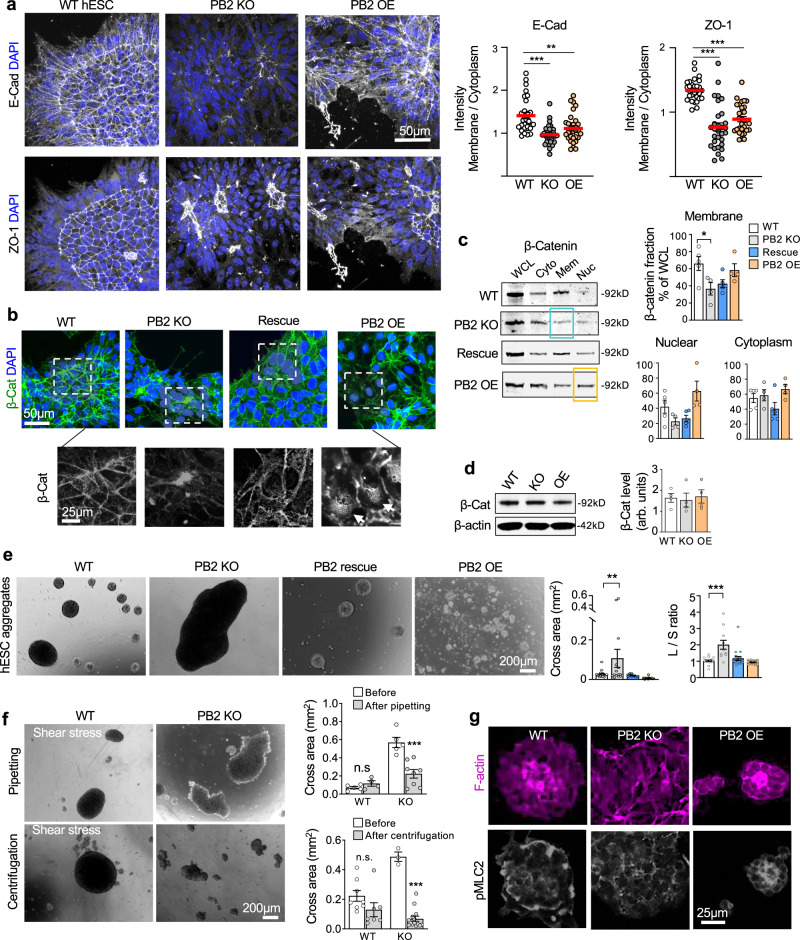
Plexin-B2 influences cell–cell adhesion in multicellular organization of hESCs. **a** Confocal IF images show altered junction recruitment of E-cadherin and ZO-1 in mutant hESC colonies in contrast to the organized pattern in WT colony. Data points in quantification shown on right represent boundary/cytoplasm ratios of E-cadherin and ZO-1 immunointensities. *n* = 30 cells per group. One-way ANOVA followed by Dunnett’s multiple comparisons test. **P = 0.0016 and ***P *<* 0.0001. Red lines represent mean values. **b** Confocal IF images reveal redistribution of β-catenin in mutant hESC colonies. Enlarged images of boxed areas are shown in bottom panels, highlighting β-catenin membrane localization in WT cells, which was shifted to the center of cell clusters in *PLXNB2* KO. Arrows point to increased nuclear β-catenin in *PLXNB2* OE cells. **c** WBs of cell fractions show a shift of membrane-associated (Mem) β-catenin to the cytoplasm (Cyto) in *PLXNB2* KO hESCs (highlighted by blue box), which was rescued by CRISPR-resistant Plexin-B2. *PLXNB2* OE resulted in increased nuclear (Nuc) β-catenin (highlighted by orange box). WCL whole cell lysate. Quantifications are shown to the right. One-way ANOVA followed by Tukey’s multiple comparisons test. *n* = 5 for WT, *n* = 4 for *PLXNB2* KO, *n* = 5 for Rescue, and *n* = 4 for *PLXNB2* OE. *P = 0.0183. Data represent mean ± SEM. **d** WB and quantification show comparable levels of total β-catenin in WT and *PLXNB2* mutant hESCs. β-actin served as a loading control. arb.units arbitrary units. One-way ANOVA followed by Tukey’s multiple comparisons test. *n* = 4 samples per group. Data represent mean ± SEM. **e** Floating 3D hESC aggregates after 48 h culture in low adherence conditions. Quantifications of cross area and shape (L/S ratio, longest vs. shortest diameter) of aggregates are shown on the right. PB2 KO clone #1. One-way ANOVA followed by Dunnett’s multiple comparisons test. For cross area, *n* = 20 aggregates for WT, *n* = 15 for *PLXNB2* KO, *n* = 9 for Rescue, and *n* = 37 for *PLXNB2* OE. For L/S ratio, *n* = 19 aggregates for WT, *n* = 11 for *PLXNB2* KO, *n* = 19 for Rescue, and *n* = 28 for *PLXNB2* OE from 3–5 fields. **P = 0.0071, ***P *<* 0.0001. Data represent mean ± SEM. **f** Images and quantifications show the reduced capability of *PLXNB2* KO hESC aggregates to withstand shear forces applied by pipetting or low-speed centrifugation compared to WT. Two-sided unpaired *t*-test to compare within the groups. For pipetting: WT, *n* = 5 aggregates before and *n* = 4 after; *PLXNB2* KO, *n* = 5 before and *n* = 8 after. ***P = 0.0008. For centrifugation: WT, *n* = 8 before and *n* = 7 after; *PLXNB2* KO, *n* = 3 aggregates before and *n* = 14 after. ***P *<* 0.0001; n.s. not significant. Data represent mean ± SEM. **g** IHC images of cross section of 3D hESC aggregates show that *PLXNB2* KO resulted in cellular disarray and disorganized actomyosin networks, whereas *PLXNB2* OE led to enhanced actomyosin compact.

So far, we showed that Plexin-B2 controls cell stiffness and actomyosin organization in the multicellular organization. As contractile forces are transmitted to cell–cell and cell–matrix junctions to maintain tissue cohesion^3^, we next studied the impact of Plexin-B2 on the organization of adhesion complexes in hESC colonies. We observed that while E-cadherin and ZO-1 displayed a uniform cobblestone-like network in WT colonies, both were severely disrupted in *PLXNB2* mutant colonies (Figs. 4a and S4a), concordant with the cellular disarray and disorganized actomyosin networks.

In hESCs, β-catenin forms a complex with E-cadherin^46^, which connects to actin cytoskeleton to mediate cell–cell adhesion and pluripotency^8^. Indeed, in WT hESCs, β-catenin displayed a similar cobblestone-like pattern as E-cadherin (Fig. 4b). However, Plexin-B2-deficiency resulted in reduced membrane association of β-catenin and redistribution towards the center of cell clusters, a phenotype partially rescued by Plexin-B2 re-expression; in contrast, Plexin-B2 OE led to an increased nuclear fraction of β-catenin (Fig. 4b). These phenotypes are likely a consequence of the disorganized membrane clustering of E-cadherin in hESC colonies. WB confirmed a shift in subcellular localization of β-catenin (Fig. 4c), while the total protein levels remained similar in all three groups (Fig. 4d). The subcellular fractionation approach was verified by probing for markers of nuclear, cytoplasmic, and membrane fractions, respectively (Fig. S4b).

We next examined the impact of Plexin-B2 on the self-assembly of hESCs in a 3D context. When cultured in low-attachment dishes, WT hESCs spontaneously aggregated into compact spheroids of ~200–500 μm diameter by 48 h; strikingly, *PLXNB2* KO cells formed larger but irregularly shaped aggregates, measuring up to 1.5 mm in length, a phenotype rescued by Plexin-B2 reexpression (Fig. 4e). Conversely, *PLXNB2* OE resulted in smaller but more compact aggregates (~18–60 μm in diameter), reflective of a heightened contractile state. As a gauge of tissue cohesion or adhesive forces between cells, we tested the ability of hESC aggregates to resist external shear stress applied either through gentle pipetting or by centrifugation^47^. Results showed that whereas WT aggregates maintained the compact spheroid shape under shear stress, the large *PLXNB2* KO aggregates appeared easily broken up into smaller pieces with uneven shapes, consistent with reduced tissue cohesion (Fig. 4f).

Concordantly, IF staining of cross sections of the WT spheroids revealed a high concentration of organized F-actin networks in the center, surrounded by a rim of pMLC2 at the periphery, whereas both F-actin and pMLC2 appeared more compact in *PLXNB2* OE spheroids, but looser and in disarray in KO aggregates (Fig. 4g). Actomyosin contraction can affect FN assembly in multicellular organization^48,49^. Indeed, as another sign of disorganized actomyosin network, the gradient of FN fibrils extending from core to periphery seen in WT spheroids appeared more compact in *PLXNB2* OE spheroids, but in marked disarray in KO aggregates (Fig. S4c).

As an alternative approach to gauge cell–cell cohesion, we also examined aggregation of hESCs and hNPCs in a hanging drop assay^50^. We detected reduced or enhanced compaction of hanging drop aggregates of *PLXNB2* KO or OE cells, respectively, relative to WT counterparts (Fig. S4d, e).

### Plexin-B2 affects focal adhesion maturation in hESC colony and hNPCs

**Fig. 5:**
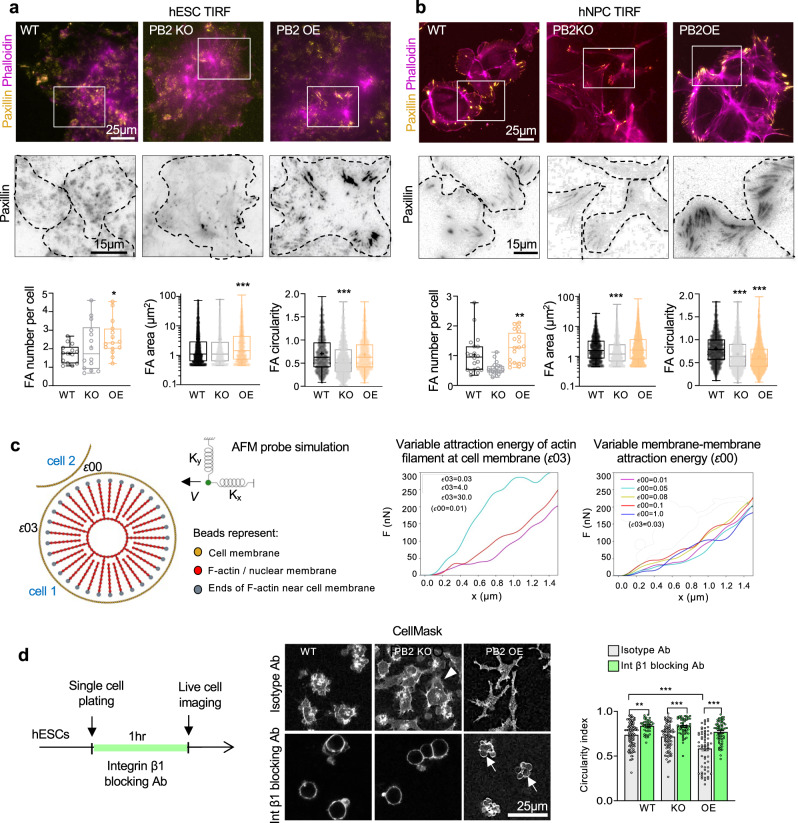
Plexin-B2 influences cell–matrix adhesion and controls contractility in hESC colonies. **a**, **b** TIRF microscopy images and quantification show altered number, size, and shape of focal adhesions (FAs) in *PLXNB2* mutant hESC colonies (**a**) and hNPCs (**b**) compared to wild-type (WT) cells. Cells were stained for FA marker paxillin and F-actin (phalloidin). Enlarged images of boxed areas are shown below in inverted black and white, with dashed lines outlining individual cells. Box plots depict 25–75% quantiles, minimal and maximal values (whiskers), median (midline), and mean (cross signs). For hESCs: FA number, *n* = 14–15 cells per group. FA area, *n* = 2271 FAs for WT, *n* = 1042 for PB2 KO, and *n* = 978 for PB2 OE. FA circularity, *n* = 1042 FAs for WT, *n* = 1042 for PB2 KO, and *n* = 978 for PB2 OE. *P = 0.0397, ***P *<* 0.0001. For hNPCs: FA number, *n* = 20 cells per group. FA area and circularity, *n* = 1829 FAs for WT, *n* = 2385 for PB2 KO, and *n* = 4528 for PB2 OE. Kruskal–Wallis test followed by Dunn’s multiple comparisons test. **P = 0.0084, ***P *<* 0.0001. **c** Molecular dynamics mathematical simulation. Left, components of simulation based on a bead-spring model. The tip of the simulated AFM probe is depicted as a green sphere, with elastic constants K_x_ and K_y_ in the x and y directions, respectively. Right, graphs of force as a function of distance traveled by the simulated AFM probe in two different scenarios. Left, when varying the actin-membrane attraction energy that simulates increasing contractility, the response of the force to the virtual AFM probe resembles the results of the experimental AFM data in accordance to Plexin-B2 activity (see Fig. 2). Such result is not seen when varying membrane-membrane attraction energy (right). **d** Left, experimental design for live-cell imaging. Middle, images of hESCs labeled with CellMask reveal distinct cell morphology when exposed to a function-blocking antibody against integrin β1 (P5D2) or an isotype control IgG. Arrowhead points to cellular protrusion from *PLXNB2* KO hESCs. Arrows point to cell membrane blebbing of *PLXNB2* OE hESCs after integrin blockade. Quantification for cell roundness (circularity index) is shown on right. One-way ANOVA followed by Tukey’s multiple comparisons test. For isotype IgG treatment: *n* = 100 cells for WT, *n* = 80 cells for PB2 KO, and *n* = 63 for PB2 OE. For integrin β1 treatment: *n* = 41 cells for WT, *n* = 54 for PB2 KO, and *n* = 63 cells for PB2 OE. **P = 0.0025 and ***P *<* 0.0001. Data represent mean ± SEM.

We next examined the impact of Plexin-B2-mediated actomyosin tension on the interface of cytoskeleton and matrix. We performed total internal reflection fluorescence (TIRF) microscopy for morphometric analysis of FAs visualized by paxillin IF (Fig. 5a, b). In both hESCs and hNPCs, we observed increased numbers of FAs with *PLXNB2* OE. In addition, both the size and the shape of FAs were also altered, with generally larger FAs with *PLXNB2* OE, and less circular ones with KO, although there were variabilities between hESCs and hNPCs (Fig. 5a, b).

Next, we investigated the levels of phosphorylated focal adhesion kinase (pFAK) as an indicator of stable FAs. Confocal microscopy revealed diminished levels of pFAK in Plexin-B2-deficient hESC colonies, but enhanced accumulation in Plexin-B2 OE, both in disarray in contrast to the cobblestone-like pattern seen in WT (Fig. S5a). In hNPCs, Plexin-B2 OE also led to increased pFAK levels (Fig. S5b). Together, these data support the notion that FA maturation is affected by different tensional forces as mediated by Plexin-B2.

In Drosophila sensory neurons, Plexin-B forms a complex with integrin β and stabilizes it on the cell surface to regulate self-avoidance and dendritic tiling^51^. Our microscopy analysis of hESC colonies revealed analogous results, with active integrin β1 immunosignals attenuated in *PLXNB2* KO, but enhanced in OE colonies (Fig. S5c). Of note, WB showed that only the intracellular localization, but not the total protein level of integrin β1 was affected by *PLXNB2* KO (Fig. S5d). A similar loss of active integrin β1 was observed in *PLXNB2* KO hNPCs (Fig. S5e). Hence, Plexin-B2 controls actomyosin tension and maturation of junctional adhesions in multicellular organization (Fig. S5f).

### Plexin-B2 primarily controls contractility in hESC colony with consequent changes in adhesion

Our next question pertains to whether Plexin-B2 primarily controls actomyosin contractility or adhesive strength, two interconnected processes in a multicellular organization. To address this question, we performed mathematical simulations of cell mechanics using the molecular dynamics approach^52,53^. We first developed a membrane model that reproduces the main elements of cellular mechanical components based on the coarse-grained bead-spring model, wherein cell membrane, nuclear membrane, and actin filaments were modeled as beads connected by springs; and a virtual AFM tip was represented as a sphere anchored by two springs (Fig. 5c). We simulated how cell stiffness might behave in response to either: (i) modulation of actomyosin contractile forces, as simulated by altering the attraction energy between actin filament heads and cell membrane (energy *ɛ*03) or (ii) varying cell adhesion, as simulated by altering the interaction energy between neighboring cell membranes (*ɛ*00). We tested two scenarios: variable *ɛ*03 with fixed *ɛ*00, or variable *ɛ*00 with fixed *ɛ*03 (Fig. 5c and Supplementary Movies 2, 3). The simulation results from the first scenario—i.e., modulating actomyosin contractile forces—aligned better with our experimental AFM data. Hence, mathematical simulations support the model that Plexin-B2 primarily controls actomyosin contractility, with secondary effects on cell adhesiveness during the multicellular organization.

To corroborate the simulation results, we assayed the contractile properties of hESCs in the single-cell state under adherent vs. non-adherent conditions, thus decoupling actomyosin contraction from cell–cell or cell–matrix adhesion (Fig. 5d). Live-cell imaging revealed that under adherent conditions, individual cells with *PLXNB2* OE assumed a highly branched morphology, whereas KO cells frequently extended cellular protrusions, suggestive of reduced cell–matrix anchorage. We then released cells from matrix anchorage by incubation with an anti-integrin β1 blocking antibody. This resulted in rounding-up of cells, and notably, *PLXNB2* OE cells displayed membrane blebbing, signifying cytoskeletal hypercontractility (Fig. 5d). This agrees with the model that a main function of Plexin-B2 is to promote actomyosin contractility.

To further test this model, we applied WT or *PLXNB2* mutant hESCs pharmacological inhibitors for myosin II (blebbistatin, 10 µM) or Rho-associated coiled-coil-containing kinase (ROCK) (Y-27632, 20 µM)^54^ (Fig. S6a, b). Inhibition of actomyosin contractility by these inhibitors indeed abolished the hypercontractile state of Plexin-B2 OE cells, leading to junction changes resembling the KO phenotypes in all three genotype conditions (Fig. S6b). Exposure to latrunculin (5 µM), which inhibits F-actin assembly, resulted in rounding-up of cells and cell dissociation in all three groups (Fig. S6b). These data support the interconnection of actomyosin contractility and cell adhesion with a primary function of Plexin-B2 in promoting the former. Interestingly, treatment with Y16 (25 µM), a specific inhibitor of the Rho-GEFs, which can function as downstream effectors of Plexin-Bs^55^, caused no significant morphological changes in hESC colonies or in 3D spheroids in all three groups (Fig. S6c, d).

We performed additional mathematical simulations of molecular dynamics to probe the impact of varying actomyosin contractility on cell cohesion in multicellular settings. This was interrogated by calculating the force needed to pull cells from the colony edge or from the colony center. Simulation results indicated that increased cell contractility can strengthen cell–cell cohesiveness (Fig. S6e). Altogether, our results demonstrated that Plexin-B2-deficient hESCs formed less compact and less cohesive colonies, whereas Plexin-B2 OE hESCs formed more adhesive colonies. As both Plexin-B2 KO and OE colonies displayed marked cellular disarray, it indicated that a balanced level of Plexin-B2 activity is required to orchestrate multicellular dynamics.

### Plexin-B2 engages extracellular and Ras-GAP domains to orchestrate cytoskeletal network

**Fig. 6:**
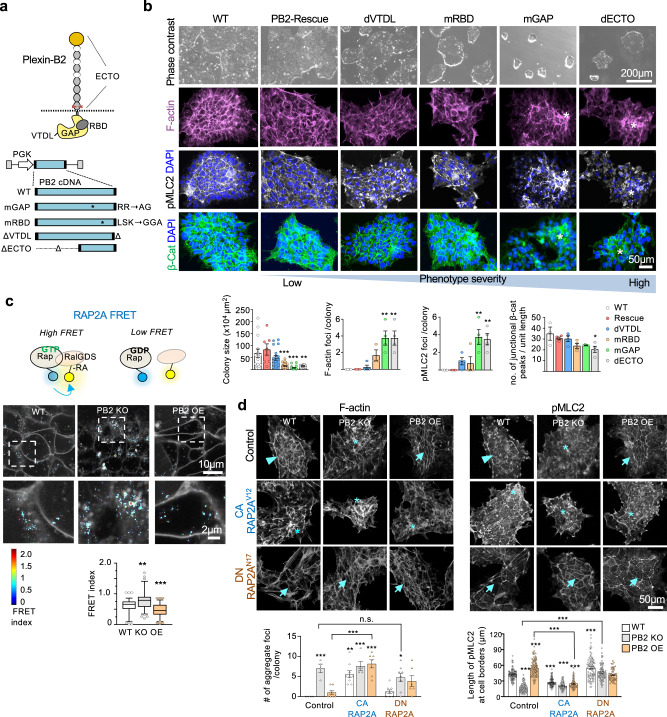
Plexin-B2 signals through RAP1/2 to orchestrate cell mechanics in hESC colonies. **a** Top, Plexin-B2 domain structure: ECTO ectodomain, RBD Rho-binding domain, GAP Ras-GTPase domain, VTDL, four C-terminal amino acids, forming PDZ binding motif. The cleavage site of mature Plexin-B2 is depicted by red arrows. Bottom, lentiviral vectors expressing wild-type (WT) Plexin-B2 or signaling mutants (CRISPR-KO resistant): mGAP mutation in GAP, mRBD mutation in RBD, ΔVTDL deletion of VTDL, ΔECTO deletion of ECTO domain. **b** Phase-contrast and confocal images illustrate the severity of cytoskeletal disarray and β-catenin membrane distribution phenotypes of hESC colonies expressing wild-type or Plexin-B2 signaling mutants (in the background of *PLXNB2* KO). Asterisks denote abnormal cell clusters within the colony. Quantifications are shown below. One-way ANOVA followed by Tukey’s multiple comparisons test. For hESC colony size: *n* = 14 colonies for WT, *n* = 7 for Rescue, *n* = 18 for dVTDL, *n* = 24 for mRBD, *n* = 21 for mGAP, and *n* = 12 for dECTO. **P = 0.0028; ***P < 0.0003 (WT vs mRBD), ***P *<* 0.0001 (WT vs mGAP). For others, *n* = 3–4 for each group. For F-actin foci, **P = 0.002. For pMLC2 foci, **P = 0.0019 (WT vs. mGAP) and **P = 0.0037 (WT vs. dECTO). For junctional β-cat peaks, *P = 0.025. Data represent mean ± SEM. **c** Top, diagram of FRET probes for RAP2A activation status. Middle, representative images of normalized RAP2A FRET signals in WT, *PLXNB2* KO (clone #3), *PLXNB2* OE hESCs. Enlarged images of boxed areas are shown below. Box plots show median, 25–75% quantiles, and top or bottom 5% data points. Kruskal–Wallis test followed by Dunn’s multiple comparisons test. *n* = 98 cell–cell membrane areas for WT; *n* = 89 for PB2 KO; *n* = 85 for *PLXNB2* OE. **P = 0.0013 and ***P *<* 0.0001. **d** Confocal IF images show the effects of RAP2A isoforms on cortical actomyosin network, colony geometry, and cellular organization of hESC colonies as compared to control colonies with no RAP2A expression vector. Arrowheads point to the peripheral F-actin band in WT colony, asterisks denote abnormal cell cluster in *PLXNB2* KO colony, and arrows point to tensed cortical F-actin and stretched junction borders in *PLXNB2* OE colony. Note that constitutively active (CA) RAP2A^V12^ phenocopied Plexin-B2 KO (clone #3), whereas dominant-negative (DN) RAP2A^N17^ phenocopied Plexin-B2 OE. Quantifications of actomyosin aggregate foci per colony and length of pMLC2 at cell borders are shown below. For aggregate foci: control, *n* = 4 colonies for WT, *n* = 4 for PB2 KO, and *n* = 8 for PB2 OE; CA RAP2A^V12^, *n* = 9 for WT, *n* = 5 for PB2 KO, and *n* = 7 for PB2 OE; DN RAP2A^N17^, *n* = 9 for WT, *n* = 8 for PB2 KO, and *n* = 6 for PB2 OE. *P = 0.010, **P = 0.0017, ***P < 0.0001. For pMLC2: control, *n* = 80–100 cell borders for each genotype; CA RAP2A^V12^, *n* = 60–70 for each genotype; DN RAP2A^N17^, WT, *n* = 50–90 for each genotype. ***P *<* 0.0001. One-way ANOVA followed by Dunnett’s post hoc correction to compare to WT group. A two-sided unpaired *t*-test was applied when comparing data from two groups (brackets). Data represent mean ± SEM.

Plexin-B2 can potentially signal through its Ras-GAP domain to inactivate Ras/Rho small GTPases^56–59^, its Rho-binding domain (RBD) that can bind to Rac or Rnd^57,60^, or its C-terminal PDZ binding motif as a docking site for PDZ-Rho-GEFs to activate RhoA^61^ (Fig. 6a). We next dissected which of these Plexin-B2 domains are engaged in the mechanoregulation of multicellular dynamics.

We introduced a series of Plexin-B2 mutants into KO hESCs to conduct domain-specific rescue experiments: Plexin-B2-mGAP (mutation of amino acids required for Ras-GAP activity), -mRBD (critical mutations in RBD domain), -ΔVTDL (deletion of PDZ binding motif), and -ΔECTO (deletion of an extracellular domain) (Fig. 6a). WB and ICC confirmed comparable expression levels of Plexin-B2 signaling mutants in hESCs (Fig. S7a, b).

We found that the Plexin-B2 KO phenotypes—i.e., altered colony size and geometry, disorganized actomyosin and β-catenin networks—could be fully restored by wild-type (WT) Plexin-B2 rescue vector, largely rescued by Plexin-B2-ΔVTDL, partially rescued by Plexin-B2-mRBD, but minimally by Plexin-B2-mGAP and -ΔECTO (Fig. 6b). Hence, the extracellular and the Ras-GAP domains, and to a lesser degree RBD domain, are required for Plexin-B2 mechanoregulatory activity. In contrast, the C-terminal PDZ binding motif appeared dispensable, echoing the results from Rho-GEF inhibitor Y16 (see Fig. S6c, d),

Since the extracellular domain of Plexin-B2 is required to rescue the KO phenotype in the hESC colony, we tested how hESCs respond to Sema4C, a ligand for Plexin-B2. When WT hESCs were exposed to increasing concentrations of Sema4C mixed with Matrigel substrate, colony geometry was perturbed, displaying a contractile appearance with irregular contours, resembling the tensed appearance of *PLXNB2* OE colonies (Fig. S7c). *PLXNB2* OE cells responded to Sema4C similarly as WT cells, but Plexin-B2 KO cells appeared insensitive to Sema4C. Of note, anchorage of Sema4C to the cell membrane or substrate surface seemed critical as applying Sema4C in culture media did not elicit such an effect (Fig. S7d). It is notable that the effect of Sema4C was not identical to Plexin-B2 OE, thus a ligand-independent function of Plexin-B2 or its involvement in homotypic cell–cell adhesion remains a possibility^18^.

### Plexin-B2 signals through RAP1/2 for mechanoregulation

Next, to validate the importance of the Ras-GAP domain of Plexin-B2 during hESC colony formation, we utilized genetic FRET probes to probe the activation status of RAP1/2 by live-cell imaging. Indeed, the FRET probes revealed that *PLXNB2* OE cells showed reduced RAP1A and 2A activation as compared to WT cells, while KO cells displayed enhanced RAP1/2 activation (Figs. 6c and S8a).

To further test the functions of RAP1/2 as downstream effectors of Plexin-B2, we introduced constitutively active (CA) or dominant-negative (DN) isoforms of RAP1B or 2A into hESCs (Figs. 6d and S8b). In all three groups (WT, *PLXNB2* KO, or OE), expression of CA RAP1B or 2A phenocopied Plexin-B2 KO in regards to disorganized actomyosin networks, dissipation of the peripheral F-actin band, and filopodia-like protrusions at colony edge. Conversely, DN RAP1/2 phenocopied Plexin-B2 OE in regards to strengthened cortical F-actin, angular morphology, stretched cell borders, and spiky colony contours in all three genotypes, including Plexin-B2 KO (Figs. 6d and S8b). Additionally, membrane clustering of β-catenin, as well as the proliferation of hESCs, were affected by CA- or DN- Rap1/2 mutants, again phenocopying Plexin-B2 KO or OE, respectively (Fig. S8c, d). Hence, functional data support the engagement of RAP1/2 by Plexin-B2 in the mechanoregulation of the hESC colony.

### Plexin-B2-mediated cytoskeletal tension impacts on YAP/TAZ activity

**Fig. 7:**
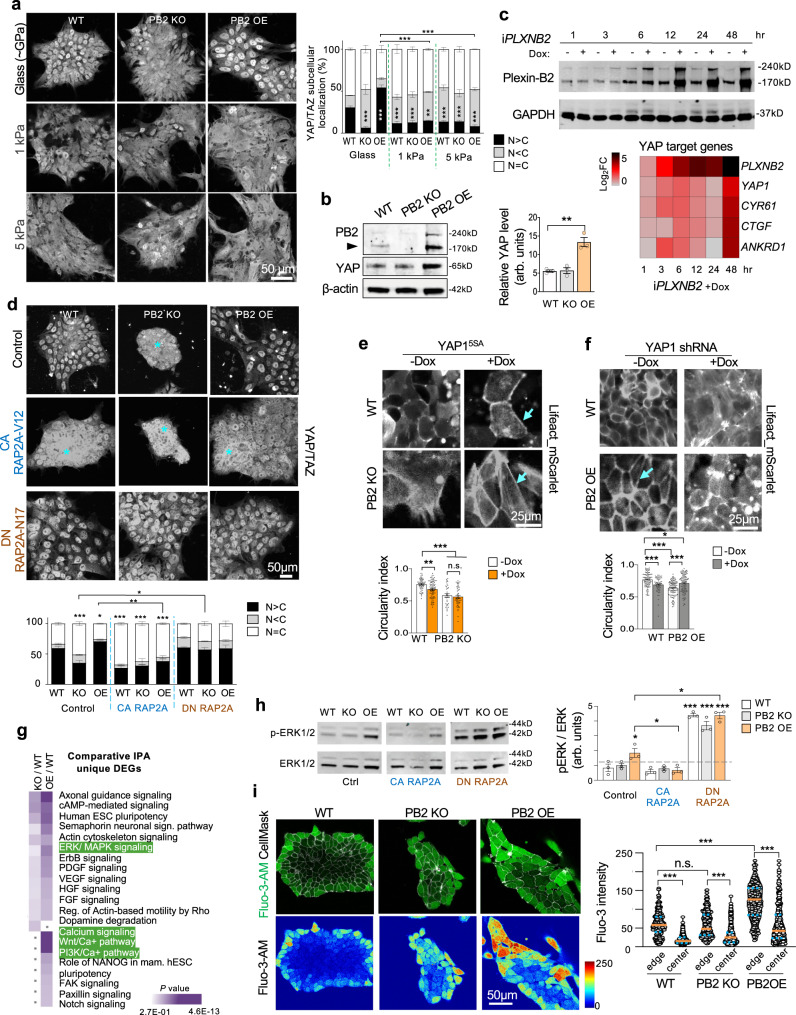
Plexin-B2 engages the YAP mechanotransduction pathway and activates ERK1/2 and calcium signaling. **a** Confocal IF images show reduced nuclear YAP/TAZ in *PLXNB2* KO hESCs, but a cytoplasm-to-nucleus shift in *PLXNB2* OE as compared to WT cells on standard cell culture substrate (glass coverslip coated with Matrigel). Cultures on hydrogels with softer stiffness (1 and 5 kPa) is similar for all genotype groups with a strong reduction of nuclear YAP/TAZ. Quantifications show relative fractions of cells with nuclear YAP/TAZ signals that are greater, equal, or smaller than cytoplasmic signals (N > C, N = C, N < C). Two-way ANOVA followed by Dunnett’s post hoc test to compare to WT. *n* = 115–301 cells for each condition **P = 0.0019, ***P = 0.0001 (WT glass vs KO and OE glass), ***P = 0.0002 (WT glass vs WT 1 kPa), ***P = 0.0004 (WT glass vs KO 1 kPa), ***P = 0.0008 (WT glass vs WT 5 kPa), ***P = 0.0009 (WT glass vs KO 5 kPa), ***P = 0.0001 (WT glass vs OE 5 kPa). A two-sided unpaired *t*-test was applied when comparing data from two groups (brackets). ***P = 0.0002 (OE Glass vs OE 1 kPa, and OE Glass vs OE 5 kPa), Data represent mean ± SEM. **b** WB and quantification show an increased level of total YAP in *PLXNB2* OE cells as compared to WT or *PLXNB2* KO hESCs. Arrowhead points to mature Plexin-B2 band. β-actin served as a loading control. arb.units arbitrary units. One-way ANOVA followed by Tukey’s multiple comparisons test. *n* = 3 samples per group. **P = 0.0017. Data represent mean ± SEM. **c** Top, WB shows time course of Dox-induced expression of Plexin-B2 (i*PLXNB2)* in hESCs. GAPDH served as a loading control. Bottom, heatmap of qRT-PCR data illustrates upregulation of *YAP1* and its target genes *CYR61, CTGF, and ANKRD1* upon Plexin-B2 induction. The heatmap was generated by the mean value from *n* = 3 independent cultures. **d** IF images show the effects of RAP2A isoforms on YAP/TAZ nuclei/cytoplasmic localization in hESC colonies. Note that expression of constitutive active RAP2A^V12^ led to elevated cytoplasmic YAP/TAZ in all three groups, phenocopying Plexin-B2 KO. Quantification shows relative fractions of cells with nuclear YAP/TAZ immunosignals that are greater, equal, or smaller than cytoplasmic signals (N > C, N = C, N < C). Two-way ANOVA followed by Dunnett’s post hoc test to compare to WT group. For control, *n* = 330–466 cells for each genotype; for CA RAP2A^V12^, *n* = 163–233 for each genotype; for DN RAP2A^N17^, *n* = 412–543 for each genotype *P = 0.040 and ***P *<* 0.0001. A two-sided unpaired *t*-test was applied when comparing data from two groups (brackets). *P = 0.015, **P = 0.0012. Data represent mean ± SEM. **e**, **f** Live-cell imaging shows differences in cortical F-actin (visualized by LifeAct_mScarlet) in hESCs with Dox-inducible expression of YAP1^5SA^ (**e**) or YAP1 shRNA (**f**). Arrows point to tensed cortical F-actin and stretched cell borders. Data represent mean ± SEM. One-way ANOVA with Tukey’s post hoc test. For YAP1^5SA^: WT, *n* = 54–80 cells; PB2 KO, *n* = 33–50 cells. **P = 0.0053 and ***P < 0.0001. For YAP1 shRNA: WT, *n* = 62–89 cells; PB2 OE, *n* = 100 cells each. *P = 0.012 and ***P < 0.0001. n.s. not significant. Data represent mean ± SEM. **g** Comparative Ingenuity pathway analysis (IPA) of enriched gene pathways for the unique DEGs identified in *PLXNB2* KO or OE hESCs relative to WT. Note the enhanced activation of ERK/MAPK and calcium signaling in Plexin-B2 OE cells. The categories are ranked by adjusted *P* value computed by IPA’s Fisher’s Exact Test. **h** WBs show increased ERK1/2 phosphorylation (p-ERK1/2) in *PLXNB2* OE cells compared to WT. p-ERK1/2 was further increased by DN RAP2A^N17^ expression in all three groups (WT, PB2 KO, and PB2 OE). In contrast, CA RAP2A^V12^ expression resulted in reduced p-ERK1/2 in *PLXNB2* OE cells. ERK1/2 served as a loading control. arb.units arbitrary units. One-way ANOVA followed by Dunnett’s post hoc correction to compare to WT group. A two-sided unpaired *t*-test was applied when comparing data from two groups (brackets). *n* = 3 samples per group. *P = 0.017 (WT vs. PB2 OE), *P = 0.028 (Control PB2 OE vs. CA RAP2A^V12^ PB2 OE), *P = 0.0024 (Control PB2 OE vs. DN RAP2A^N17^ PB2 OE), ***P < 0.0001. Data represent mean ± SEM. **i** Calcium imaging with Fluo-3-AM dye shows higher levels of intracellular calcium in hESCs at colony edges compared to central areas. Calcium signals were elevated in *PLXNB2* OE. Kruskal–Wallis test followed by Dunn’s multiple comparisons test. For colony edges, *n* = 328, 332, and 116 for WT, PB2 KO, and PB2 OE, respectively. For colony center, *n* = 1000, 648, 417 cells for WT, PB2 KO, and PB2 OE, respectively. ***P < 0.0001. Orange lines indicate median values and blue lines 25 and 75% percentiles.

Cells can respond to the mechanical properties of the surrounding physical environment, such as substrate rigidity, through the mechanosensitive transcription factors YAP/TAZ^36^, which are the main executors of the Hippo pathway^37^. Of note, our transcriptomic analysis had indicated an effect of Plexin-B2 manipulations on Hippo/YAP/TAZ signaling (see Fig. S2a). To explore whether Plexin-B2 affects YAP/TAZ activation in hESC colonies, we examined the nuclear localization of YAP/TAZ as a readout—i.e., inactive YAP is phosphorylated and retained in the cytoplasm for degradation, whereas active YAP is translocated into the nucleus to drive pro-growth gene programs^62,63^. IF revealed a nucleus-to-cytoplasm shift of YAP/TAZ in Plexin-B2-deficient hESCs as compared to WT, and conversely, a cytoplasm-to-nucleus shift in OE cells (Figs. 7a and S9a). It is worth noting that, unlike differentiated cells, human PSCs are insensitive to contact inhibition as they display a sustained basal YAP-driven transcriptional activity despite growing as dense colonies^64^.

Cytoskeletal mechanotension ensured by Plexin-B2 signaling may in fact facilitate response to extracellular mechanical cues such as substrate stiffness. To address this, we examined YAP activation in WT or Plexin-B2 mutant hESCs grown on soft or stiff substrates. Results showed that WT and Plexin-B2 OE hESCs responded to softer substrates with reduced nuclear YAP and to a stiffer substrate with YAP nuclear translocation, with Plexin-B2 OE cells responded more robustly than WT cells with higher levels of nuclear YAP (Fig. 7a). In contrast, Plexin-B2 KO hESCs were insensitive to substrates stiffness in terms of YAP nuclear localization, which remained low in all conditions.

Consistently, WB showed higher levels of YAP in *PLXNB2* OE hESCs than in WT (Fig. 7b). Furthermore, the expression of YAP target genes *CYR61, CTGF*, and *ANKRD1* was robustly upregulated in response to Dox-induced overexpression of Plexin-B2 in hESCs (Fig. 7c). Our RNA-seq data also showed transcriptional changes of YAP signature genes in Plexin-B2 mutant hESCs as compared to WT, and principal component analysis (PCA) based on the expression of YAP signature genes showed clear segregation of WT, *PLXNB2* KO, and OE hESC samples, confirming that each genotype leads to a distinct state of YAP signaling (Fig. S9b, c). Interestingly some of the YAP target genes responded differently to constitutive overexpression vs. acute upregulation of Plexin-B2 in the inducible model (Fig. S9b). This may be reconciled by the fact that certain YAP target genes have been shown to act as a transient response to biomechanical stimuli^65^.

Additionally, the introduction of CA RAP2A resulted in YAP inactivation (nucleus-to-cytoplasm shift) in all three genotypes, phenocopying Plexin-B2 KO, whereas DN RAP2A reverted YAP inactivation in Plexin-B2 KO cells (Fig. 7d).

To further interrogate a functional link between Plexin-B2 and YAP signaling, we introduced a Dox-inducible CA form of YAP (YAP1^5SA^) into WT or *PLXNB2* mutant hESCs (Fig. S9d), which led to angular cell morphologies and strengthened cortical F-actin in WT but also in *PLXNB2* KO hESCs, reminiscent of the OE phenotypes (Fig. 7e). Conversely, *YAP1* KD by shRNA resulted in diminished cortical F-actin in WT hESCs, but also attenuated the hypercontractile phenotype of Plexin-B2 OE cells (Figs. 7f and S9e, f).

As another way to test the functional relationship of Plexin-B2 and YAP and *β*-catenin in hESCs, we exposed hESCs to verteporfin (VP), an inhibitor of YAP signaling, or cardamonin, which promotes degradation of β-catenin (Fig. S10a). Results showed that VP markedly reduced proliferation of WT and *PLXNB2* OE hESCs, but did not further diminish the proliferation of KO cells (Fig. S10b), thus supporting YAP as a downstream effector of Plexin-B2 in regard to hESC proliferation. Correspondingly, the colony size was reduced in WT and Plexin-B2 OE by VP, but VP did not further reduce the size of *PLXNB2* KO colonies, which were already smaller than WT (Fig. S10b). VP also phenocopied *YAP1* KD in reducing cortical F-actin in WT or Plexin-B2 OE hESCs, and attenuated the hypercontractile appearance of OE cells (Fig. S10c). In contrast, cardamonin did not significantly alter the proliferation or cortical F-actin patterns in hESCs of either of the three genotypes, but it altered cellular arrangement in WT hESC colonies (Fig. S10b, c). These observations fit with our mathematical simulations that Plexin-B2 primarily controls actomyosin contractility, with secondary effects on adhesion complexes during the multicellular organization.

Hence, YAP can not only respond to Plexin-B2-mediated cytoskeletal tension but can mediate cytoskeletal dynamics and stem cell state in hESC colonies. Moreover, Plexin-B2-mediated cytoskeletal tension ensures mechanoresponse of YAP to extracellular mechanical cues.

### Altered biomechanics affects ERK1/2 activation, calcium distribution, and stem cell physiology in hESC colony

We next investigated the effect of Plexin-B2 on stem cell state. Comparative analysis of differentially engaged signaling pathways in *PLXNB2* KO or OE hESCs relative to WT indicated activation of ERK/MAPK and calcium signaling (Fig. 7g). We, therefore, assessed ERK1/2 activation by WB, which indeed showed elevated levels of phosphorylated ERK1/2 in *PLXNB2* OE hESCs relative to WT or KO cells (Figs. 7h and S10d). Again, the effect of Plexin-B2 OE on ERK1/2 activation could be reverted by expression of CA RAP1/2; and conversely, expression of DN RAP1/2 led to a marked increase of ERK1/2 activation in all three genotypes (Figs. 7h and S10d), further supporting RAP1/2 as a downstream effector of Plexin-B2 in this regard.

We next performed live calcium imaging of hESC colonies, which revealed a distinct pattern of cytosolic calcium levels—higher at colony periphery and lower in colony interior—in agreement with the higher traction forces at colony edges^6^ (Fig. 7i). Notably, *PLXNB2* KO and OE disrupted the spatial pattern of calcium within the colonies, echoing the disarray of actomyosin networks. Furthermore, calcium signals were in general elevated in *PLXNB2* OE cells (Fig. 7i).

Interestingly, the altered biomechanical properties of hESC colonies from Plexin-B2 manipulations also affected the orientation of mitotic spindles at the colony edge. In WT and *PLXNB2* OE colonies, the cell division planes at the colony periphery were largely oriented perpendicular to the colony rim, in alignment with in-plane traction forces, but they were randomly oriented at the colony center (Fig. S11). In contrast, cell division planes in KO colonies were largely randomized in all areas (Fig. S11). Hence, distinct mechanical niches as orchestrated by Plexin-B2 govern mitotic spindle orientation in hESC colonies.

**Fig. 8:**
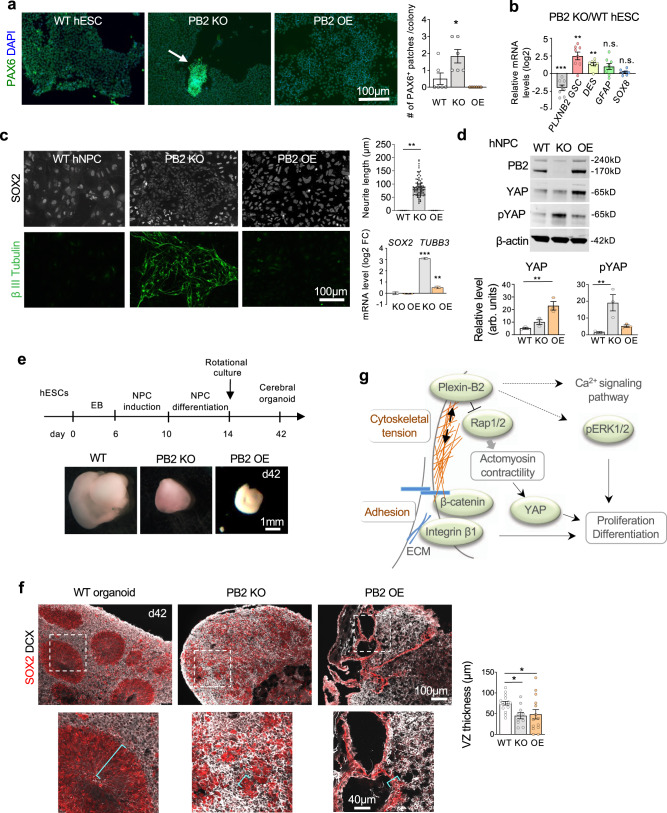
Plexin-B2 affects stem cell differentiation and neuroepithelial architecture. **a** IF images and quantification show small patches of PAX6^+^ cells in Plexin-B2 deficient hESC colonies (arrow). One-way ANOVA followed by Tukey’s post hoc test. *n* = 6 colonies per group. *P = 0.019. Data represent mean ± SEM. **b** qRT-PCR results confirm induction of marker genes for differentiation in Plexin-B2 deficient hESCs relative to WT. Note the verification of reduced *PLXNB2* expression in KO cells. Two-sided unpaired *t*-test. *n* = 8 samples per group. **P = 0.0036 (for *GSC*), **P = 0.0015 (*DES*), and ***P = 0.0002 (*PLXNB2*). n.s. not significant. Data represent mean ± SEM. **c** IF images show increased expression of β-III tubulin at the expense of SOX2 in *PLXNB2* KO hNPCs derived from hESCs (clone KO#2). Quantifications show enhanced neurite length (visualized by β-III tubulin IF) in *PLXNB2* KO cells as compared to WT or *PLXNB2* OE cells. One-way ANOVA followed by Tukey’s multiple comparisons test versus WT. *n* = 4 random fields per group. **P = 0.0014. Right, qRT-PCR results show relative transcript levels of *SOX2* and *TUBB3* in *PLXNB2* KO and OE hNPCs relative to WT. One-way ANOVA followed by Tukey’s multiple comparisons test versus WT. *n* = 3 samples per group. **P = 0.0048; ***P < 0.0001. Data represent mean ± SEM. **d** WBs show levels of total YAP and pYAP in hNPCs in dependence of Plexin-B2. β-actin as a loading control. arb.units arbitrary units. One-way ANOVA followed by Dunnett’s multiple comparisons test. *n* = 3 samples per group. For YAP, **P = 0.0026. For pYAP, **P = 0.0085. Data represent mean ± SEM. **e** Timeline of cerebral organoid derivation from hESCs. Images show smaller size of cerebral organoids with Plexin-B2 perturbation relative to WT at matching time points (day 42). **f** IHC images show SOX2^+^ neuroprogenitors and DCX^+^ neuroblasts residing in ventricular structures in WT cerebral organoids, but in disarray in mutants. Magnified images are shown below, with brackets marking ventricular zone (VZ) thickness, as quantified in bar graphs on the right. One-way ANOVA followed by Tukey’s multiple comparisons test versus WT. *n* = 17, 10, 14 ventricular zones for WT, KO, and OE, respectively. *P = 0.042 (WT vs. KO) and *P = 0.047 (WT vs. OE). Data represent mean ± SEM. **g** Model of the link of Plexin-B2 signaling with mechanoregulation and stem cell functions.

Colony integrity is an indicator of the stemness state of hESCs^8^. Although hESCs continued to express the pluripotency factors SOX2, OCT4, and NANOG in *PLXNB2* KO or OE colonies (Fig. S12a), we found that Plexin-B2-deficient colonies harbored small patches of cells expressing neural stem cell marker PAX6, indicating premature neural lineage differentiation (Figs. 8a and S12b). By comparison, Plexin-B2 OE did not cause premature induction of PAX6. Indeed, our RNA-seq data confirmed that *PLXNB2* KO hESCs upregulated genes related to ectoderm differentiation, dopamine neurogenesis, and neural crest differentiation, indicative of premature neuronal differentiation in KO colonies, while *PLXNB2* OE cells upregulated genes related to mesodermal commitment and cardiac progenitor differentiation (see Fig. S2a).

For corroboration of premature lineage commitment in *PLXNB2* KO hESCs, we carried out qRT-PCR array assays on the expression of a set of stem cell and differentiation markers. Around 90% of these markers exhibited no significant transcriptional alteration in *PLXNB2* KO relative to WT hESCs, including ESC markers *OCT4*, *NANOG*, and *SOX2* (Fig. S12c). Of the remaining 10% markers that showed transcriptional changes, notably all displayed upregulation in *PLXNB2* KO cells, including markers for the three germ layers: i.e., *GSC* (endoderm), *DES* (mesoderm), as well as neural markers, e.g., *GFAP* and *SOX8* (Fig. S12c), which were further confirmed by qRT-PCR (Fig. 8b).

### Plexin-B2 affects the proliferation and differentiation of hNPCs and neuroepithelial cytoarchitecture

We next studied the effect of Plexin-B2 on hNPC biology. We found that as in hESCs, *PLXNB2* KO also resulted in reduced proliferation of hNPCs, while *PLXNB2* OE did not further enhance the already high proliferative state of hNPCs (Fig. S13a).

Upon neural induction of hESCs, Plexin-B2 deficient cells consistently displayed accelerated neuronal differentiation compared to WT counterparts, as evidenced by enhanced induction of the neuroblast marker doublecortin (DCX) and the neuronal marker β-III tubulin, and reduced expression of NPC markers PAX6 and SOX2 (Figs. 8c and S13b, c). Analyses by qRT-PCR confirmed upregulation of *TUBB3* (encoding β-III tubulin) in *PLXNB2* KO cells, whereas *PLXNB2* OE did not cause accelerated neuronal differentiation (Fig. 8c).

In addition, WB also showed a response of YAP to Plexin-B2 manipulation in hNPCs, with higher pYAP levels (signifying inactivation) in *PLXNB2* KO cells, but elevated YAP levels in OE cells (Fig. 8d). We further tested the effect of β-catenin or YAP inhibitors on neural induction (Fig. S13d). For β-catenin inhibition with cardamonin, we observed no overt effects on neuronal differentiation in either of the three groups, with *PLXNB2* KO cells similarly displaying enhanced neuronal differentiation with or without cardamonin exposure. For YAP inhibition with VP, we observed that limited VP exposure indeed promoted neuronal differentiation for WT and *PLXNB2* OE cells, a phenotype similar to Plexin-B2 KO. These data provide additional support for a link of Plexin-B2 and YAP in controlling stem cell differentiation.

To further probe the role of Plexin-B2 in neuroepithelial tissue morphogenesis, we generated 3D cerebral organoids from hESCs using a forebrain organoid differentiation protocol^66,67^. After 6 weeks of culture, layered cortical structures emerged in cerebral organoids, with ventricular zones occupied by neuroprogenitors (SOX2^+^) and cortical plate-like zones occupied by differentiating neuroblasts (DCX^+^) (Fig. 8e, f).

Cerebral organoids derived from *PLXNB2* KO or OE hESCs were consistently smaller than WT organoids (Fig. 8e). Histological analysis of organoid cross sections revealed severe disruption of neuroepithelial cytoarchitecture in mutant organoids, e.g., absence of organized ventricular structures, which were replaced with numerous simple cysts in *PLXNB2* KO organoids, and large cystic structures with stretched and irregular contours in OE organoids (Fig. 8f). Notably, both SOX2^+^ and DCX^+^ cells were present in mutant organoids, albeit in spatial disarray (Fig. 8f). The disorganization of neuroepithelium was also reflected by the disarray of the actomyosin network, activated integrin β1, pFAK, as well as junctional N-cadherin, corresponding to Plexin-B2 manipulations (Fig. S13e). Thus, Plexin-B2 is not strictly required for neural induction per se, but affects cellular alignment and architectural integrity during neuroepithelial morphogenesis.

## Discussion

The multicellular organization relies on force-mediated processes that orchestrate cytoskeletal organization across cells in order to keep individual cells in a certain shape while maintaining tissue cohesion and structural integrity^68^. The signaling pathways that transmit external guidance cues into cytoskeletal tension and collective formation of actomyosin network and adhesive complexes need to be better mapped.

Here, we provided multiple lines of evidence to support the role of the guidance receptor Plexin-B2 in orchestrating actomyosin cytoskeletal network and cell–cell adhesion during multicellular development of hESCs and hNPCs. This impacts signaling pathways of β-catenin, YAP, ERK1/2, and calcium, all critical for stem cell physiology (Fig. 8g). From a mechanical perspective, the correlation between the experimental AFM data and mathematical simulations indicated that Plexin-B2 primarily promotes actomyosin contractility, which in turn affects tensile forces exerted on cell–cell junctions and cell–matrix adhesion sites, underscoring the interconnectedness of these mechanical elements^8,69,70^. The fundamental mechanoregulatory function of Plexin-B2 is consistent with an early evolutionary appearance of semaphorins and plexins at the transition from unicellular to multicellular organisms to adjust cytoskeletal tension and adhesion strength^29^.

From a signaling perspective, we found that the extracellular and the Ras-GAP domains of Plexin-B2 are indispensable to rescue the KO phenotypes, in agreement with a high degree of evolutionary conservation of these domains^29^. Plexin-B2 can be activated by class 4 semaphorins^71^; however, whether the mechanoregulatory function of Plexin-B2 requires semaphorins need further studies. In this context, it is notable that a semaphorin-independent mechanosensory function of Plexin-D1, a paralog of Plexin-B2, has recently been reported in endothelial cells, and this involves the bending of its extracellular domain in response to blood flow shear stress^72^. Whether this applies to Plexin-B2 in regulating multicellular organization awaits future studies, but it is worth noting that the current study and the earlier study^72^ converge on the mechanical effects of Plexins in inducing cytoskeletal changes, calcium surge, and ERK1/2 activation.

The effects of Plexin-B2 on stem cell physiology may be linked to YAP and β-catenin signaling. We demonstrated that YAP can respond to Plexin-B2-mediated cytoskeletal tension, and can itself mediate cytoskeletal dynamics. Consistently, YAP mutation in fish models results in flattening of the body, indicating a requirement of YAP for tissue integrity^47^. Our study also established Rap1/2 as downstream effectors of Plexin-B2 in orchestrating actomyosin network, YAP, and ERK1/2 activation, as well as stem cell proliferation in hESC colonies. Intriguingly, Rap2A has been shown as a key intracellular signal transducer that relays ECM rigidity to YAP/TAZ^7^. This raises a tantalizing possibility that Rap1/2 may relay mechanical information from Plexin-B2 to YAP.

Interestingly, Worzfeld and colleagues have recently implicated Plexin-B1 and -B2 in mechanosensing during epidermal development to detect compressive forces, such as those elicited by cell confluency, resulting in YAP inactivation^18^. This study and ours converge on the roles of Plexins in enhancing the stiffness of cortical actin cytoskeleton, stabilizing cell–cell adhesive complexes, and conferring mechanosensitivity. However, perhaps reflecting tissue-specific roles, the two studies diverge on the way Plexin-B2 controls YAP activation and thereby cell proliferation and contact inhibition: while in hESCs, Plexin-B2 deletion resulted in decreased nuclear YAP and reduced proliferation, in epidermal stem cells, loss of Plexin-B1 and -B2 led to increased nuclear YAP and over-proliferation^18^. Two observations may explain these divergent functions: First, differentiation states of epidermis and hESCs are different; second, whereas epidermis responds to biomechanical constraints from compressive forces in confluent cell layers, human PSCs are insensitive to such contact inhibition^64^.

From a developmental point of view, our data solidify the principle that mechanical forces and tissue architecture provide an overarching signal to inform cell fate decisions^73^. Our data showed that Plexin-B2 deletion reduces cell-intrinsic stiffness of hESCs and hNPCs, and this is associated with neuronal cell fate commitment. This echoes a previous report that soft matrix substrate promotes neural differentiation of hESCs, while stiff substrate does not affect the pluripotency state^74^. Interestingly, in mesenchymal stem cells, the lineage specification is also sensitive to tissue elasticity, with softer matrix promoting neuronal while stiffer matrix promoting muscle differentiation^75^. Hence, cytoskeletal tension as controlled by Plexin-B2 exerts similar effects on cell fate commitment as substrate stiffness. Our study may thus point to strategies to accelerate neuronal differentiation by manipulating intrinsic cell mechanics.

The mechanoregulatory function of Plexin-B2 is more critical for structurally complex tissues such as ventricular zones and neuroepithelium. In cerebral organoids, Plexin-B2 perturbation caused ventricular malformation, reminiscent of the neural tube closure defect seen in *Plxnb2* KO mice^12^, and echoing earlier reports that neural tube closure defect can arise from dysregulated mechanical interactions and tissue tension, thereby impacting cellular alignment and NPC function^10^. Our study also has implications for development and cancer. Indeed, reduced Plexin-B2 activity has been suspected to contribute to the neurodevelopmental phenotypes of Phelan-McDermid syndrome (22q13 deletion)^76^. In breast cancer and glioblastoma, Plexin-B2 is upregulated and promotes tumor growth and invasion^77,78^. Resonating with our current study, we have also recently demonstrated that Plexin-B2 provides the biomechanical dynamics required for invasive growth of glioblastoma cells^17^.

In sum, our study identified a critical role of Plexin-B2 in organizing the actomyosin network and cytoskeletal tension during the multicellular organization of hESCs and hNPCs. Our data underscore the importance of force-mediated processes in controlling proliferation, cell fate specification, and tissue morphogenesis. Our findings may have broad implications for understanding neurodevelopmental disorders, tissue repair, and cancer biology.

## Methods

### hESC culture

All culture experiments were conducted using the WA09 (H9) hESC^79^ line obtained from the University of Wisconsin and validated at the Mount Sinai Stem Cell core facility. The Mount Sinai Stem Cell core conducts routine quality controls by verifying karyotype and mycoplasma-free status for all stem cell lines. Briefly, H9 hESC were seeded on matrigel-coated cell culture dishes (1:100, Corning) and grown in mTeSR1 medium (STEMCELL Technologies). Cells were passaged around every 5 days by dissociation with Accutase (BD Biosciences) and addition of 2.5 μM Rho-associated protein kinase (ROCK) inhibitor Thiazovivin (Millipore). The experiments were conducted at early hESC passages (P1 or P2) upon viral infections. The study was approved by the Embryonic Stem Cell Research Oversight Committee (ESCRO) at Icahn School of Medicine at Mount Sinai.

### CRISPR genome editing for Plexin-B2 knockout

The CRISPR/Cas9-sgRNA sequence targeting *PLXNB2* exon 3 was embedded inside a CRISPR/Cas9 lentivirus plasmid^80,81^ to generate plentiCRISPRv2-sgPLXNB2 (target sequence: GTTCTCGGCGGCGACCGTCA). Lentiviral particles were produced in HEK293T cells (Takara, no. 632180) by co-transfection of lentiviral plasmid, envelope plasmid pMD2.G, and packaging plasmid psPAX2 (Addgene plasmids #12259 and #12260; deposited by Didier Trono, EPFL Lausanne). Low passage hESCs transduced with the lentivirus were selected with puromycin (1 µg/ml) for 7 days. Successful knockout of Plexin-B2 was confirmed by WB, demonstrating the absence of mature Plexin-B2 (170 kDa). A lentiviral vector (plentiCRISPRv2-sgEGFP) expressing sgRNA targeting eGFP (target sequence: GGGCGAGGAGCTGTTCACCG) was used as control (WT).

Plasmids cloned for this study have been deposited at Addgene (“Roland Friedel Lab Plasmids”; https://www.addgene.org/Roland_Friedel/).

### CRISPR clones sequencing and karyotyping

To isolate clonal lines of hESCs that carry defined CRISPR mutations, after cells were transduced with lentiviruses (either targeting Plexin-B2 or EGFP as control) and selected with puromycin as described above, three colonies were picked, expanded, and sequenced for verifying mutations at the *PLXNB2* locus. Three PB2 KO and its respective WT clones were confirmed. Normal karyotypes of selected clonal lines were confirmed by chromosome G-banding at Mount Sinai Genetic Testing Laboratory Sema4 (New York, USA).

### Lentiviral vectors for Plexin-B2 rescue, overexpression, and signaling mutants

For rescue experiments, human *PLXNB2* cDNA (entry clone HsCD00399262, DNA Resource Core at Harvard Medical School) was modified by site-directed mutagenesis to generate a CRISPR-resistant *PLXNB2* cDNA, which was inserted into the lentiviral vector pLenti-PGK (plasmid backbone pLENTI-PGK Neo DEST, Addgene #19067; deposited by Eric Campeau & Paul Kaufman) by Gateway LR Clonase II (Thermo Fischer Scientific). The resulting vector pLV-*PLXNB2* was then used to generate lentiviruses to transduce *PLXNB2* KO hESCs for rescue/structural mutants’ experiments. The pLV-*PLXNB2* without CRISPR-resistant mutation was used to generate lentivirus for overexpression experiments by transducing H9 hESCs.

The mutations in functional domains of Plexin-B2 were generated in the CRISPR-resistant *PLXNB2* cDNA plasmid using the Phusion Site-Directed Mutagenesis Kit (Thermo Fischer): mGAP (mutation in the GAP domain: R1391A, R1392G), mRBD (mutation in the RBD domain: LSK 1558–1560 -> GGA), ∆VTDL (deletion of four last C-terminal amino acids VTDL of PDZ binding motif), and ∆ECTO (lacking extracellular domain, deletion of amino acids 30-1189). The mutant cDNAs were transferred into pLenti-PGK destination vector by Gateway cloning as described above.

### Doxycycline-inducible Plexin-B2 overexpression and shRNA KD

A plasmid for a lentiviral vector for doxycycline (Dox)-inducible human Plexin-B2 overexpression was generated by inserting human *PLXNB2* cDNA by Gateway cloning into pLenti-CMVtight-Puro DEST (Addgene #26430). Target cells were coinfected with a lentivirus expressing Tet-On 3 G transactivator protein and hygromycin resistance (VectorBuilder, VB160122 -10094). Stable *PLXNB2* Tet-On hESC lines were established by puromycin (1 μg/ml) and hygromycin (200 μg/ml) selection of coinfected cells.

Temporally controlled KD of Plexin-B2 at different phases of hESC colony expansion was achieved with TET-ON lentiviral vectors (Tet-pLKO-Puro backbone, Addgene #21915, deposited by Dimitri Wiederschain) expressing Dox-inducible shRNA targeting *PLXNB2* and puromycin resistance marker (pLKO-Tet-On-*PLXNB2*–shRNA1 and –shRNA2). A non-targeting shRNA vector (pLKO-Tet-On-shRNA-Control) served as control (oligonucleotides used for construction of shRNA vectors are listed in file Supplementary Data 1). Stable PLXNB2 shRNA lines were established by puromycin selection (1 μg/ml) over 7 days. The *PLXNB2* overexpression and the shRNA-mediated *PLXNB2* KD were induced by adding 1 μg/ml Dox (MP Biomedicals) to the culture medium.

### Doxycycline-inducible YAP overexpression and shRNA KD constructs

DNA plasmids for lentiviral vectors with doxycycline-inducible human *YAP1* and *YAP1-5SA* cDNAs were generated by VectorBuilder, Inc.. Plasmids were designed using VectorBuilder website tools. In brief, human *YAP1* plasmid was designed using an mRNA sequence from NCBI transcript NM_001282101, and human *YAP1-5SA* was designed based on ref. ^82^ to generate an unphosphorylated CA form of *YAP1*. Both WT and mutant *YAP1* plasmids are driven by a TRE3G promoter and have a CMV-puromycin-T2A-mCherry cassette inserted after the gene of interest to facilitate the selection of transfected cells. Target cells were coinfected with a lentivirus expressing Tet-On 3G transactivator protein and hygromycin resistance (VectorBuilder, VB160122 -10094).

YAP short hairpin (sh)RNA lentiviral expression vectors were cloned by ligating oligonucleotides encoding the shRNA hairpins (Integrated DNA Technologies) into the pLKO-Tet-On vector (Addgene #21915) to have temporal control of YAP1 KD (oligonucleotide sequences are listed in file Supplementary Data 1). All plasmids were validated using restriction enzymes and Sanger sequencing (Macrogen) using the primer pLKO-shRNAseq (ggcagggatattcaccattatcgtttcaga).

### Lentiviral constructs for RAP1/2 small GTPases

To validate the relevance of the Ras family of small GTPases, human RAP2A cDNA was modified to generate a CA (RAP2A-V12) and DN (RAP2A-N17) isoforms using site-directed mutagenesis (Thermo Fischer). The RAP2A isoforms were then inserted into the lentiviral vector pLenti-PGK Neo or Puro DEST by Gateway LR Clonase II (Thermo Fischer). For RAP1B, CA (bovine Flag-RAP1B-V12, Addgene #118323) and DN (bovine Flag-Rap1B-N17, Addgene #118322) were inserted into the same lentiviral vectors as described above for RAP2A. The resulting vectors were used to transduce WT, PB2 KO, and PB2 OE hESCs.

### hESC colony and 3D aggregation assays

For adherent hESCs culture, cells were plated at a density of 2.5 × 10^4^ in Matrigel-coated six-well plates. Individual colonies were imaged from day 1 to day 6 with a CKX53 inverted microscope (Olympus) using the Olympus cellSens Entry software, and colony sizes were measured using ImageJ.

A 3D aggregation assay was performed by culturing 4 × 10^4^ hESCs resuspended in mTeSR1 medium in low-adhesion 24-well plates with the addition of 10 μM Thiazovivin. The size of hESC aggregates was evaluated after 48 h. For the hanging drop assay, 2.5 × 10^4^ hESCs were resuspended in 10 µl of mTeSR1 medium with 10 μM Thiazovivin for 24 h. Images were taken using a Nikon SMZ745D microscope, and areas of clustered cells were measured using ImageJ.

### Culture of hESCs on hydrogels with different stiffness

To investigate the effects of substrate stiffness on YAP localization in hESCs carrying Plexin-B2 mutations, we used synthetic peptide hydrogels (Manchester Biogel) of different stiffness: PeptiGel-alpha 4 (~1.0 kPa) and PeptiGel-gamma 2 (~5.0 kPa). Gel solution was centrifuged to remove air bubbles and 100 μl each was distributed into chambers of a four-chamber 35 mm glass-bottom dish (CellVis). The chambers containing hydrogel solutions were overlayed with DMEM/F12 media containing Matrigel (1:100, Corning) and incubated for 1 h at 37 °C to induce gelling. Media was carefully removed and hESCs were seeded on the top of the gels at a density of 10^4^ cells per chamber in mTeSR1 media containing 2.5 μM Thiazovivin (Millipore). As control condition, hESCs were plated into Matrigel-coated glass coverslips only. The media was replaced daily and the cells were maintained for 3 days before fixation and imaging.

### Calcium imaging of hESCs

For calcium imaging of hESCs, 4 × 10^4^ cells were seeded in a Matrigel-coated four-chamber 35 mm glass-bottom dish (CellVis) and grown for 3 days. The cells were loaded in mTeSR1 medium supplemented with 10 µM Fluo-3 AM (Biotium; diluted in 20% Pluronic F-127) for 30 min at 37 °C, washed, and superfused with fresh mTeSR1 medium. Fluo-3 AM was excited at 488 nm and emitted fluorescence above 505 nm was recorded by an LSM 780 confocal microscope (Zeiss) in frame scan mode on a heated stage. Hoechst 33342 (1:2,000, Thermo) and CellMask deep red (1:2,000, Thermo) dyes were used for nuclear and plasma membrane staining, respectively.

### Neural differentiation

Human neural progenitor cells (hNPCs) were generated by a single-cell monolayer protocol using STEMDiff Neural Induction Medium (StemCell Technologies). For the monolayer protocol, hESCs were plated at a density of 2.5 × 10^5^ cells/cm^2^ in STEMDiff medium, and on day 6 the cells were replated at the high confluence. After day 11 the cells were plated on glass coverslips coated with Matrigel and analyzed by immunocytochemistry (ICC) to confirm the NPCs identity and used for subsequent experiments. Neurite length was measured using the ImageJ plugin Simple Neurite Tracer.

### Cerebral organoid derivation from hESCs

Cerebral organoids were generated from hESCs by forming the first embryoid bodies that were subsequently transferred to Matrigel droplets for maturation into organoids^66,67^. Briefly, H9 hESCs were dissociated into single cells with Accutase (BD Biosciences) and plated in ultra-low-attachment 96-U-well plates (Costar) at a density of 9000 cells/well (150 μl) in mTeSR1 medium (STEMCELL Technologies) containing 10 μM Thiazovivin (Millipore). After 6 days, embryoid bodies were cultured in STEMDiff Neural Induction Medium (STEMCELL Technologies). The neuroepithelial tissue that formed over the next 4–6 days was transferred into 30 μl Matrigel (Corning) droplets and grown for 4 days in stationary culture in a medium composed of DMEM-F12 (Invitrogen), Neurobasal medium (Invitrogen), N2 supplement (Invitrogen), human insulin (Sigma), GlutaMAX supplement (Gibco), MEM-NEAA (Gibco), penicillin-streptomycin (Sigma), 2-mercaptoethanol (Millipore), and B27 supplement minus vitamin A (Invitrogen). This was then followed by culturing the droplets in six-well plates on an orbital shaker with the addition of a B27 supplement plus vitamin A (Invitrogen) to the media to promote neuronal differentiation. At day 42 cerebral organoids developed to a size of ~3–5 mm in diameter and were analyzed.

### Immunocytochemistry and immunofluorescence

For ICC, cells were fixed in 4% formaldehyde in PBS at room temperature for 10 min, washed 3x with PBS, permeabilized and blocked with 5% donkey serum, and 0.3% Triton X-100 in PBS for 1 h. Primary antibodies were incubated at 4 °C overnight in dilution buffer composed of PBS with 1% BSA and 0.3% Triton X-100. Secondary antibodies were incubated in dilution buffer at room temperature for 1 h together with nuclear staining (DAPI, 1:1000; Thermo Fisher). For most preparations, the cells were also counterstained for F-actin with Alexa Fluor 568 Phalloidin (Thermo Fisher).

For histological analysis of cerebral organoids by IF, cerebral organoids were fixed in 4% paraformaldehyde/PBS at 4 °C for 15 min, washed with PBS, cryoprotected in 30% sucrose at 4 °C overnight, embedded in OCT (Fisher HealthCare), and frozen on dry ice. The organoids were sectioned using a cryostat (Leica) at 12 μm thickness and the cryosections were used for antibody staining following the protocol described above for ICC.

### Western blot

For WB, freshly collected samples were lysed in RIPA buffer (Sigma) containing protease (Fisher Scientific) and phosphatase inhibitors (Sigma). Protein concentrations were determined using a BCA assay (Thermo Scientific). Proteins were resolved on 4–12% polyacrylamide NuPAGE gels (Invitrogen) using the XCell SureLock system (Invitrogen) and transferred to nitrocellulose membranes (Li-Cor Biosciences). The immunoreactive bands were detected by fluorescent ODYSSEY infrared imaging system (Li-Cor Biosciences). Equal protein loading was controlled by probing the blots with an antibody against a housekeeping control protein. Uncropped WBs can be found in the Source data file.

### Antibodies

The following primary antibodies were used for ICC/IF: anti-sheep plexin-b2 (ecd) (1:300, R&D systems, AF5329), anti-rabbit plexin-b2 (icd) (1:200, Abcam, ab193355), anti-rabbit sox2 (1:200, Abcam, ab97959), anti-guinea pig dcx (1:500, EMD Millipore, ab2253), anti-rabbit pmlc2 (1:200, Cell Signaling, 3671), anti-rat e-cadherin (1:200, Life Technologies, 131900), anti-mouse n-cadherin (1:200, BD Bioscience, 610920), anti-rabbit zo-1 (1:200, Thermo, 61-7300), anti-mouse integrin β1 (active, huts-4) (1:200, EMD Millipore, MAB2079Z), anti-mouse, oct4 (1:500, Abcam, ab184665), anti-rabbit pFak (1:200, Thermo, 44-624 G), anti-rabbit nanog (1:200, Abcam, ab109250), anti-rabbit caspase 3 (1:500, R&D systems, AF835), anti-mouse yap (1:200, Santa Cruz, sc-101199), anti-mouse β-catenin (1:200, BD Bioscience, 610153), anti-mouse pax6 (1:200, Abcam, ab78545), anti-βIII tubulin (tuj1) (1:100, R&D systems, MAB1195), anti-mouse paxillin (1:200, Invitrogen, AHO0492), anti-mouse p-vimentin (phospho S55) (1:200, Abcam, ab22651), anti-rabbit FN (1:200, EMD Millipore, ab2033).

The following secondary antibodies were used for ICC/IF: Alexa Fluor 488, 594, or 647-conjugated donkey anti-goat, -rabbit, -rat, or -mouse IgG, and anti-guinea pig IgG (Jackson ImmunoResearch Laboratories, 1:300).

For function-blocking assay: anti-mouse integrin β1 (P5D2) (10 μg/ml, Santa Cruz, sc-13590) and ChromPure Mouse IgG, whole molecule (1:1,000; Jackson ImmunoResearch Laboratories, 015-000-003).

The following primary antibodies were used for WB: anti-rabbit β-actin (1:10,000, Sigma, A1978), anti-sheep plexin-b2 (ecd) (1:500, R&D Systems, AF5329), anti-rabbit β1-integrin (4706 s) (1:1000, Cell Signaling, 4706), anti-mouse yap (1:500, Santa Cruz, sc-101199), anti-rabbit phospho-yap (Ser127) (1:1000, Cell Signaling, 4911 S), anti-rabbit phospho-p44/42 mapk (erk1/2) (thr202/tyr204) (1:1000, Cell Signaling, 9101 S), anti-mouse p44/42 mapk (erk1/2) (3a7) (1:1000, Cell Signaling, 9107 S), anti-mouse β-catenin (1:1000, BD Bioscience, 610153), anti-rabbit sox2 (1:10,000, Abcam, ab97959), anti-rabbit pax6 (1:2000, BioLegend, 901302), anti-rabbit gapdh (1:1000, Cell Signaling, 2118), anti-rabbit h3k4me2 (0.5 µg/ml, Active Motif, 39914). The following secondary antibodies were used: IRDye 800CW donkey anti-mouse (1:10,000, Li-Cor Biosciences, 926-32212), 680RD-donkey anti-goat (1:10,000, Li-Cor Biosciences, 926-68024), and anti-rabbit (1:10,000, Li-Cor Biosciences, 926-68073).

### Staining of cells for cytoskeleton and membrane components

To visualize F-actin in fixed cells, staining with fluorescently labeled phalloidin was performed (Alexa Fluor 568 phalloidin (1:100, Invitrogen, A12380), Alexa Fluor 647 phalloidin (1:500, Invitrogen, A22287). F-actin and pMLC2 foci were measured with the assistance of the ImageJ plugin BioVoxxel Toolbox Cluster Indicator. β-catenin peaks were measured using the ImageJ plugin BAR Finding Peaks.

To observe F-actin in live cells with the LifeAct-mScarlet sensor protein, hESCs and hNPCs were infected with lentiviral preparations expressing LifeAct-mScarlet^44^. The cells were selected with G418 (200 μg/ml) over 7 days and expanded for subsequent experiments. For staining with the fluorescent SPY555-Actin probe (Cytoskeleton, Inc), live hESCs were incubated with the dye for 1 h and imaged using the LSM 780 confocal microscope on a heated stage at 37 °C and 5% CO_2_ for probing the actin dynamics.

For CellMask (Thermo) plasma membrane staining, live hESCs were incubated with dye for 5–10 min and imaged immediately to record the dynamic changes of plasma membranes and cell borders. Alternatively, cell membranes were visualized in live cells with MemGlow 488 dye (1:200, Cytoskeleton, MG-01). Cell nuclei were stained with Hoechst 33342 dye (Thermo) or NucSpot Live 650 (Biotium). Based on the live staining described above, the circularity index of cells was calculated with the formula: 4π x area/perimeter^2. A value of 1.0 indicates a perfect circle; a value approaching 0.0 indicates an increasingly elongated shape.

### Total internal reflection microscopy (TIRF) and focal adhesion segmentation

TIRF microscopy was performed using a Leica DMi8 Infinity TIRF microscope and LASX (v3.7.0.20979). Focal adhesion morphometrics were assessed using IF staining of paxillin (at an imaging depth of 90 nm), which was imaged under PBS supplemented with ProLong Live antifade agent (Thermo P36975) Nuclear and actin channels were obtained simultaneously by epifluorescence using IF staining of phalloidin and DAPI as mentioned above. Images were captured using a 1.4 NA Leica 63 X oil TIRF objective at room temperature.

For quantification of focal adhesion morphometrics, binary masks of the FAs were generated in Fiji^83^. First, the background of the paxillin image had its background subtracted using the rolling ball method with *r* = 30. Next, the image was auto-thresholded using the moments method on a dark background. This was then converted to a binary mask and the “Fill Holes” operation was applied. Following this, the actin channel was used to generate a cell mask in the following manner: the actin channel was auto-thresholded using the mean method with a dark background. This was then converted to a binary mask with the “Dilate”, “Fill Holes”, and “Open” operations performed on it. The final mask used for quantification was the result of applying the logical AND operation on both masks. Segmented images of the nuclei were obtained using Cellpose on the DAPI channel. After this, the binary images were analyzed using an in-house Matlab script. To calculate the number of FAs per cell, the number of segmented FAs was divided by the number of segmented nuclei for each region imaged.

### High-content image analysis of cell morphologies

hESCs were seeded on a four-chamber 35 mm glass-bottom dish (CellVis) at a density of 2 × 10^4^ cells/well and cultured at 37 °C for 3–5 days. hNPCs were seeded at 4 × 10^4^ cells on glass coverslips 25 mm, #1½ (Electron Microscopy Sciences) and cultured at 37 °C for 2 days. The cells were fixed in 4% formaldehyde in PBS at room temperature and ICC was carried out as described in the respective section above. Cells were then incubated with primary antibodies in dilution buffer at 4 °C overnight and incubated with secondary antibodies (Alexa Fluor 488 and 647; Jackson ImmunoResearch) at 1:300 dilution in dilution buffer for 1 h at room temperature. Subsequently, the cells were counterstained for F-actin and nucleus with Phalloidin 568 (Thermo Fisher) and DAPI, respectively. Image acquisition (30 fields per condition) was carried out on LSM 710 confocal microscope (Zeiss) using 40X objective and zoom 0.6.

Cell segmentation was performed using Cellpose^84^. Briefly, nuclear objects were identified using the DAPI channel, and the corresponding cell objects were identified using the innate propagation algorithm of Cellpose coupled with the contrast-enhanced phalloidin or β-Catenin channel to define cell boundaries. Image analysis and quantification were performed using Matlab where multi-parametric analysis was performed for the four channel images, including morphometric (size and shape) and intensity. Measurements were exported as xls files and GraphPad Prism was used to generate box plots.

To calculate cell–cell distances from confocal microscopy images, centroid distances were calculated from DAPI images. We calculated the distance between every nucleus in each image. The five nearest cells for each cell in the image were picked up for histogram plotting. The histogram was normalized using a Probability density estimate function. All centroid distance calculations and histogram plotting were performed with Matlab R2018a software (MathWorks).

### Time-lapse video microscopy

For SPY555-Actin time-lapse microscopy, hESCs were plated at a density of 2 × 10^4^ cells per well in a four-chamber 35 mm glass-bottom dish (Cellvis) and left growing for 18 h. Live-cell time-lapse video microscopy was carried out for 21 h with frames taken every 20 min, using LSM 780 confocal microscope (Zeiss), with environmental control chamber conditions set at 37 °C temperature and 5% CO_2_. The analysis was carried out using Imaris software (Oxford Instruments).

### EdU Click-iT assay

To assess the proliferative potential of hESCs, cells were plated at a density of 2 × 10^4^ in Matrigel-coated eight-chamber slides, and after 3 days of culture, 10 µM of the nucleotide analog EdU (Life Technologies) was added to the medium for 30 min. The cells were immediately fixed in 4% formaldehyde at room temperature for 10 min and washed 3x with PBS. EdU Click-iT assay was performed accordingly to manufacturer’s instruction (Thermo) followed by DAPI staining. The number of EdU^+^ cells was quantified using Photoshop CS5 (Adobe) and ImageJ.

### EdU Click-iT flow-cytometry assay

hESC and hNPCs were grown as 2D cultures on Matrigel-coated dishes and treated with 10 µM EdU (Life Technologies) for 20 and 90 min, respectively. The cells were harvested with Accutase, washed with PBS, pelleted, and fixed in Click-iT fixative at room temperature for 15 min, protected from light. The cells were washed with 1% BSA in PBS, pelleted, and resuspended in 1X Click-iT saponin-based permeabilization and wash reagent and click labeled with EdU Click-IT flow-cytometry assay, accordingly manufacturer’s instruction (Thermo). DAPI (Invitrogen) was added to cell suspensions at a concentration of 5 μg/ml to stain the nuclei. Cells were resuspended in FACS buffer (Hibernate-E low fluorescence, BrainBits) with 0.2% BSA and 20 μg/ml DNase (Worthington) and passed through a 70-μm mesh filter. Cell suspensions were analyzed by flow cytometry (BD LSRII) and cell cycle data was evaluated with FACSDiva (BD) and FlowJo software (FlowJo LLC).

### Subcellular fractionation

A subcellular protein fractionation kit (Cell Signaling) was used to separate cell lysates into cytoplasmic (Cyto), membrane (Mem), and nuclear (Nuc) fractions based on the use of detergents. All procedures were performed accordingly to the manufacturer’s protocol. The fractions were mixed with 3x SDS loading buffer plus DTT and analyzed by WB.

### RNA extraction, library preparation, and RNA-seq

Total RNA from WT, PB2 KO, and PB2 OE hESCs were isolated using an RNeasy Micro kit (Qiagen), following the recommended instructions for on-column DNase digestion. RNA quality and concentrations were measured using an Agilent Bioanalyzer (Agilent RNA 6000 Pico), and samples with RNA integrity number scores higher than 7 were used for the experiments. Poly(A) mRNA were isolated using NEBNext Poly(A) mRNA Magnetic Isolation Module (NEB E7490S) and cDNA libraries for Illumina next-generation sequencing were prepared with NEBNext Ultra Directional RNA Library Prep Kit (NEB E7760S). Three independent samples for each condition were barcoded using NEBNext Index primers (NEB, E7335S), pooled together as one sample, and ran on an Illumina platform HiSeq2500 with one-sided 75 base reads, with about 30 million reads per sample.

### Differential gene expression analysis

Raw RNA-seq reads were mapped to the hg38 genome using HISAT2^85^. Counts of reads mapping to genes were obtained using featureCounts software of Subread package against Ensembl v90 annotation^86^. Differential expression analysis was carried out using the DESeq2 package^87^, and PCA in the DESeq2 package was utilized to identify outliers. Unsupervised clustering of samples and of DEGs was performed by calculating for each gene the transcripts-per-million (TPM) value, filtering for genes with a mean expression value of >20 TPM, and selecting genes with a differential expression of at least twofold between any of the samples. The hierarchical clustering analysis was performed with the clustergram function using Matlab R2018a (MathWorks).

Gene ontology analysis of common and exclusive PB2 KO and PB2 OE DEGs was performed with the ENRICHR resource (http://amp.pharm.mssm.edu/Enrichr/; accessed 05/2020). The scales are ranked by “Combined Score” (log *p* value multiplied by the *z*-score of the deviation from the expected rank). The same gene lists were also analyzed by NetworkAnalyst 3.0 (https://www.networkanalyst.ca/) and used to visualize Reactome-based gene categories^88^. The most relevant pathways were selected to display interactive analytics of gene networks. Ingenuity pathway analysis (IPA) was performed to compare PB2 KO with PB2 OE DEG lists (https://www.qiagenbioinformatics.com/products/ingenuity-pathway-analysis/; accessed 07/2020).

### qRT-PCR array and validation

Cells were lysed using RLT buffer (Qiagen) plus 2-mercaptoethanol (Millipore), and lysates were stored at −80 °C until RNA extraction. Total RNA was isolated using the RNeasy Mini Kit (Qiagen). RNA was converted to cDNA using oligo(dT) primer, and quantitative PCR reactions were set up using the PerfeCTa SYBR Green FastMix ROX qPCR kit (QuantaBio). Quantitative RT-PCR array analysis was performed using a hESC-specific PCR array (PAHS-081Y; SABiosciences). Reactions were run on an ABI/Life Technologies 7900HT real-time PCR instrument. The statistical analysis and plotting were performed on the RT2 Profiler PCR Array Data Analysis Webportal (http://dataanalysis.qiagen.com/pcr/arrayanalysis.php).

Validation of qRT-PCR array and for *PLXNB2* and *YAP1* and its target genes (*CYR61*, *CTGF*, and *ANKRD1*) were performed with individual qRT-PCR reactions (primers sequences are listed in file Supplementary Data 1). Relative mRNA expression levels were determined by ΔΔCt method relative to *GAPDH* or *ACTB* housekeeping gene controls. qRT-PCR data are average from at least three replicates for each sample.

### Treatment with YAP inhibitor VP or β-catenin inhibitor cardamonin

hESCs were seeded at a density of 10^4^ cells in Matrigel-coated eight-chamber slides, and after 3 days of culture, 0.5 µM of YAP inhibitor VP (Sigma) was added to the media for further culture of 24 h. The β-catenin inhibitor cardamonin (2 µM; Tocris) was added after 2 days of culture for further culture of 48 h. Cells were fixed in 4% formaldehyde/PBS at room temperature for 10 min and washed 3x with PBS before immunostaining and imaging.

For hNPC induction, hESCs were seeded according to the neural differentiation protocol described above and the β-catenin inhibitor cardamonin (0.5 µM; Tocris) was added in the fresh media daily from day 1 to day 10. The YAP inhibitor VP (0.5 µM; Sigma) was added in two 24 h pulses (days 5 and 9) to reduce toxicity effects.

### Stimulating hESCs with Sema4C

For Sema4C coating of culture dishes, Sema4C-Fc (R&D Systems) at 2, 10, and 50 µg/ml was mixed with Matrigel, and dishes were incubated for 1 h at 37 °C. hESCs were seeded at a density of 4 × 10^4^ cells per well and cultured for 2 days. The colonies were fixed, stained for cytoskeleton markers, and imaged.

For treatment with Sema4C soluble in media, hESCs were seeded at a density of 10^4^ cells per well of the eight-chamber slide and cultured for 2 days before being treated by addition of 2 µg/ml Sema4C-Fc (R&D Systems) to culture media for further 2 days.

### Cytoskeletal drugs and integrin antibody blocking experiments

Cytoskeletal drugs and integrin antibody blocking experiments were carried out directly in live hESCs transfected with LifeAct_mScarlet or SPY555-Actin for Y-16 treatment. Drugs or vehicles were added to media, and cells were incubated for 3 h in a cell incubator. Drugs were used at the following concentrations: Rock Inhibitor Y-27632 (20 µM), blebbistatin (10 μM), Rho-GEF inhibitor Y16 (25 µM), and latrunculin (5 μM). Integrin β1 antibody (P5D2; sc-13590) 10 μg/mL or IgG antibody (used as control) were added to cells in suspension for 15 min before plating cells in Matrigel-coated coverslips. The mTeSR media was supplemented with 2.5 μM Thiazovivin (Millipore) and cells were incubated for 1 h before imaging. After incubation hESCs were treated with SPY555-Actin and CellMask dye (Thermo) and imaged immediately using LSM 710 confocal microscope (Zeiss).

### AFM elastography

AFM measurements were conducted using an Asylum MFP 3D-BIO AFM equipped with environmental thermal and vibrational control coupled with an Olympus IX-80 inverted spinning disk confocal microscope^43^. For the experiments, hESCs were plated on 60 mm Matrigel-coated plastic dishes at a density of 5 × 10^5^ for WT and *PLXNB2* OE groups and 7.5 × 10^5^ cells for the *PLXNB2* KO group (to compensate for slower colony expansion). hNPCs were plated at a density of 4.2 × 10^6^ for WT and *PLXNB2* OE groups and 6.3 × 10^6^ cells for *PLXNB2* KO group on 60 mm dishes. After 3 days in culture, the plates were secured to the AFM and maintained at 37 °C for the entire duration of the AFM experiment. A gold-coated silicon nitride probe with a triangular-shaped body, blunted pyramidal tip, and nominal spring constant of 0.1 N/m (Cat #: TR400PB, Asylum) was used for cell measurements and calibrated using the thermal noise method. The Hertzian model was used to fit our indentation data for its elastic modulus, where this model called for probe tip half-angle (9°), probe material (silicon nitride), and sample Poisson’s ratio (*ν* = 0.45). To measure the colony elastic modulus a 24 × 24 indentation array was conducted over a 64 µm^2^ region of the colony (avoiding colony edges). The AFM was set to conduct its indentation array at an indentation rate of 0.5 Hz and indent each time until the probe deflected 50 nm (about 6 nN of force), which would trigger its retraction. For hESCs, each condition was measured for six-seven different colonies across different dishes from independent preparations. For hNPCs, each condition was measured for three areas across different dishes. For each cell group, every indentation was aggregated into a pool of measurements which was then averaged to return a mean colony stiffness. This aggregation allowed for proper sampling power regarding biological variability and batch variation. Due to the phenotypic nature of hNPCs, NPC cultures had open/dish exposed areas, which translated into direct dish measurements during AFM indentations, a phenomenon called substrate effect. To avoid these unwanted influences, NPC modulus values larger than 20 kPa were filtered out (expected NPC stiffness ~5 kPa). Mean colony stiffness trends and statistical comparisons were done on both filtered and unfiltered NPC data and confirmed to be equivalent.

### Cell topology imaging via AFM

In addition to elasticity measurements, the atomic force microscope (AFM) can image colony topology via direct contact. We employed the AFM imagining technique called contact mode imaging whereby a feedback loop is set upon the probe as it is told to slide across, and track, the surface of the colony in a raster scanning manner. hESCs were prepared as above except that cells were fixed with formaldehyde for 15 min. This allowed preservation of colony structure and features throughout the duration of AFM imaging, as well as increase colony robustness during extended AFM probe interaction. This step was acceptable for contact mode imaging but would not be appropriate for elasticity measurements as fixing may change the cells' elastic properties. The same type of probe used for AFM elasticity measurements was selected for contact mode imaging (see methods above), its 0.1 N/m spring constant and 42 nm tip radius allowed for gentler contact imaging and detection of finer cell features. A 90 µm^2^ region central to the cell colony was selected for imaging. The probe was prescribed to move at a speed of 200 μm/second and scan its 90 µm^2^ region in 256 segments, these 256 segments were then stitched together for a complete topography map. Scans used a setpoint = 0.8 V. Topology images revealed ruffles across the cell membrane particularly for the *PLXNB2* KO group. To quantify these ruffles a profile was taken through a selected cell, perpendicular to the ruffle direction. That profile was then counted for cell membrane features (peaks) of amplitude greater than 15 nm of probe deflection. Each cell was measured for four profiles, resulting in a total (sum) of ruffles per cell. Five cells were selected per image (two images per cell type) and then averaged for a mean # of ruffles per cell.

### FRET analysis of RAP1/2 activation status and vinculin tension biosensor

For FRET analysis of RAP1/2 activation status, hESCs were transfected with RAP1A and 2 A FRET-based biosensor probes derived from original plasmids obtained from Dr. Hahn and Dr. O’Shaughnessy, University of North Carolina (UNC) Chapel Hill^89^.

For the vinculin tension biosensor, human NPCs were transfected with vinculin tension biosensor using lentiviral particles as described above. Forty-eight hours after transduction, cells were sorted by FACS (BD FACSAria IIu). Media was then supplemented with CloneR (Stem-Cell Technologies) and cells were expanded for the imaging experiment.

Analysis of FRET was performed by live-cell FRET imaging using Leica SP5 DMI confocal microscope equipped with temperature and CO_2_ control^45^ at the Mount Sinai microscopy core. eCFP was excited with a 458 nm emission line using an Argon ion laser and eYFP was excited with a 515 nm emission line. eCFP, FRET, and eYFP channels were simultaneously imaged using an internal GaAsp detector collecting the emission from a range of wavelengths appropriate for each channel: For eCFP channel: 463–520 nm, FRET channel: 520–620 nm, and eYFP channel: 520–620 nm. Intensity-based ratiometric FRET indices and heatmaps were obtained on a per-cell (for RAP1/2) and per-focal adhesion (vinculin tension biosensor) basis using custom-written scripts in Matlab and ImageJ plugin (FRET Analyzer). For RAP1/2 probes, images were sorted into donor, FRET, and acceptor channels. Masks were drawn on top of the membrane covering both sides of the peri membrane space. For each set of images, the values for different channels were registered and the final FRET index was obtained^90^ by correcting the bleed-through using the equation $\frac{R={{{{{{\mathrm{FRET}}}}}}}-\left(\alpha \times {Cy{{{{{\mathrm{Pet}}}}}}}\right)-\left(\beta \times {Y{{{{{\mathrm{Pet}}}}}}}\right)}{{Cy{{{{{\mathrm{Pet}}}}}}}}$.

For the vinculin tension sensor, to obtain FA masks, CLAHE and Laplacian of Gaussian (LoG) were performed on the eYFP channel, followed by applying a size and intensity filter on the resulting particles. Since the vinculin tension biosensor is a single-chain construct, FRET Index was calculated by dividing the FRET channel (donor excitation with acceptor emission) by the CFP channel (donor excitation with donor emission).

### Molecular dynamics (MD) models

The computational model consists of simulating the mechanical rigidity of a cell membrane based on using an AFM tip to measure force as a function of its deformation. MD models with the approximation of macromolecular systems^52,53^ were used to simulate the dynamics of a cell composed of membranes and actin filaments in two dimensions.

#### Cell model

We developed a membrane model that can reproduce the main features of the mechanical structure of a cell. The cell membrane, nuclear membrane, and actin filaments are modeled as being formed by beads connected by springs. Specifically, the cell membrane is composed of 201 beads connected by springs forming a diameter of 5.25 µm. To describe the nuclear membrane, it was necessary to use 33 beads connected by springs, generating a diameter of 1.5 µm for the nucleus. Finally, the actin filaments, which are radial in the initial position, are separated into two types: type I filament has one end attached to the nuclear membrane and one end free, while type II filament has both ends free. For each type I configuration, we have two type II configurations interspersed, as depicted in Fig. 5c. The type I filament contains ten beads with a total length of 1.5 µm, while type II contains eight beads and a length of 1.2 µm.

To facilitate the simulation, each bead was labeled with one of three different numerical marks. All beads that make up the cell membrane are marked as 0. The beads forming the actin filament and nuclear membrane were marked as 1. The ends of actin filaments near the cell membrane were labeled as 3. The elastic constants responsible for the bonds between each bead can be separated into three types: the elastic constant between the beads that make up the membranes (referred to as *κ*_*F*00_); the elastic constant that forms the actin filaments (*κ*_*F*11_), except the head next to the cell membrane; and the elastic constant between the head of the filament and the nearest membrane bead (*κ*_*F*13_). The relation between the constant modules in the simulation is given by: *κ*_*F*11_ = *κ*_*F*00_ and *κ*_*F*13_ = 5*κ*_*F*11_.

#### Potential model

To calculate the forces capable of creating the basic structure of a cell, it is necessary to define the potential of attraction and repulsion between the beads of type 0, 1, and 3 (see above).

We based our system on a Bead-Spring model of polymers, also known as the coarse-grained model. In this model, we have the following potentials based on corresponding equations:

Lennard-Jones (L-J) potential (1), where the parameter r is the distance between particles (beads), while *σ* is the equilibrium distance, and *ɛ* the well depth energy.

1 $${{{{{{\rm{U}}}}}}}_{{{{{{\rm{LJ}}}}}}}\left(r\right)=\varepsilon \left[{\left(\frac{\sigma }{r}\right)}^{12}-2{\left(\frac{\sigma }{r}\right)}^{6}\right]$$

Weeks–Chandler–Anderson (WCA) (2), representing the repulsive part of L-J potential:

2 $${{{{{{\rm{U}}}}}}}_{{{{{{\rm{WCA}}}}}}}\left(r\right)=\left\{\begin{array}{c}{{{{{{\rm{U}}}}}}}_{{{{{{\rm{LJ}}}}}}}\left(r\right)+\varepsilon ,{{{{{\rm{if}}}}}}\;r \, < \, \sigma \\ 0,{{{{{\rm{if}}}}}}r\ge \sigma \end{array}\right\}$$

Finite extensible nonlinear elastic (FENE) potential (3), where *κ*_F_ is the FENE spring constant, ∆*r*^max^ is a maximal extension/compression, and *r*_0_ is equilibrium position.

3 $${{{{{{\rm{U}}}}}}}_{{{{{{\rm{FENE}}}}}}}\left(r\right)=\frac{-{\kappa }_{F}{\left(\varDelta {r}_{{^{max} }}\right)}^{2}}{2}{{\log }}\left[1-{\left(\frac{r-{r}_{0}}{\varDelta {r}_{{^{max} }}}\right)}^{2}\right]$$

Bend potential (4), related to torsional potential. It represents the energy associated with bending between three neighboring beads and *κ* is the bend spring constant, where θ is the angle between three consecutive neighbor beads and θ_0_ is the equilibrium angle.

4 $${{{{{{\rm{U}}}}}}}_{{{{{{\rm{BEND}}}}}}}\left(\theta \right)=\frac{{\kappa }_{B}}{2}{\left({{\cos }}\left(\theta \right)-{{\cos }}\left({\theta }_{0}\right)\right)}^{2}$$

The potentials are shown as a function of the distances between beads for various values of *κ*_*Fij*_, *κ*_*Bij*_*, ɛ*_*ij*_, *σ*_*ij*_, *∆r* ^max^_*ij*_, with *i, j* = 0, 1, 3 (types of beads, see above) (Fig. S14). All quantities are dimensionless and are represented by the asterisk symbol.

#### AFM tip model

To model the tip of an AFM, we used two elastic constants, one in x direction (*k*_x_) and one in *y* direction (*k*_y_), noting that we are working on a 2-D problem. The spring are clamped into an apparatus and the assembly moves in the direction of the cell membrane with constant velocity V. The force on the membrane is measured as a function of the deformations of the springs. The tip of the AFM is shaped like a bead and marked as 7.

#### Numerical simulation

With the definitions given above and appropriately choosing the parameters for *σ*, it is possible to analyze the stiffness behavior of the cell using the AFM tip. The proposed model allows us to attract energy between the membranes (*ɛ*_*00*_) and also the attraction energy that the actin filament head makes in the membrane (*ɛ*_*03*_). The results of the force made by the AFM tip on the membrane as a function of its distance traveled, for both cases, are shown in graphs in figures. To facilitate analysis both graphs were plotted on the same scale.

### Statistics and reproducibility

Data were presented as mean ± SEM unless indicated otherwise. One-way analysis of variance (ANOVA) with Tukey’s or Dunnett’s post hoc test or Kruskal–Wallis test with Dunn’s multiple comparisons test were performed for data with more than two groups. Unpaired *t*-test (two-sided) or Mann–Whitney test was applied when comparing data from two groups. For studies with repeated measures (RM), a two-way ANOVA RM and Bonferroni post hoc test was performed. Statistical analyses were performed using GraphPad Prism 8 statistical software. Statistical significance was considered as *P* < 0.05 (*); *P* < 0.01 (**); *P* < 0.001 (***). Sample sizes and statistical details are indicated in figure legends.

All cell culture experiments presented in the manuscript were repeated at least three times independently with similar results.

### Reporting Summary

Further information on research design is available in the Nature Research Reporting Summary linked to this article.

## Supplementary information

Supplementary Information

Description of Additional Supplementary Files

Supplementary Data 1

Supplementary Movie 1

Supplementary Movie 2

Supplementary Movie 3

Reporting Summary

## Data Availability

The RNA-seq data generated in this study have been deposited in the NCBI Gene Expression Omnibus (GEO) database under accession code “GSE158017”. The hg38 human reference genome was obtained from Ensembl Release 90 (https://useast.ensembl.org/index.html). Source data underlying graphs and full WB images are provided in Source Data. All other data that support the findings of this study are available from the corresponding author R.H.F. upon reasonable request. Source data are provided with this paper.

## Source data

Source Data

## Code availability

Molecular dynamics (MD) codes have been deposited at Github (github.com/diasrodri/SimCellMD-1) and Zenodo (zenodo.org/record/4977917#.YMyfWTZKjyU)^91^.
