# Supplementary material for: Plexin-B2 orchestrates collective stem cell dynamics via actomyosin contractility, cytoskeletal tension and adhesion
Source: Nat Commun. 2021 Oct 14;12:6019. doi: 10.1038/s41467-021-26296-7 (PMC8517024; doi:10.1038/s41467-021-26296-7)
Supplement: Supplementary file 1 — Supplementary Information [file 41467_2021_26296_MOESM1_ESM.pdf]

SUPPLEMENTARY INFORMATION

Plexin-B2 orchestrates collective stem cell dynamics via actomyosin contractility, cytoskeletal tension and adhesion

Chrystian Junqueira Alves et al.

Supplementary Figures.

On following pages.

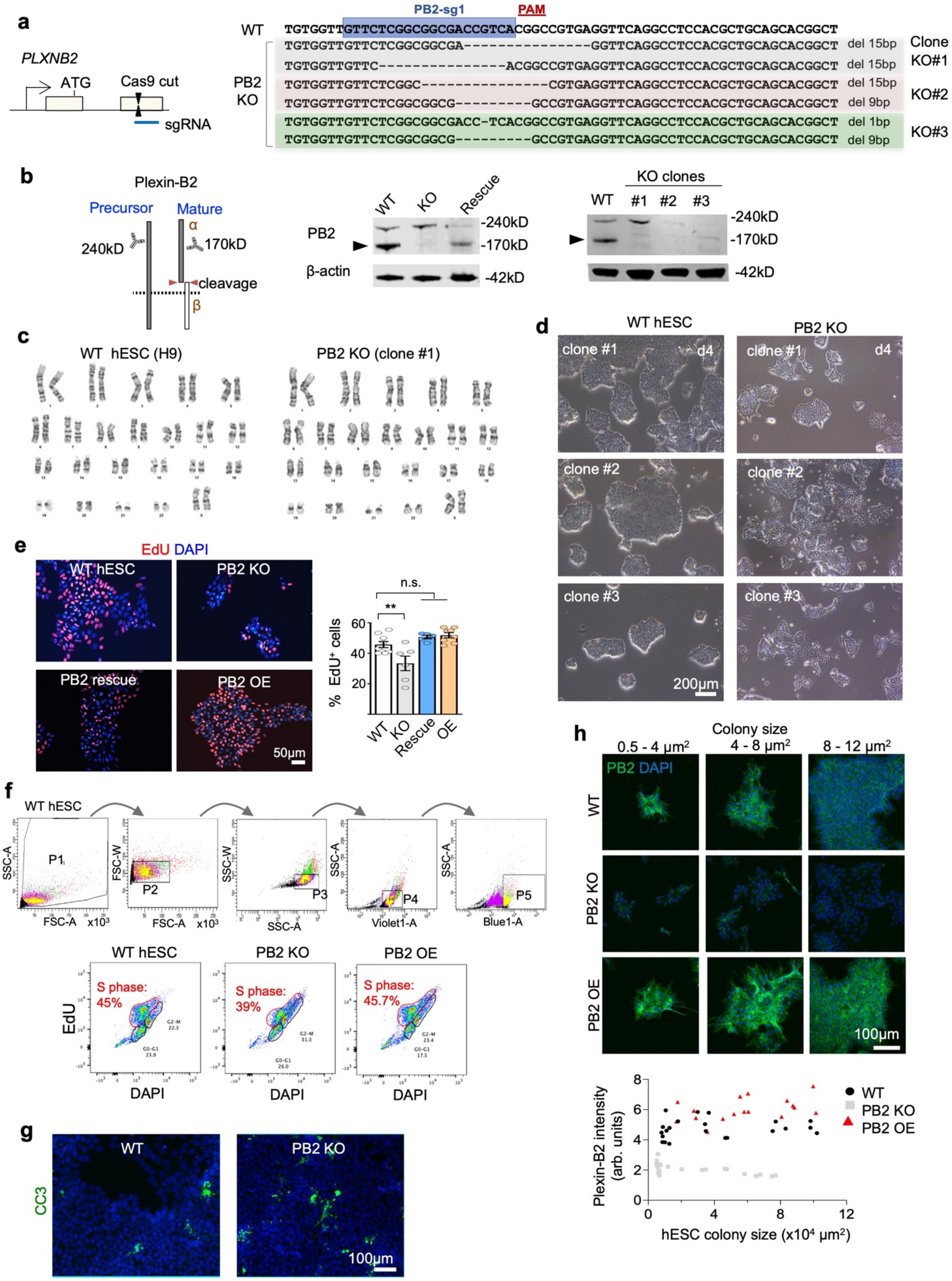

Supplementary Figure 1. Plexin-B2 deletion affects hESC colony expansion.

a, Left, schematic of CRISPR/Cas9-mediated *PLXNB2* knockout (KO) with a small guide RNA (sgRNA) targeting second coding exon. Right, sequencing of three KO clones reveals bi-allelic frameshift or in-frame deletion mutations in each clone. PB2-sg1, small guide RNA targeting *PLXNB2.* PAM, protospacer adjacent motif.

b, Left, diagram of Plexin-B2 forms: precursor and mature protein (non-covalently linked α and β subunits). Antibody

symbols indicate fragments detected on WB. Right, WBs show loss of mature Plexin-B2 (arrowhead) in polyclonal population and three clonal KO lines, which was rescued by expression of CRISPR-resistant *PLXNB2*. Note that a Plexin-B2 precursor band carrying in-frame CRISPR-mutations (~240 kD) is detectable on WB, but these mutant precursor forms are not processed to mature protein with cell surface localization (see IF data in Fig. 1a).

c, Karyotypes of WT hESCs and of *PLXNB2* KO clones were normal (clone #1 shown).

d, Phase-contrast images show smaller colony size for three *PLXNB2* KO clones compared to WT clones (day 4 after plating). Similar results were obtained from 3 independent cultures.

e, IF images and quantification of EdU pulse (30 min) study reveal reduced proliferation of *PLXNB2* KO hESCs. One-way ANOVA followed by Dunnett's multiple comparisons test versus WT. n=1,235 cells for WT, n=593 cells for *PLXNB2* KO, n=1,050 cells for Rescue, and n=2,047 cells for *PLXNB2* OE. $^{**}P=0.0069$; n.s., not significant. Data represent mean ± SEM.

f, Top**,** example of gating strategy of flow cytometry to select DAPI$^+$ (violet laser) and EdU$^+$ cells (Blue laser). Bottom, flow cytometry data for ESCs of three genotypes show reduced fraction of cells in S phase for PB2 KO compared to WT and PB2 OE cells.

g, IF micrographs show comparable rates of apoptosis (cleaved caspase 3, CC3) in WT and *PLXNB2* KO hESCs.

h, Plexin-B2 expression levels remain relatively constant during hESC colony expansion. Cells were plated at 10^4 cells per well of an 8-well chamber slide and analyzed after 2 and 5 days for study of size and Plexin-B2 immunointensity. Arb. units, arbitrary units.

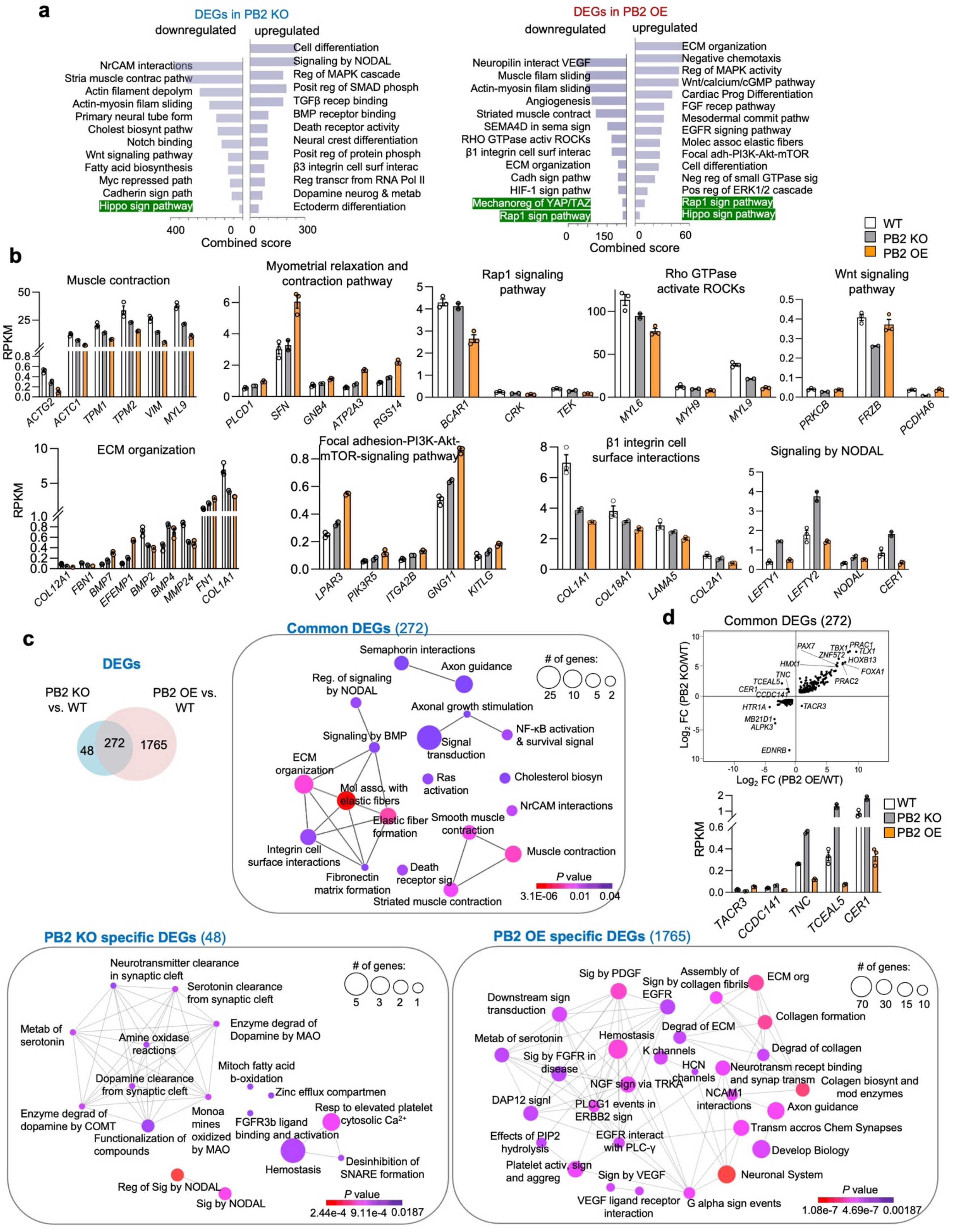

Supplementary Figure 2. Plexin-B2 KO affects gene pathways related to cell biomechanics and differentiation.

a, Enriched gene ontologies and pathways of differentially expressed genes (DEGs) in *PLXNB2* KO and OE hESCs relative to WT by ENRICHR analyses. Colors highlights gene sets associated with Rap1 as well as Hippo and YAP/TAZ mechanosensing pathways.

b, Expression levels of genes of selected GO categories that were differentially regulated between *PLXNB2* KO or OE

hESCs relative to WT. Average RPKM (reads per kb per million reads) values for each genotype condition are shown. n=3 samples for WT and PB2 OE, and n=2 for PB2 KO. Data represent mean ± SEM.

c, Top left, Venn diagram displaying unique and common DEGs in *PLXNB2* KO or OE hESCs relative to WT. Network view of enriched Reactome terms show pathways for shared and specific DEGs for *PLXNB2* KO and OE hESCs. Note that cell-mechanics related signaling pathways were highly represented.

d, Top, Scatter plot showing relative expression changes of DEGs common to *PLXNB2* KO and OE hESCs relative to WT. Selected top DEGs are indicated. Bottom, Expression levels of DEGs with opposite directionality of expression changes in *PLXNB2* KO and OE hESCs relative to WT. n=3 samples for WT and PB2 OE, and n=2 for PB2 KO. Data represent mean ± SEM.

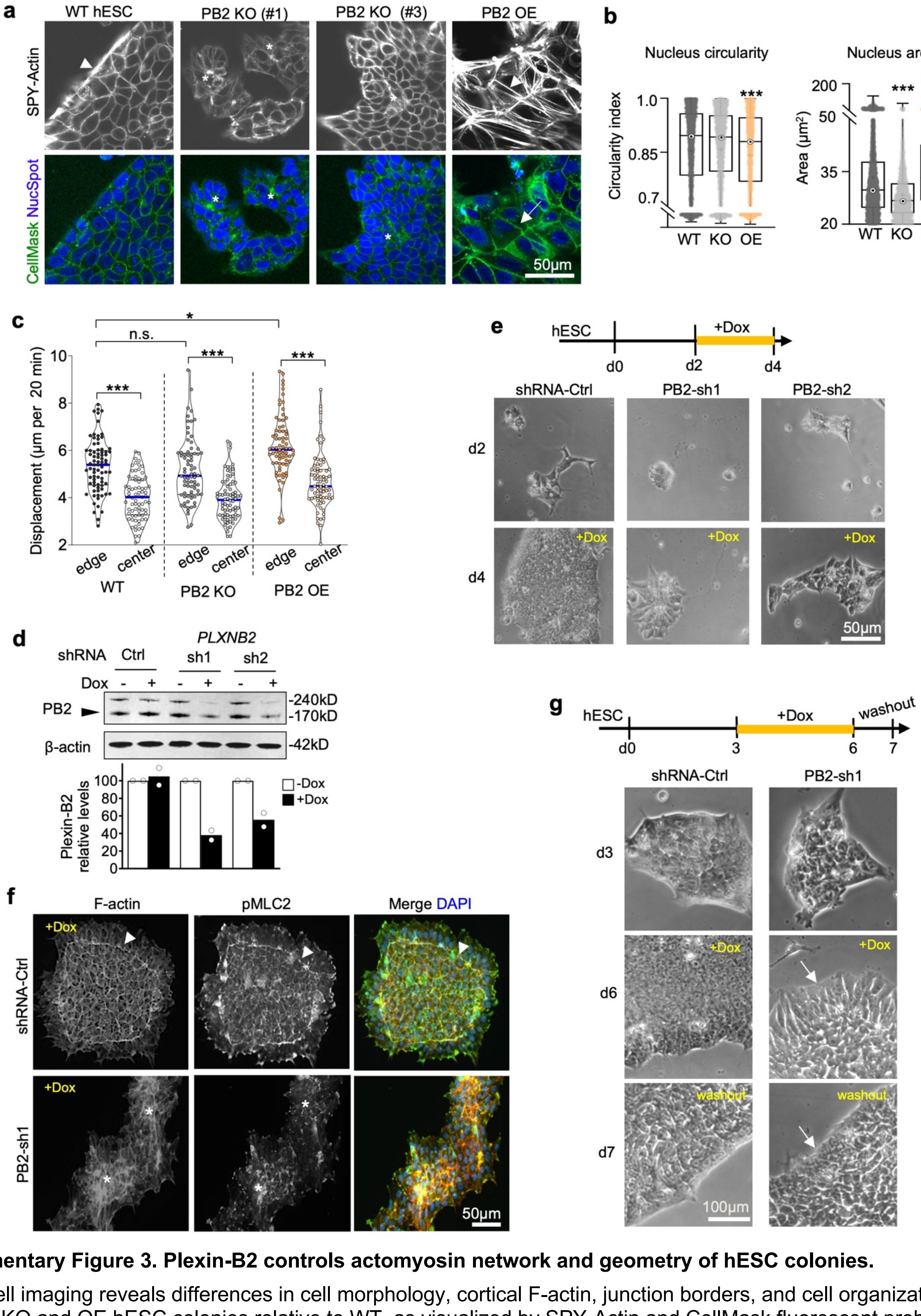

Supplementary Figure 3. Plexin-B2 controls actomyosin network and geometry of hESC colonies.

a, Live-cell imaging reveals differences in cell morphology, cortical F-actin, junction borders, and cell organization in *PLXNB2* KO and OE hESC colonies relative to WT, as visualized by SPY-Actin and CellMask fluorescent probes. Nuclei were labeled with NucSpot. Arrowhead points to peripheral F-actin band in WT colony. Asterisks denote abnormal cell clusters in *PLXNB2* KO colonies. Arrow points to strengthened cortical F-actin and stretched cellular borders, often associated with F-actin stress fibers in OE colonies.

b, Box plots show high content quantifications of nuclei geometries (area and circularity) in WT, PLXNB2 KO and OE hESCs. Box plots show 25-75% quantiles, median (bullseye), mean (cross sign), minimal and maximal values (whiskers).

For nuclear area: n=9,620 nuclei for WT, n=3,757 for KO, and n=3,292 for OE. For circularity: n=6,072 nuclei for WT, n=4,695 for KO, and n=3,590 for OE. Data collected from 30 random fields per group. Kruskal-Wallis test followed by Dunn's multiple comparisons test. ****P*<0.0001.

c, Quantification of cell movement in hESC colonies. Cells at colony edge show significantly faster changes in their position than cells in colony center. Interestingly, Plexin-B2 OE leads to an increased rate of cell displacement at colony periphery. Kruskal-Wallis test followed by Dunn's multiple comparisons test. n=75 cells for each genotype in each condition. **P*<0.05; ****P*<0.001; n.s., not significant. Blue lines indicate median, gray lines denote 25% and 75% percentiles.

d, WB shows Plexin-B2 knockdown efficiency with two independent doxycycline (Dox)-inducible shRNAs. β-actin served as loading control. Arrowhead points to band representing mature Plexin-B2 (PB2). Quantification shown below. n=2 samples per group. Data represent mean.

e, Top, experimental timeline of Dox-induced *PLXNB2* shRNA KD in hESCs. Bottom, phase-contrast images show comparable initial formation of small colonies 2 days after passage with control (Ctrl) or Plexin-B2 shRNAs. At day 4, two days after Dox induction, *PLXNB2* KD resulted in smaller colonies with altered geometry.

f, Fluorescence images show actomyosin band (arrowheads) at colony periphery of WT hESCs, which was dissipated with *PLXNB2* KD. Also note cellular disorganization and abnormal cell clusters (denoted by asterisks) in KD colony.

g, Top, timeline of reversible shRNA KD of *PLXNB2* during hESC colony formation. Bottom, phase-contrast images of hESC colonies show that 3 days after *PLXNB2* KD (d6), cells displayed elongated morphology at colony edge (arrow), and 1 day after Dox washout (d7), cells returned to rounded morphology (arrow).

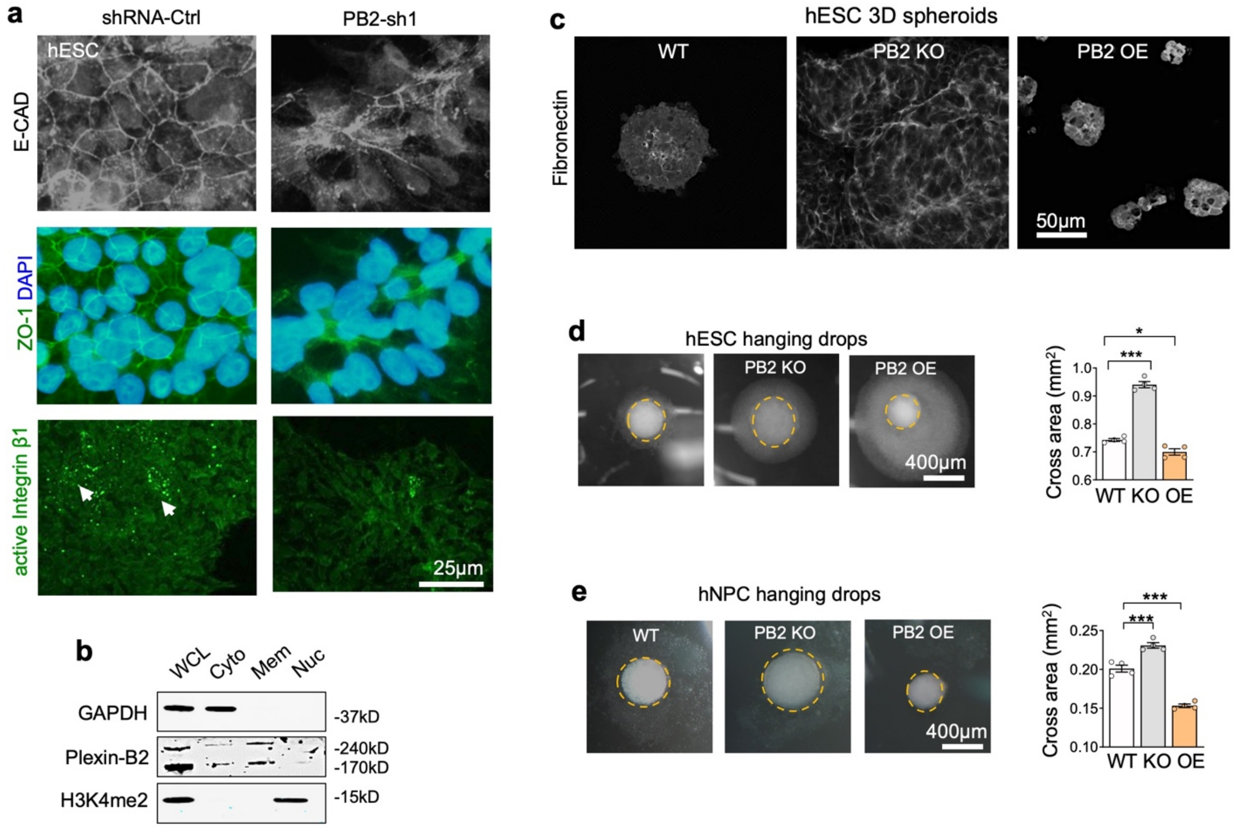

Supplementary Figure 4. Plexin-B2 stabilizes adherens junction and focal adhesion complexes.

a, Confocal images show even recruitment of E-Cadherin (E-CAD) and ZO-1 to cell junctions in control hESC colony; both in disarray and reduced in *PLXNB2* KD colonies, as was activate integrin β1 (labeled with antibody HUTS-4, dotty appearance denoted by arrows).

b, WBs verified cell fractionation: GAPDH for cytoplasm (Cyto) fraction, Plexin-B2 for cell membrane (Mem), and Histone 3 with lysine 4 dimethylation (H3K4me2) for nuclear (Nuc) fraction. WCL, whole cell lysate.

c, Cross-section images of 3D hESC aggregates show that *PLXNB2* KO resulted in cellular disarray and disorganized fibronectin deposits, whereas *PLXNB2* OE led to more compact fibronectin network.

d, e, Images of aggregates of hESC (**d**) or hNPCs (**e**) in hanging drop culture show reduced or increased compaction of *PLXNB2* KO or OE cells compared to WT, respectively. Dotted lines outline aggregate compaction. Quantification on the right. One-way ANOVA followed by Dunnett's post-hoc test. n=4 aggregates per group. For hESC: *P=0.023 and ***P<0.0001. For hNPC: ***P=0.0004 (WT vs. KO) and ***P<0.0001 (WT vs. OE). Data represent mean ± SEM.

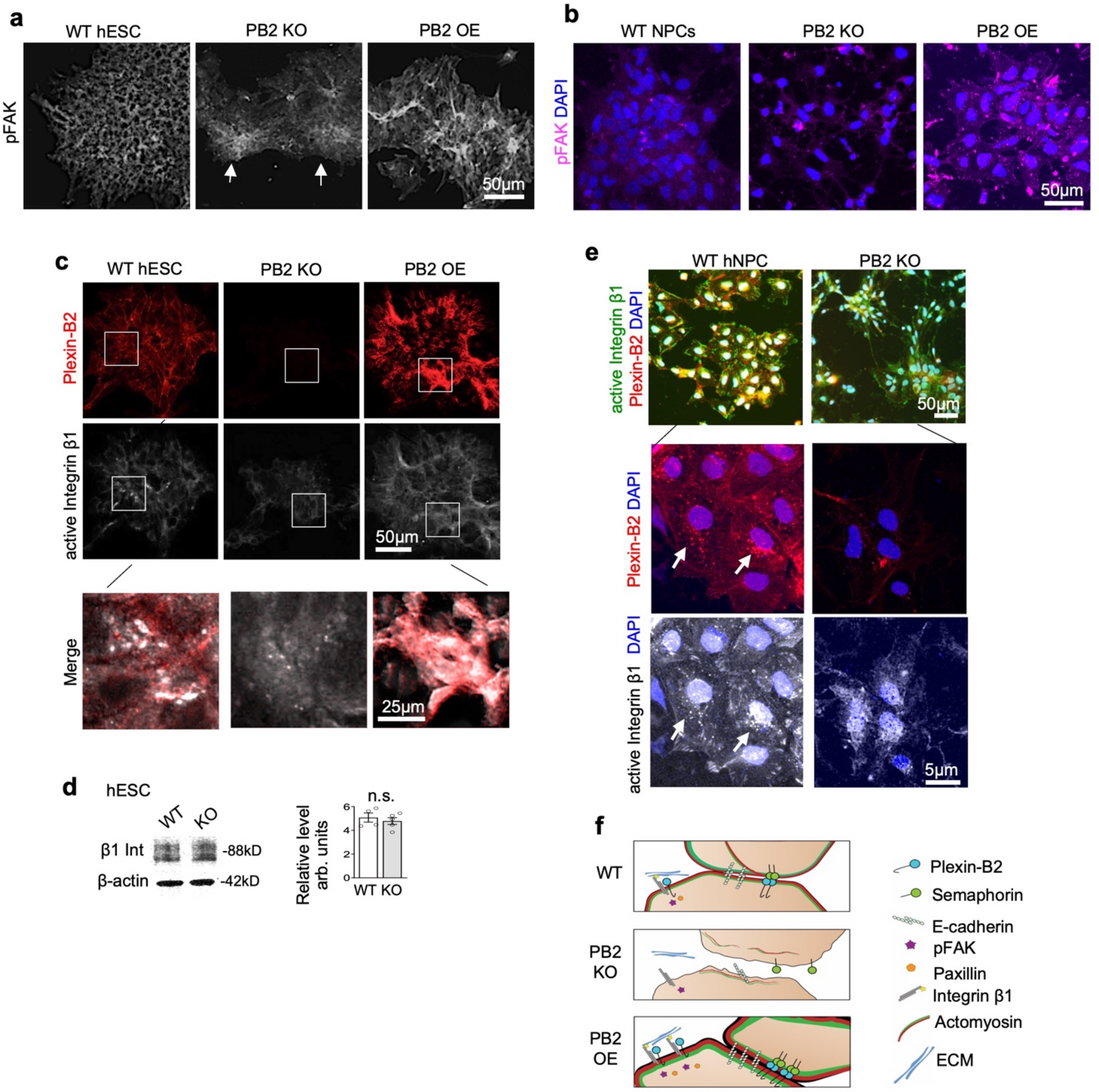

Supplementary Figure 5. Plexin-B2 enhances active integrin β1 and focal adhesions.

a, b, IF images show redistribution of focal adhesion kinase (pFAK) in mutant hESC colonies (**a**). Arrows point to the foci appearance in *PLXNB2* KO colonies, in contrast to the homogenous distribution in WT. Confocal images of hNPCs (**b**) show enhanced signal for pFAK in *PLXNB2* OE cells.

c, IF images show surface expression of Plexin-B2 and active integrin β1 in WT hESC colonies (HUTS-4 antibody), both reduced or enhanced in *PLXNB2* KO or OE hESCs, respectively. Enlarged images of boxed areas are shown in bottom panels, highlighting the dotty and clustered appearance of active integrin β1 in WT and OE cells, respectively. Immunosignals of active integrin β1 were decreased in *PLXNB2* KO.

d, WBs show comparable levels of integrin β1 in WT and *PLXNB2* KO hESCs. Quantification below. β-actin served as loading control. arb. units, arbitrary units. Two-sided unpaired *t*-test. n=4 samples for WT, n=5 for KO. n.s., not significant. Data represent mean ± SEM.

e, IF images of hNPCs (magnified images below) show that *PLXNB2* KO resulted in altered cell morphology, cell size, and reduced levels of active integrin β1 (HUTS-4 antibody, dotty appearance denoted by arrows in WT cells).

f, Model of Plexin-B2 controlling actomyosin contractility in cells, which in turn impacts adhesive properties, e.g., cadherin-based junctional adhesion and integrin-mediated cell-matrix attachment.

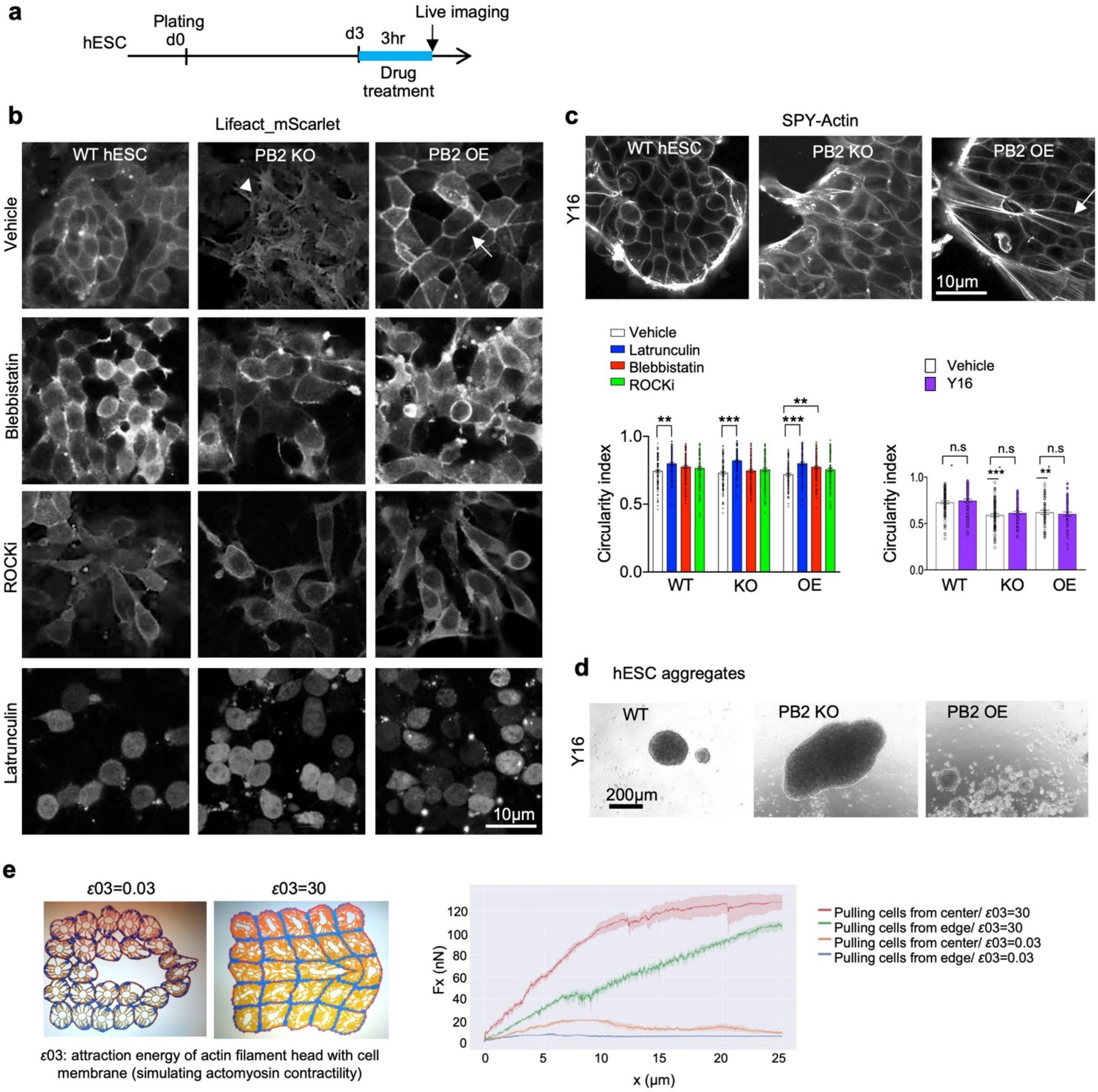

Supplementary Figure 6. Plexin-B2 regulates mechanical properties of hESC colonies.

a, Experimental timeline for live-cell imaging of hESC colonies from 3 hours after drug treatment.

b, Confocal images from live-cell videography of hESC colonies treated with indicated inhibitors. F-actin network was visualized by mScarlet-tagged LifeAct, and cell morphology and junctional borders were seen by CellMask dye. Arrowhead points to cell protrusions from colony edge for *PLXNB2* KO or after drug treatment. Arrow points to tensed cortical F-actin and stretched cell border in Plexin-B2 OE colony. Quantifications of cell morphology using circularity index shown to the right. One-way ANOVA followed by Dunnett's multiple comparisons test. n=100 cells per group. ***P*=0.0011 (WT veh vs. WT latrunc), ***P*=0.0014 (PB2 OE veh vs. PB2 OE blebb), ****P*<0.0001. Data represent mean ± SEM.

c, Top, confocal images from hESC colonies treated with Y16 (inhibitor of PDZ-Rho-GEF). F-actin visualized with SPY555-Actin. Arrow points to tensed cortical F-actin and stretched cell border in Plexin-B2 OE colony. One-way ANOVA followed by Tukey's multiple comparisons test. For vehicle treatment, n=84, 78, 48 cells for WT, KO, OE, respectively. For Y-16: n=90, 56, 52 for WT, KO, OE, respectively. ***P*=0.0013 and ****P*<0.0001. n.s., non-statistically significant. Data represent mean ± SEM.

d, Images of 3D aggregates of indicated hESCs show no overt effect of Y16 inhibitor.

e, Mathematical simulation of the impact of different levels of intracellular contractility on intercellular cohesion in multicellular organization. Left, intracellular contractility was modulated by varying attraction energy of the actin filament head with the cell membrane (ϵ03). ϵ03=0.03 simulated reduced cell stiffness as seen in *PLXNB2* KO, while ϵ03=30 simulated enhanced cell stiffness as seen in *PLXNB2* OE. Right, intercellular cohesiveness was interrogated by measuring

the force needed to pull cells away from the colony edge or from colony center. The lines represent mean, the shadowed area ± SD.

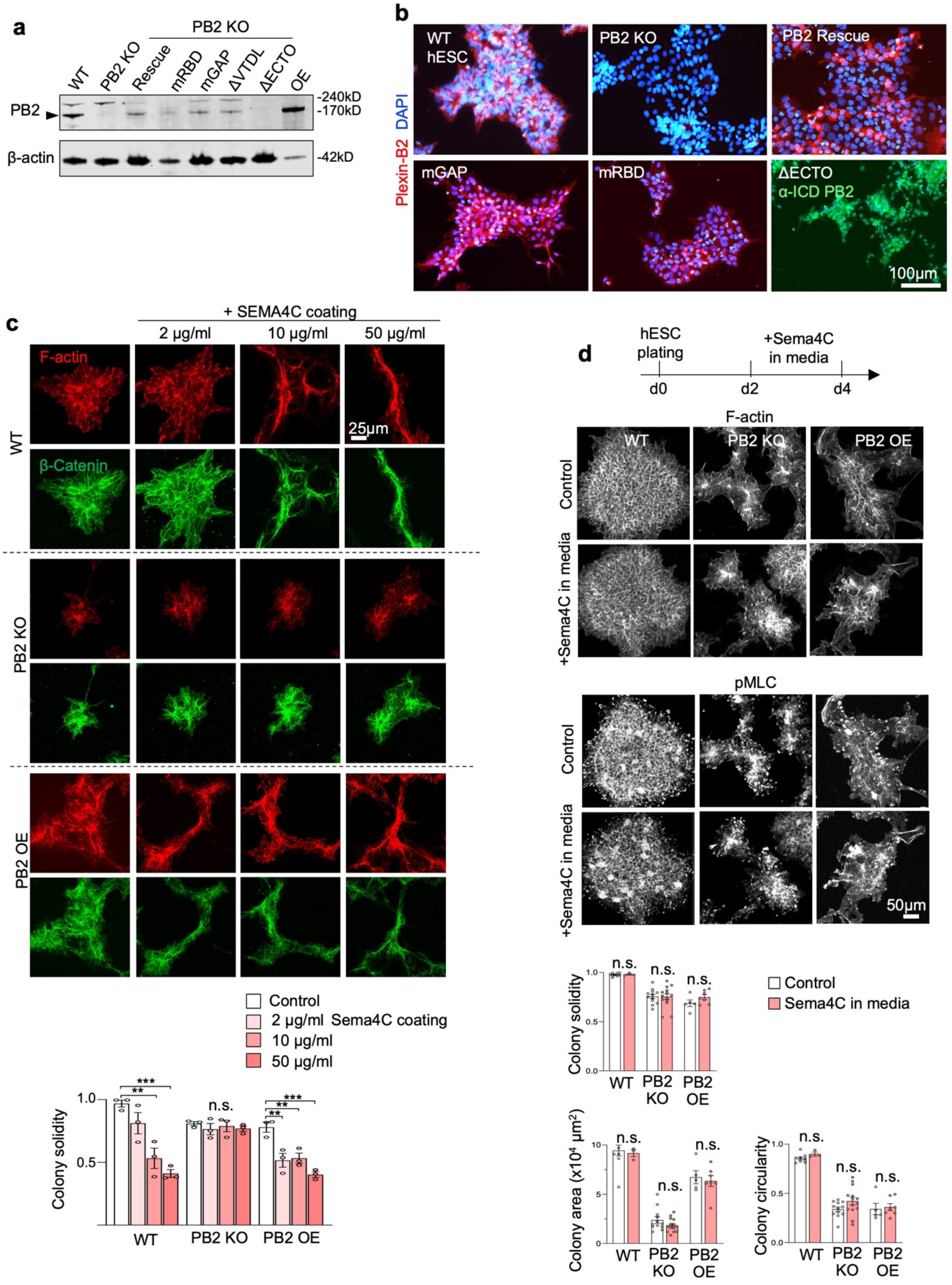

Supplementary Figure 7. Interrogation of Plexin-B2 signaling pathways.

a, WB demonstrates comparable expression levels of Plexin-B2 signaling mutants. Arrowhead points to mature Plexin-B2 band. Note the absence of signal for Plexin-B2-ΔECTO mutant, as the Plexin-B2 antibody detects epitopes in the extracellular domain (ECD) only. Rescue denotes lentiviral expression of CRISPR-resistant Plexin-B2 in the background of *PLXNB2* KO, while overexpression (OE) denotes lentiviral expression of WT Plexin-B2 in WT hESCs.

b, IF images show expression of Plexin-B2 signaling mutants in hESCs. Note that a different antibody against the intracellular domain (ICD) of Plexin-B2 was used for cells expressing the ΔECTO mutant.

c, Increasing concentrations Sema4C-Fc were mixed with Matrigel for coating of cell culture dishes before seeding of

hESCs. A concentration-dependent effect of Sema4C on colony geometry was observed for WT and Plexin-B2 OE cells. In contrast, Plexin-B2 KO cells appeared insensitive to Sema4C. n=3 colonies per group. One-way ANOVA followed by Dunnett's multiple comparisons to compare treatments within the groups. For WT group: ***P*=0.0029 and ****P*=0.0006. For PB2 OE group: ***P*=0.0049 (control vs. 2μg), ***P*=0.0071 (control vs. 10μg), ****P*=0.0005. n.s., not significant. Data represent mean ± SEM.

d, Fluorescent images and quantifications show that application of soluble Sema4C-Fc (2 μg/ml) in culture media for 2 days did not alter colony geometry or actomyosin networks in either of the three genotypes. For control: n=9, 12, 5 colonies for WT, KO and OE, respectively. For Sema4C treatment: n=3, 14, 7 for WT, KO and OE, respectively. Two-sided unpaired *t*-test to compare treatments within the groups. n.s., not significant. Data represent mean ± SEM.

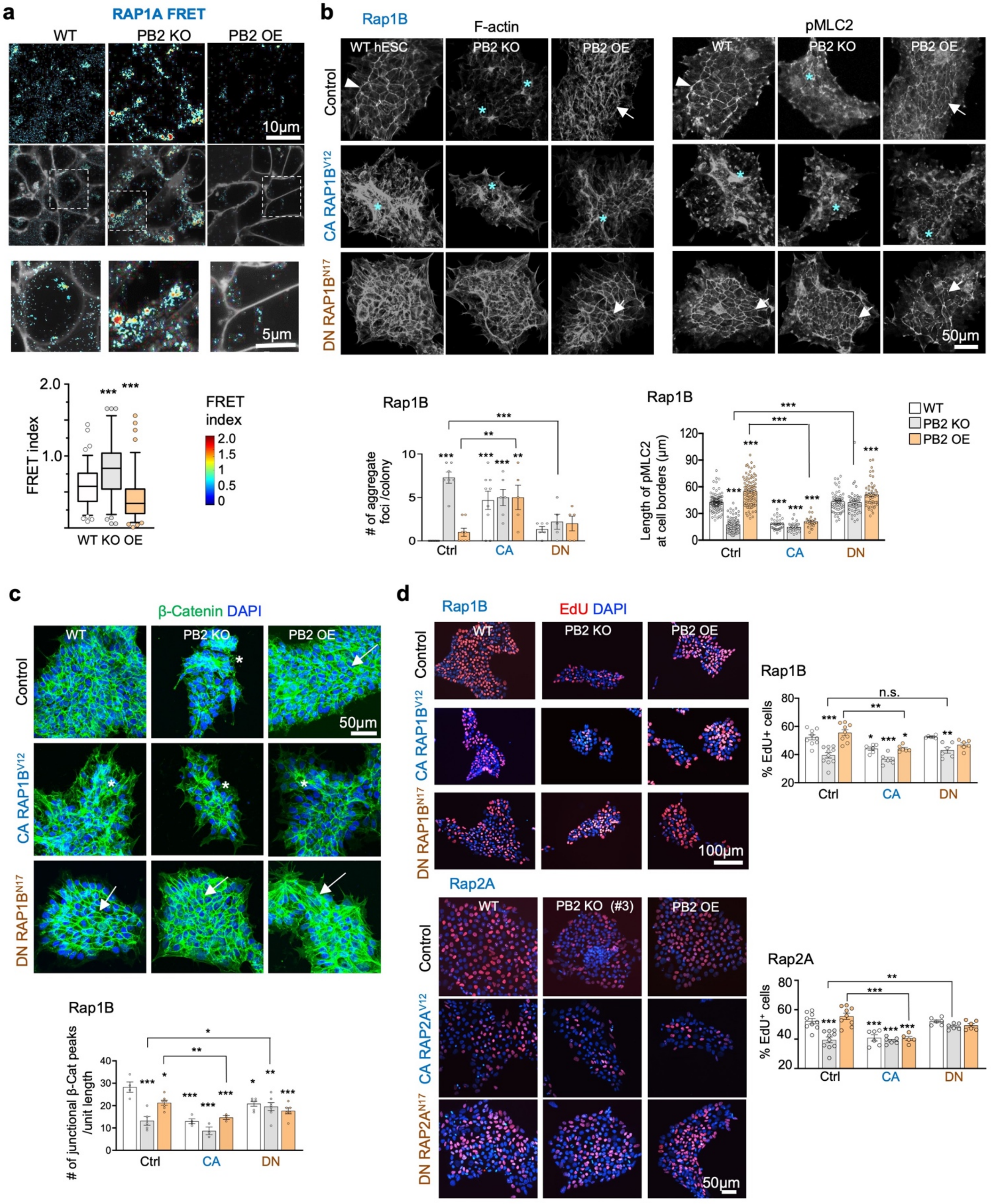

Supplementary Figure 8. Effects of Rap signaling on cytoskeletal organization and proliferation of hESC colonies.

a, Representative images of normalized RAP1A FRET signals in indicated hESCs. Enlarged images of boxed areas are shown below. Quantifications of FRET index are shown as box plots (median, 25-75 quantile, and top and bottom 5% data points). Kruskal-Wallis test followed by Dunn's multiple comparisons test. n=91-95 for each genotype, ****P*=0.0002 (WT vs. KO) and ****P*<0.0001 (WT vs. OE).

b, Confocal images show the effects of RAP1B isoforms on cell morphology and actomyosin network in indicated hESC colonies. Note that constitutive active (CA) RAP1B^{V12} phenocopied Plexin-B2 KO, whereas dominant negative (DN) RAP1B^{N17} phenocopied Plexin-B2 OE. Arrowhead points to peripheral F-actin band in WT colony, asterisks denote abnormal cell clusters in *PLXNB2* KO colony, and arrows point to strengthened cortical F-actin, stress fibers, and stretched

junction borders in *PLXNB2* OE colony. Also note that CA RAP1B attenuated Plexin-B2 OE phenotypes, while DN RAP1B attenuated Plexin KO phenotypes. Quantifications are shown below. One-way ANOVA followed by Dunnett's post hoc correction to compare to WT group. Two-sided unpaired *t*-test was applied when comparing data from two groups (brackets). For aggregate foci: control, n=5-9 colonies for each genotype in each condition. ***P*=0.0081 (Control PB2 OE vs. CA RAP1B^{V12} PB2 OE), ***P*=0.001 (WT vs. CA RAP1B^{V12} PB2 OE), ****P*<0.0001 (WT vs. PB2 KO), ****P*=0.0003 (WT vs. CA RAP1B^{V12} WT), ****P*=0.0005 (WT vs. CA RAP1B^{V12} PB2 KO), and ****P*=0.0007 (PB2 KO vs. DN RAP1B^{N17} PB2 KO). For pMLC2: control, n=80-100 cell borders for each genotype; CA RAP1B^{V12}, n=20-30 for each genotype; DN RAP1B^{N17}, n=50-60 for each genotype. ****P*=0.0005 (WT vs. DN RAP1B^{N17} PB2 OE) and ****P*<0.0001. Data represent mean ± SEM.

c, Confocal images show the effects of RAP1B isoforms on junctional recruitment of β-catenin in hESC colonies. Constitutively active (CA) RAP1B-V12 phenocopied Plexin-B2 KO, whereas dominant negative (DN) RAP1B-N17 phenocopied Plexin-B2 OE. Asterisks denote abnormal cell clusters in *PLXNB2* KO colony and arrows point to stretched junction borders in *PLXNB2* OE colony. Quantifications shown below. One-way ANOVA followed by Dunnett's post hoc correction to compare to WT group. Two-sided unpaired *t*-test was applied when comparing data from two groups (brackets). n=3-7 colonies for each genotype in each condition. **P*=0.041 (PB2 KO vs. DN RAP1B^{N17} PB2 KO), **P*=0.021 (WT vs. PB2 OE), **P*=0.016 (WT vs. DN RAP1B^{N17} WT), ***P*=0.0028 (WT vs. DN RAP1B^{N17} PB2 KO), ***P*=0.0068 (PB2 OE vs. CA RAP1B^{V12} PB2 OE) ****P*=0.0001 (WT vs. CA RAP1B^{V12} PB2 OE), ****P*=0.0004 (WT vs. DN RAP1B^{N17} PB2 OE), ****P*<0.0001. Data represent mean ± SEM.

d, Representative IF images and quantifications of EdU labeling (30 min pulse) of hESCs expressing different RAP1/2 isoforms. Quantifications on the right show results for RAP1B or RAP2A and manipulations. One-way ANOVA followed by Dunnett's post hoc correction. For Rap1B and Rap2A: control, n=2319, 1815, 1603 cells for WT, KO and OE, respectively (note the control bar graphs represent the same datasets for both Rap1B and Rap2A). For CA RAP1B^{V12}, n=806, 640, 974; DN RAP1B^{N17}, WT, n=967, 975, 860 for WT, KO and OE, respectively. **P*=0.015 (WT vs. CA RAP1B^{V12} PB2 OE), **P*=0.016 (WT vs. CA RAP1B^{V12} WT), ***P*=0.0042 (WT vs. DN RAP1B^{N17} PB2 KO), ****P*<0.0001 (WT vs. CA RAP1B^{V12} PB2 KO), ****P*<0.0001 (WT vs. PB2 KO), n.s., not significant. For CA RAP2A^{V12}, n=859, 570, 528 cells; DN RAP2A^{N17}, n=1382, 1061, 1553 cells for WT, KO and OE, respectively. ****P*=0.0003 (WT vs. CA RAP2A^{V12} PB2 OE), ****P*<0.0001 (WT vs. CA RAP2A^{V12} PB2 KO), ****P*=0.0002 (WT vs. CA RAP2A^{V12} WT), and ****P*<0.0001 (WT vs. PB2 KO). Data represent mean ± SEM.

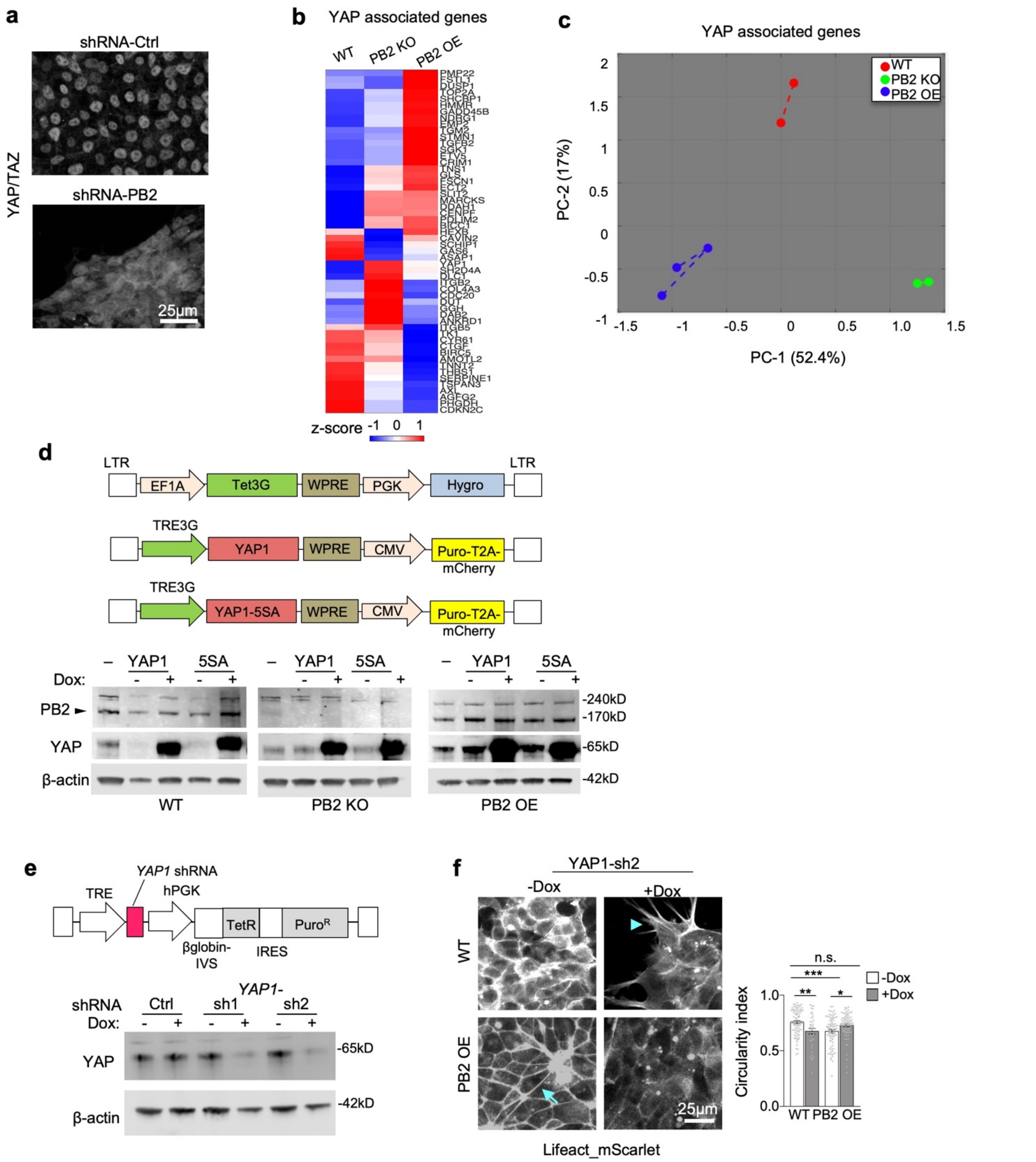

Supplementary Figure 9. Effects of modulating Plexin-B2 signaling on YAP signature gene expression and morphology of hESC colonies.

a, IF images of hESC colonies show that *PLXNB2* knockdown by shRNA resulted in a cytoplasm shift of YAP.

b, Heatmap of expression of 51 YAP signature genes (GSEA gene set M2871) in indicated hESCs.

c, Principal component analysis of hESC samples based on 51 YAP signature genes shows that different genotypes are represented in distinct clusters on the plot, indicating different YAP signaling states.

d, Top, diagram of lentiviral vectors for Dox-inducible expression of wild-type YAP1 or constitutively active YAP1-S5A. Tet3G: Tet-On 3G transactivator protein. TRE3G: Tetracycline response element. Bottom, WBs show Dox induced expression of *YAP1* or *YAP1*-5SA in hESC of different genotypes. Arrowhead indicates mature Plexin-B2. β-actin as loading control.

e, Top, diagram of lentiviral vector for Dox-inducible expression of shRNA against YAP1. Bottom, WB confirmed YAP1 knockdown by two independent shRNAs. β-actin as loading control.

f, Live-cell images hESC colonies labeled with Lifeact_mScarlet demonstrate the influence of Plexin-B2 OE and YAP KD on cortical F-actin, cell morphology, and cell border. Arrow points to strengthened cortical F-actin and stretched cellular border in Plexin-B2 OE colony, which was attenuated by Dox inducible *YAP1* KD. Arrowhead points to cell protrusion at edge of colony with *YAP1* KD. Right, quantification of cell circularity index. One-way ANOVA with Tukey's post hoc test. For WT, -Dox n=88 cells, +Dox n=38 cells. For *PLXNB2* OE, n=80 cells for each condition. **P*=0.030 (PB2 OE-YAP1-sh2 -Dox vs. PB2 OE-YAP1-sh2 +Dox), ***P*=0.0041 (WT-YAP1-sh2 -Dox vs. WT-YAP1-sh2 +Dox), and ****P*=0.0001 (WT-YAP1-sh2 -Dox vs. PB2 OE-YAP1-sh2 -Dox). n.s., not significant. Data represent mean ± SEM.

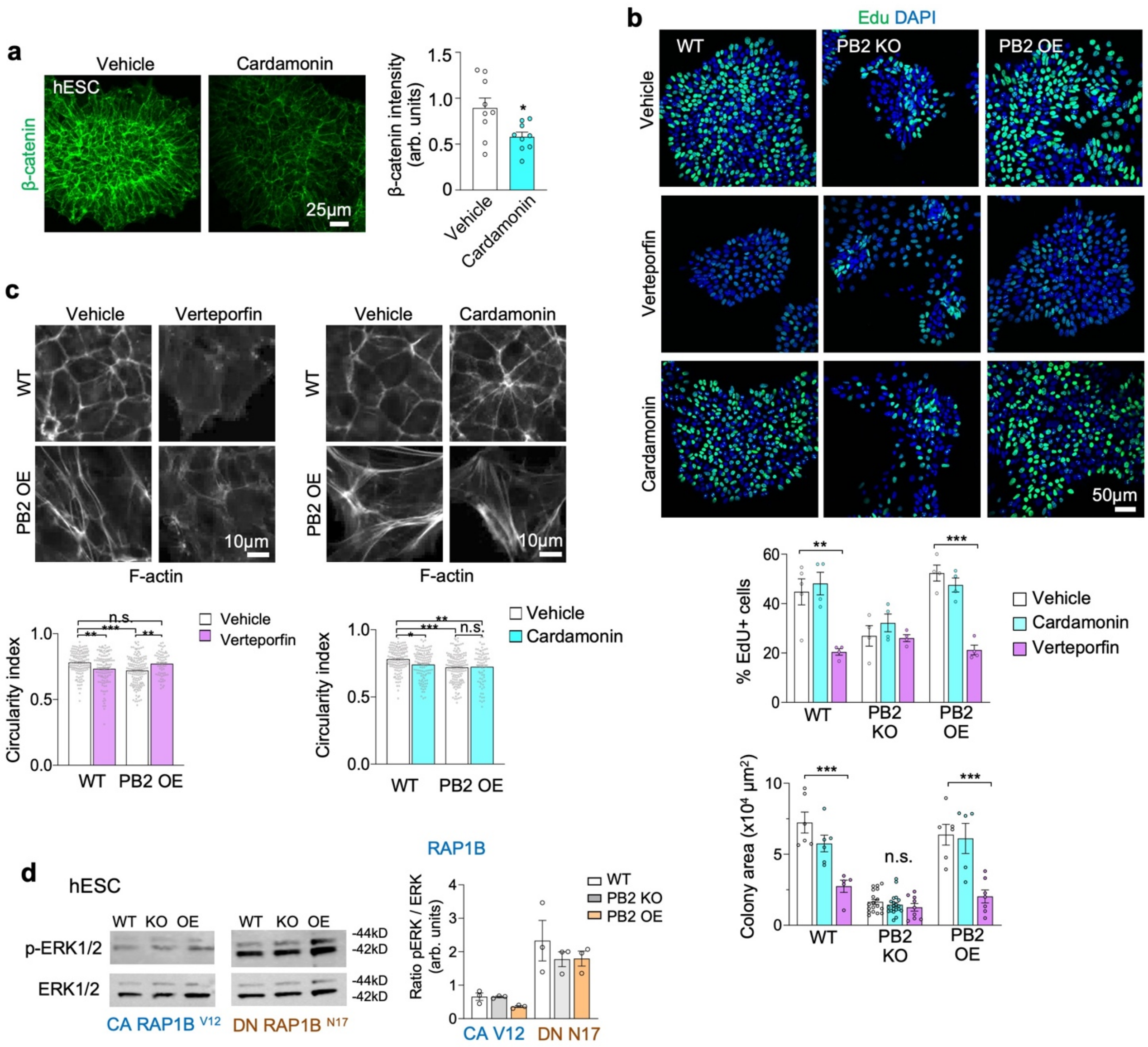

Supplementary Figure 10. Inhibition of YAP1 and β-catenin signaling and its impact on hESC growth.

a, Treatment of hESC colonies with β-catenin inhibitor cardamonin resulted in reduction of β-catenin levels. n=9 colonies per group. Two-sided unpaired *t*-test. *P=0.019.arb.units, arbitrary units. Data represent mean ± SEM.

b, Treatment of hESC colonies with YAP inhibitor verteporfin resulted in significant decrease in proliferation of WT and PB2 OE ESCs, as revealed by EdU labeling (30 min pulse) and by measurement of colony areas. Colonies of PB2 KO hESCs were not affected. Cardamonin had no effect on colony growth. For EdU: vehicle, n=1641, 1120, 1455 cells for WT, KO, OE, respectively; verteporfin, n=990, 1106, 1384; cardamonin, n=1129, 1298, 1496. **P=0.0048 and ***P<0.0001. For colony size: vehicle, n=6, 18, 6 colonies for WT, KO, OE, respectively; verteporfin, WT, n=5, 9, 7 colonies; cardamonin, n=6, 20, 5 colonies. ***P=0.0004 (WT veh vs. WT vert), ***P=0.001 (PB2 OE veh vs. PB2 OE vert). n.s., not significant. One-way ANOVA followed by Dunnett's multiple comparisons to compare treatments within the groups. Data represent mean ± SEM.

c, Images of hESCs show the impact of verteporfin or cardamonin on cortical F-actin, cell shape and cellular organization in colonies. Quantification of cell circularity index: One-way ANOVA with Tukey's post hoc test. For vehicle, WT, n=134 cells; PB2 OE, n=147 cells. For verteporfin, WT, n=114 cells; PB2 OE, n=67 cells. For cardamonin, WT, n=116 cells; PB2 OE, n=70 cells. For verteporfin treatment: **P=0.004 (PB2 OE veh vs. PB2 OE Vert), **P=0.0018 (WT veh vs. WT Vert), and ***P<0.0001 (WT veh vs. PB2 OE). For cardamonin treatment: *P=0.016 (WT veh vs. WT card), **P=0.0014 (WT veh vs. PB2 OE card), and ***P<0.0001 (WT veh vs. PB2 OE veh). n.s., not significant. Data represent mean ± SEM.

d, WBs and quantification show that expression of RAP1B-N17 (dominant-negative (DN) isoform) in hESCs resulted in increased levels of phosphorylated ERK1/2 in all three *PLXNB2* genotypes relative to cells expressing RAP1B-V12 (constitutively-active (CA) isoform). ERK1/2 served as loading control. n=3 samples per group. arb. units, arbitrary units. Data represent mean ± SEM.

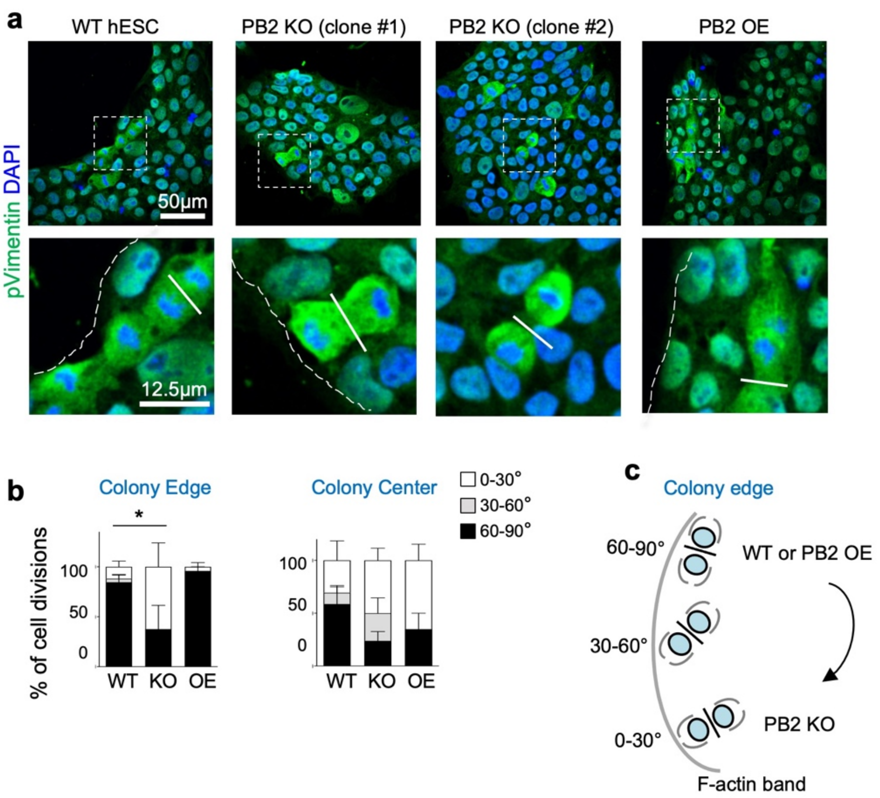

Supplementary Figure 11. Plexin-B2 affects mitotic spindle orientation in hESC colonies.

a, Confocal images of indicated hESC colonies, stained for phospho-Vimentin to highlight dividing cells. Division planes are indicated by lines in enlarged images. Dashed lines mark colony edges. Two KO clones were shown (clone #1 and clone #2).

b, Quantification of division planes that were in parallel, oblique, or perpendicular orientation to the tangent of the colony edge. Two-way ANOVA followed by Dunnett's post hoc test. For colony edge, n=21, 10, 24 cell divisions for WT, KO, and OE, respectively. For colony center, n=17, 14, 15 for WT, KO, and OE, respectively. **P*=0.039. Data represent mean ± SEM.

c, Diagram depicting reduced probability of division planes perpendicular to the tangent of colony border in Plexin-B2 KO colonies.

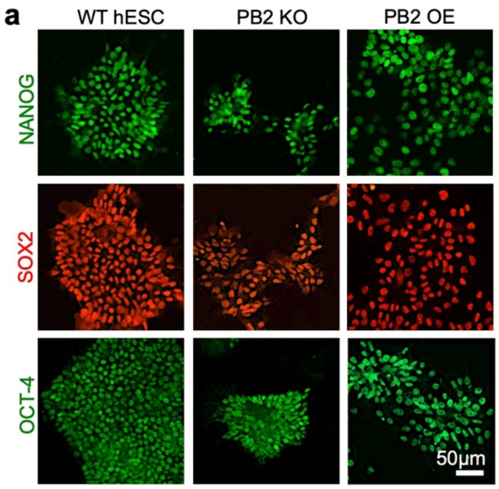

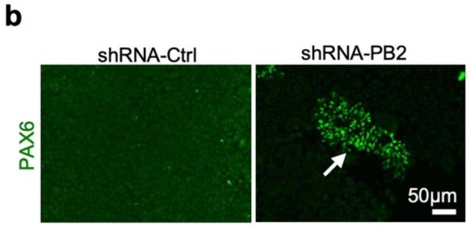

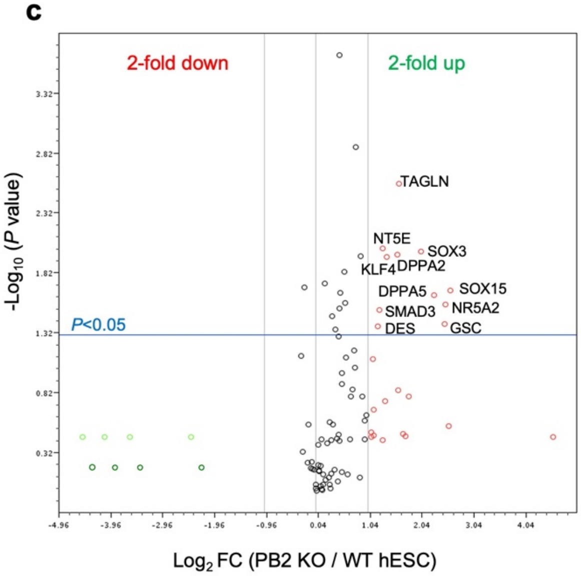

Embryonic Stem Cell (ESC) Specific Genes:
Markers: ALPL, GDF3 (VGR2), TDGF1, TERT (Housekeeping)
Chromatin Regulators: CHD1, CHD7, KAT5.
Cofactors: CNOT3, NR0B1, PAF1, TRIM28.
Transcription Factors: ESRRB, KLF4, LIN28A, MYC, NANOG, POU5F1 (OCT4), PRDM14, SALL4, SMAD1, SMAD2, SMAD3, SOX2, STAT3, TBX3, TCF3, THAP11, UTF1, ZFX.

Pluripotency: AICDA, CCNA2, CDC42, CDK1 (CDC2), DPPA2, DPPA3, DPPA4, DPPA5, FGF2 (bFGF), HSPA9, MYBL2, NR5A2, NR6A1, OTX2, RIF1, SCN1A, SOX15, TCL1A, ZFP42.

PSC Genes/Enhancers: AICDA, KLF4, LIN28A, MYC, NANOG, POU5F1 (OCT4), SOX2, UTF1.

Germ Layers:
Ectoderm: EN2, FGF5, MEIS1, NCAM1, PAX6.
Mesoderm: DES, HAND1, MESP1, MIXL1, MYOD1, T (Brachyury).
Endoderm: GATA1, GATA6, GSC, HNF4A, SOX17, SOX7.

Early Cell Types:
Neural Progenitors: GFAP, NES, OLIG2, SOX3.
Cardiac Progenitors: ISL1, NKX2-5.
Early Smooth Muscle Cells: ACTA2, TAGLN.
Hematopoietic Stem Cells/Early Endothelial Cells: ETV2, FLII, GATA2, TAL1, TEK (TIE2).
Mesenchymal Stem Cells: CD44, ENG (EVI1), NT5E, THY1.
Hepatic Tissue: ALB, TAT.

Supplementary Figure 12. Plexin-B2 influences hESC differentiation.

a, IF images reveal comparable expression of the stem cell markers NANOG, SOX2, and OCT4 in hESCs of different genotypes.

b, IF images show patch of cells in Plexin-B2 KD hESC colony with induction of neuroprogenitor marker PAX6 (arrow).

c, Left**,** volcano plot of qRT-PCR expression data shows differentially expressed genes (DEGs, >=2 fold change, *P*<0.05). Right, list of marker genes tested in the qRT-PCR array, with DEGs labeled in green denoting upregulation. The *P* values were calculated using a two-sided unpaired *t*-test on the replicate $2^{-\Delta CT}$ values of PB2 KO vs. WT.

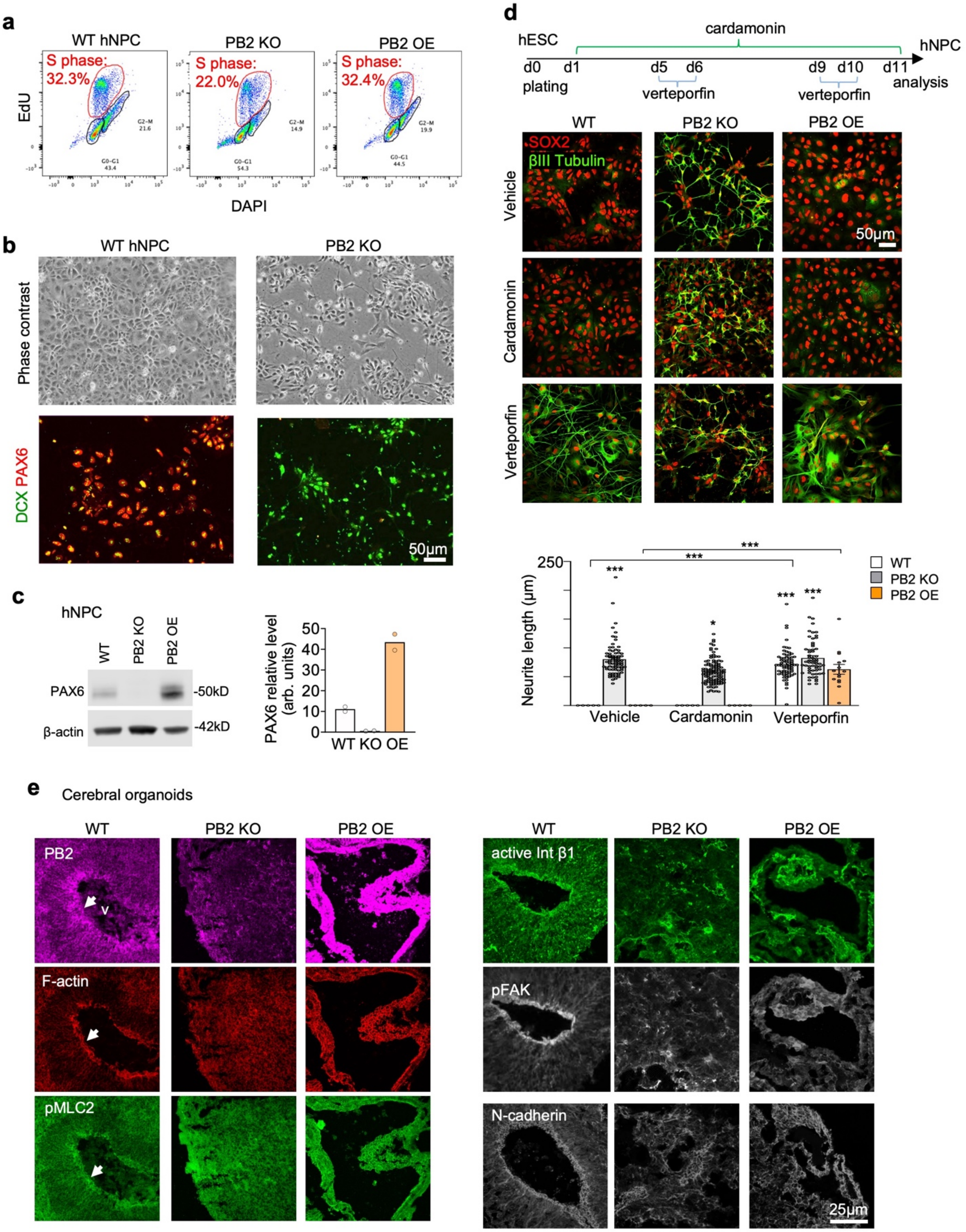

Supplementary Figure 13. Plexin-B2 plays a role in neurodifferentiation and maintenance of cytoarchitecture of neuroepithelium.

a, Flow cytometry data show reduced fraction of cells in S phase for *PLXNB2* KO hNPCs compared to WT or *PLXNB2* OE. The gating strategy used for the flow cytometry was the same as in Fig. S1f.

b, Phase-contrast and IF images show induction of neuroblast marker DCX but reduced PAX6 expression in *PLXNB2* KO cells as compared to WT at day 8 of differentiation.

c, WB and quantification show loss of PAX6 expression in Plexin-B2 KO hNPCs, and induction in Plexin-B2 OE hNPCs. n=2 samples per group. arb. units, arbitrary units. Data represent the mean.

d, The Plexin-B2 KO led to spontaneous neuronal differentiation during hNPC inductions, as measured by length of β-III

tubulin+ neurites. While cardamonin did not alter differentiation, verteporfin promoted neuronal differentiation in all genotype conditions. Note that verteporfin was applied in two 24 hr pulses to reduce toxicity effects. Kruskal-Wallis test followed by Dunn's multiple comparisons test to compare to WT group. Two-sided Mann–Whitney U test was applied when comparing data from two groups (brackets). n=5 fields per group. *P=0.042 (WT veh vs. PB2 KO card), ***P=0.0006 (WT veh vs. WT vert), ***P<0.0001 for the rest. Data represent mean ± SEM.

e, Confocal images of cross sections of cerebral organoids (day 42) demonstrate disorganized cytoarchitecture in *PLXNB2* KO and OE organoids, as shown by staining for markers related to actomyosin network and cell adhesion.

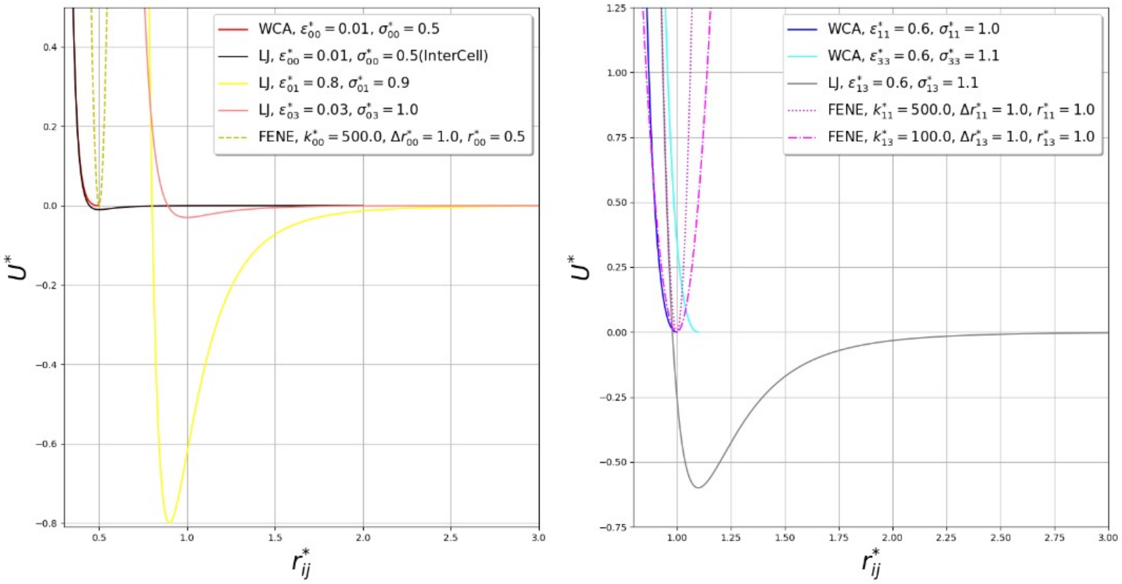

Supplementary Figure 14. Potential energy used in the molecular dynamics model.

The plots show the Weeks-Chandler-Anderson (WCA), Lennard-Jones (L-J) and Finite Extensible Nonlinear Elastic (FENE) potential for selected values of potential parameters.
