# Supplementary material for: Plexin-B2 orchestrates collective stem cell dynamics via actomyosin contractility, cytoskeletal tension and adhesion
Source: Nat Commun. 2021 Oct 14;12:6019. doi: 10.1038/s41467-021-26296-7 (PMC8517024; doi:10.1038/s41467-021-26296-7)
Supplement: Supplementary file 2 — Description of Additional Supplementary Files [file 41467_2021_26296_MOESM2_ESM.docx]

**Description of Additional Supplementary Files**

**Title: Supplementary Data 1**

Description: List of gene-specific primers used for qRT-PCR and list of oligonucleotides used for cloning of short hairpin (shRNA) vectors for gene knockdown.

**Title: Supplementary Movie 1**

**Live-cell imaging of F-actin dynamics in hESC colonies.**

Description: WT, *PLXNB2* KO, and *PLXNB2* OE hESCs were labeled with SPY555-Actin and NucSpot after 18 hours of culture and imaged for 21 hours, with frames taken every 20 min. Note the differences in cortical F-actin distribution and cell morphology in mutant vs. WT colonies.

**Title: Supplementary Movie 2**

**Mathematical simulation of mechanical rigidity of cells with constant membrane-membrane attraction energy and different attraction energy of actin filament head and cell membrane.**

Description: The videos show evolution of arrangement of four cells. When the system reaches equilibrium, the virtual AFM tip (green dot on the right) begins to indent cell membranes. The membrane-membrane attraction energy (*ɛ*00) was fixed at 0.01 and the attraction energy of actin filament head and cell membrane (*ɛ*03) was variable (0.03, 4.00 and 30.0). Note that the total distance traveled by the AFM tip was around 60 µm, far beyond the tip displacement in the graph in Fig. 5c, which ranged from 0 to 3 µm, similar to the AFM elastography experiment. The beads represent actin filaments (red), actin filament head (gray), cell membrane (yellow), and AFM tip (green).

**Title: Supplementary Movie 3**

**Mathematical simulation of mechanical rigidity of cells with constant attraction energy of actin filament head and cell membrane and different membrane-membrane attraction energy.**

Description: The videos show evolution of cellular arrangement of four cells. When the system reaches equilibrium, the virtual AFM tip (green dot on the right) begins to indent cell membranes. The attraction energy of actin filament head and cell membrane (*ɛ*03) was fixed at 0.03 and the membrane-membrane attraction energy (*ɛ*00) was variable (0.01, 1.0, and 10.0). Note that the total distance traveled by the AFM tip was around 60 µm, far beyond the tip displacement shown in the graph in Fig. 5c, which ranged from 0 to 3 µm, similar to the AFM elastography experiment. The beads represent actin filaments (red), actin filament head (gray), cell membrane (yellow), and AFM tip (green).
