# Supplementary material for: Plexin-B2 orchestrates collective stem cell dynamics via actomyosin contractility, cytoskeletal tension and adhesion
Source: Nat Commun. 2021 Oct 14;12:6019. doi: 10.1038/s41467-021-26296-7 (PMC8517024; doi:10.1038/s41467-021-26296-7)
Supplement: Supplementary file 7 — Reporting Summary [file 41467_2021_26296_MOESM7_ESM.pdf]

nature portfolio

Corresponding author(s): Hongyan Zou, Roland Friedel

Last updated by author(s): Sep 13, 2021

Reporting Summary

Nature Portfolio wishes to improve the reproducibility of the work that we publish. This form provides structure for consistency and transparency in reporting. For further information on Nature Portfolio policies, see our Editorial Policies and the Editorial Policy Checklist.

Statistics

For all statistical analyses, confirm that the following items are present in the figure legend, table legend, main text, or Methods section.

n/a	Confirmed	
☐	☒	The exact sample size (*n*) for each experimental group/condition, given as a discrete number and unit of measurement
☐	☒	A statement on whether measurements were taken from distinct samples or whether the same sample was measured repeatedly
☐	☒	The statistical test(s) used AND whether they are one- or two-sided<br>*Only common tests should be described solely by name; describe more complex techniques in the Methods section.*
☒	☐	A description of all covariates tested
☐	☒	A description of any assumptions or corrections, such as tests of normality and adjustment for multiple comparisons
☐	☒	A full description of the statistical parameters including central tendency (e.g. means) or other basic estimates (e.g. regression coefficient) AND variation (e.g. standard deviation) or associated estimates of uncertainty (e.g. confidence intervals)
☐	☒	For null hypothesis testing, the test statistic (e.g. *F*, *t*, *r*) with confidence intervals, effect sizes, degrees of freedom and *P* value noted<br>*Give P values as exact values whenever suitable.*
☒	☐	For Bayesian analysis, information on the choice of priors and Markov chain Monte Carlo settings
☒	☐	For hierarchical and complex designs, identification of the appropriate level for tests and full reporting of outcomes
☒	☐	Estimates of effect sizes (e.g. Cohen's *d*, Pearson's *r*), indicating how they were calculated

Our web collection on statistics for biologists contains articles on many of the points above.

Software and code

Policy information about availability of computer code

Data collection	Olympus cellSens Entry (phase contrast images); Zeiss AxioVision Rel. 4.8 (fluorescence microscopy images); Zeiss Zen software (confocal microscopy images); BD FACSDiva 8.0.2 Software (FACS and flow cytometry data collection); Spot 4.6 (macroscopic images); Leica Las X software (FRET images); Wavemetrics Igor Pro 6 (AFM images); Illumina platform HiSeq2500 (RNASeq).
Data analysis	GraphPad Prism 8.0 (plotting, statistical analysis); SnapGene (sequencing and cloning analysis); LI-COR Image Studio Lite (WB analysis); ImageJ, Matlab and PyCharm (FRET heatmaps); BD FACSDiva 8.0.2 Software and FlowJo (flow cytometry data ana;lysis); Qiagen GeneGlobe Data Analysis Center (qPCR array data analysis); Adobe Illustrator CS5 (illustration preparation); Microsoft Excel (data handling); Microsoft PowerPoint (figure preparation); Applied Biosystems 7900HT Fast Real-Time PCR System Software (qPCR data analysis); ImageJ 1.53k (morphological measurements and FRET analysis); Wavemetrics Igor Pro 6 (AFM data analysis); Imaris (videography); DESeq2 package, Enrichr, NetworkAnalyst 3.0 and Ingenuity Pathway Analysis (RNASeq analysis).

For manuscripts utilizing custom algorithms or software that are central to the research but not yet described in published literature, software must be made available to editors and reviewers. We strongly encourage code deposition in a community repository (e.g. GitHub). See the Nature Portfolio guidelines for submitting code & software for further information.

Data

Policy information about availability of data

All manuscripts must include a data availability statement. This statement should provide the following information, where applicable:

- Accession codes, unique identifiers, or web links for publicly available datasets
- A description of any restrictions on data availability
- For clinical datasets or third party data, please ensure that the statement adheres to our policy

The RNA-seq data generated in this study have been deposited in the NCBI Gene Expression Omnibus (GEO) database under accession code GSE158017 (https://www.ncbi.nlm.nih.gov/geo/query/acc.cgi?acc=GSE158017). Source data underlying graphs and full Western blot images are provided in Source Data. All other data that support the findings of this study are available from the corresponding author R.H.F. upon reasonable request.

The hg38 genome was obtained from Ensembl Release 90 (https://useast.ensembl.org/index.html).

Molecular dynamics (MD) codes have been deposited at Github (github.com/diasrodri/SimCellMD-1) and Zenodo (zenodo.org/record/4977917#.YMyfWTZKjyU).

Field-specific reporting

Please select the one below that is the best fit for your research. If you are not sure, read the appropriate sections before making your selection.

☒ Life sciences ☐ Behavioural & social sciences ☐ Ecological, evolutionary & environmental sciences

For a reference copy of the document with all sections, see nature.com/documents/nr-reporting-summary-flat.pdf

Life sciences study design

All studies must disclose on these points even when the disclosure is negative.

Sample size	No statistical methods were used to predetermine the sample size. The samples sizes were determined to be adequate based on the magnitude and consistency of differences between groups. This method has been used by previous studies with similar experiments (Dang et al., Nat. Commun 2021; Weigelin et al., Nat Commun. 2021, Liu et al., Nat. Commun 2021). Sample sizes for each experiment are stated in figure legends. Three clone lines for PLXNB2 KO hESCs and population, as well as stably transfected WT, PLXNB2 shRNA-KD and PLXNB2 OE and dox-inducible OE (PLXNB2 iOE) hESCs lines were used throughout the study, typically 3-4 replicates per experiment. For cerebral organoids, a minimum of two batches and at least 3-4 organoids per batch were used for each condition.
Data exclusions	No data was excluded.
Replication	The experiments were repeated successfully at least three times independently with similar outcomes.
Randomization	Sample groups tested in vitro were randomly assigned. All sample collection was performed in random order for different experimental replicates. For microscopy imaging experiments, multiple fields were randomly acquired for all experimental groups and controls. For comparative analysis that required the use of control lines for standard acquisition parameters (e.g., Fluorescence intensity from microscopy imaging assays), control conditions were assayed first, and then the other sample acquisitions were performed randomly.
Blinding	The investigators were not blinded during experiments and data collection to group allocation. For high-content image analysis, images were previously coded and analyzed by a second researcher who was blinded for experimental groups. RNA-seq data collection was performed by core facility members who were blinded to the experimental groups.

Reporting for specific materials, systems and methods

We require information from authors about some types of materials, experimental systems and methods used in many studies. Here, indicate whether each material, system or method listed is relevant to your study. If you are not sure if a list item applies to your research, read the appropriate section before selecting a response.

Materials & experimental systems

n/a	Involved in the study	
☐	☒	Antibodies
☐	☒	Eukaryotic cell lines
☒	☐	Palaeontology and archaeology
☒	☐	Animals and other organisms
☒	☐	Human research participants
☒	☐	Clinical data
☒	☐	Dual use research of concern

Methods

n/a	Involved in the study	
☒	☐	ChIP-seq
☐	☒	Flow cytometry
☒	☐	MRI-based neuroimaging

Antibodies

Antibodies used

The following primary antibodies were used for ICC/IF:

anti-sheep plexin-b2 (ecd) (1:300, R&D systems, AF5329),
anti-rabbit plexin-b2 (icd) (1:200, Abcam, ab193355),
anti-rabbit sox2 (1:200, Abcam, ab97959),
anti-guinea pig dcx (1:500, EMD Millipore, ab2253),
anti-rabbit pmlc2 (1:200, Cell Signaling, 3671),
anti-rat e-cadherin (1:200, Life Technologies, 131900),
anti-mouse n-cadherin (1:200, BD Bioscience, 610920),
anti-rabbit zo-1 (1:200, Thermo, 61-7300),
anti-mouse integrin β1 (active, huts-4) (1:200, EMD Millipore, MAB2079Z),
anti-mouse, oct-4 (1:500, Abcam, ab184665),
anti-rabbit pFak (1:200, Thermo, 44-624G),
anti-rabbit nanog (1:200, Abcam, ab109250),
anti-rabbit acaspase3 (1:500, R&D systems, AF835),
anti-mouse yap (1:200, Santa Cruz, sc-101199),
anti-mouse β-catenin (1:200, BD Bioscience, 610153),
anti-mouse pax6 (1:200, Abcam, ab78545),
anti-βIII tubulin (tuj1) (1:100, R&D systems, MAB1195),
anti-mouse paxillin (1:200, Invitrogen, AHO0492),
anti-mouse p-vimentin (phospho S55) (1:200, Abcam, ab22651),
anti-rabbit fibronectin (1:200, EMD Millipore, ab2033).

The following secondary antibodies were used for ICC/IF:

Alexa Fluor 488, 594, or 647-conjugated donkey anti-goat, -rabbit, -rat, or -mouse IgG and anti-guinea pig IgG (1:300, Jackson ImmunoResearch Laboratories).

The following primary antibodies were used for Western blot:

anti-rabbit β-actin (1:10,000, Sigma, A1978),
anti-sheep plexin-b2 (ecd) (1:500, R&D Systems, AF5329),
anti-rabbit β1-integrin (4706s) (1:1,000, Cell Signaling, 4706),
anti-mouse yap (1:500, Santa Cruz, sc-101199),
anti-rabbit phospho-yap (Ser127) (1:1,000, Cell Signaling, 4911S),
anti-rabbit phospho-p44/42 mapk (erk1/2) (thr202/tyr204) (1:1,000, Cell Signaling, 9101S),
anti-mouse p44/42 mapk (erk1/2) (3a7) (1:1,000, Cell Signaling, 9107S),
anti-mouse β-catenin (1:1,000, BD Bioscience, 610153),
anti-rabbit sox2 (1:10,000, Abcam, ab97959),
anti-rabbit pax6 (1:2,000, BioLegend, 901302),
anti-rabbit gapdh (1:1,000, Cell Signaling, 2118),
anti-rabbit h3k4me2 (0.5 μg/ml, Active Motif, 39914).

The following secondary antibodies were used for Western Blot:

IRDye 800CW donkey anti-mouse (1:10,000, Li-Cor Biosciences, 926-32212),
680RD-donkey anti-goat (1:10,000, Li-Cor Biosciences, 926-68024),
680RD-donkey anti-rabbit (1:10,000, Li-Cor Biosciences, 926-68073).

The following primary antibodies were used for function-blocking experiment:

anti-mouse INTEGRIN β1 (P5D2) (used at 10 μg/mL, Santa Cruz, sc-13590)
ChromPure Mouse IgG, whole molecule (1:1,000, Jackson ImmunoResearch Laboratories, 015-000-003).

Validation

Anti-Plexin B2 (ECD) antibody used for ICC/IF was validated in HeLa human cervical epithelial carcinoma parental cell line and it was not detectable in Plexin B2 knockout HeLa cell line and cited by 8 publications.
https://www.rndsystems.com/products/human-plexin-b2-antibody_af5329

Anti-Plexin B2 (ICD) antibody used for ICC/IF was validated by knockout and cited by 2 publications.
https://www.abcam.com/plexin-b2mm1-antibody-epr9965-ab193355.html

Anti-SOX2 antibody used for ICC/IF was cited by 497 publications.
https://www.abcam.com/sox2-antibody-ab97959.html?productWallTab=ShowAll

Anti-DCX antibody used for ICC/IF was cited by 138 publications.
https://www.emdmillipore.com/US/en/product/Anti-Doublecortin-Antibody,MM_NF-AB2253

Anti-pMLC2 antibody used for ICC/IF was cited by 400 publications.
https://www.cellsignal.com/products/primary-antibodies/phospho-myosin-light-chain-2-ser19-antibody/3671

Anti-E-Cadherin antibody used for ICC/IF was cited by 193 publications.

https://www.thermofisher.com/antibody/product/E-cadherin-Antibody-clone-ECCD-2-Monoclonal/13-1900

Anti-N-Cadherin antibody used for ICC/IF was cited by 436 publications.
https://www.bdbiosciences.com/en-us/products/reagents/microscopy-imaging-reagents/immunofluorescence-reagents/purified-mouse-anti-n-cadherin.610920

Anti-ZO-1 antibody used for ICC/IF was cited by 1,011 publications.
https://www.thermofisher.com/antibody/product/ZO-1-Antibody-Polyclonal/61-7300

Anti-INTEGRIN β1 (active, HUTS-4) antibody used for ICC/IF was cited by 86 publications.
https://www.emdmillipore.com/US/en/product/Anti-Integrin-1-Antibody-activated-clone-HUTS-4-Azide-Free,MM_NF-MAB2079Z

Anti-OCT-4 antibody used for ICC/IF was cited by 13 publications.
https://www.abcam.com/oct4-antibody-gt486-ab184665.html?productWallTab=ShowAll

Anti-pFAK antibody used for ICC/IF was cited by 147 publications.
https://www.thermofisher.com/antibody/product/Phospho-FAK-Tyr397-Antibody-Polyclonal/44-624G

Anti-NANOG antibody used for ICC/IF was cited by 98 publications.
https://www.abcam.com/nanog-antibody-epr20272-ab109250.html?productWallTab=ShowAll

Anti-aCASPASE3 antibody used for ICC/IF was cited by 157 publications.
https://www.rndsystems.com/products/human-mouse-active-caspase-3-antibody_af835

Anti-YAP antibody used for ICC/IF was cited by 484 publications.
https://www.scbt.com/p/yap-antibody-63-7

Anti-β-CATENIN antibody used for ICC/IF was was cited by 6 publications.
https://www.bdbiosciences.com/en-us/products/reagents/microscopy-imaging-reagents/immunofluorescence-reagents/purified-mouse-anti-catenin.610154

Anti-PAX6 antibody used for ICC/IF was cited by 9 publications.
https://www.abcam.com/pax6-antibody-ab62803.html

Anti-TUJ1 antibody used for ICC/IF was cited by 162 publications.
https://www.rndsystems.com/products/neuron-specific-beta-iii-tubulin-antibody-tuj-1_mab1195#product-datasheets

Anti-Paxillin antibody used for ICC/IF was cited by 15 publications.
https://www.thermofisher.com/antibody/product/Paxillin-Antibody-clone-5H11-Monoclonal/AHO0492

Anti-pVimentin (phospho S55) antibody used for ICC/IF was cited by 43 publications.
https://www.abcam.com/vimentin-phospho-s55-antibody-4a4-ab22651.html

Anti-Fibronectin antibody used for ICC/IF was cited by 62 publications.
https://www.emdmillipore.com/US/en/product/Anti-Fibronectin-Antibody,MM_NF-AB2033

Anti-β-actin antibody used for WB was cited by 3,226 publications.
https://www.sigmaaldrich.com/US/en/product/sigma/a1978

Anti-Plexin B2 (ECD) antibody used for WB was validated in HeLa human cervical epithelial carcinoma parental cell line and it was not detectable in Plexin B2 knockout HeLa cell line and cited by 8 publications.
https://www.rndsystems.com/products/human-plexin-b2-antibody_af5329

Anti-INTEGRIN β1 (4706S) antibody used for WB was cited by 80 publications.
https://www.cellsignal.com/products/primary-antibodies/integrin-b1-antibody/4706

Anti-YAP antibody used for WB was cited by 484 publications.
https://www.scbt.com/p/yap-antibody-63-7

Anti-pYAP antibody used for WB was cited by 361 publications.
https://www.cellsignal.com/products/primary-antibodies/phospho-yap-ser127-antibody/4911

Anti-phospho-p44/42 MAPK (Erk1/2) (Thr202/Tyr204) used for WB was cited by 5715 publications.
https://www.cellsignal.com/products/primary-antibodies/phospho-p44-42-mapk-erk1-2-thr202-tyr204-antibody/9101

Anti-p44/42 MAPK (Erk1/2) (3A7) used for WB was cited by 446 publications.
https://www.cellsignal.com/products/primary-antibodies/p44-42-mapk-erk1-2-3a7-mouse-mab/9107

Anti-β-CATENIN antibody used for WB was cited by 6 publications.
https://www.bdbiosciences.com/en-us/products/reagents/microscopy-imaging-reagents/immunofluorescence-reagents/purified-mouse-anti-catenin.610154

Anti-SOX2 antibody used for WB was cited by 497 publications.
https://www.abcam.com/sox2-antibody-ab97959.html?productWallTab=ShowAll

Anti-PAX6 antibody used for WB was cited by 223 publications.

https://www.biolegend.com/en-us/products/purified-anti-pax-6-antibody-11511?GroupID=GROUP26

Anti-GAPDH antibody used for WB was cited by 4,276 publications.
https://www.cellsignal.com/products/primary-antibodies/gapdh-14c10-rabbit-mab/2118

Anti-H3K4me2 antibody used for WB was cited by 2 publications.
https://www.activemotif.com/catalog/details/39913/histone-h3-dimethyl-lys4-antibody-pab-1

ChromPure Isotype IgG, whole molecule
https://www.jacksonimmuno.com/catalog/products/015-000-003

Anti-INTEGRIN β1 (P5D2) antibody was used for function-blocking experiment was cited by 2 publications.
https://www.scbt.com/p/integrin-beta1-antibody-p5d2

Eukaryotic cell lines

Policy information about cell lines

Cell line source(s)	H9 hESC line (WA09, WiCell; NIH registration number: 0062).<br>HEK293T cells (Takara, no. 632180) was used for the production of lentiviral particles by co-transfection with lentiviral plasmids.
Authentication	The H9 cell line has been authenticated by the Pluripotent Stem Cell Core Facility at Icahn School of Medicine at Mount Sinai and also authenticated in-house by immunostaining for pluripotency markers, as well as karyotypes.
Mycoplasma contamination	The cell line used in this work has been tested negative for mycoplasma contamination.
Commonly misidentified lines<br>(See ICLAC register)	No commonly misdentified cell lines lines were used in this work.

Flow Cytometry

Plots

Confirm that:

☒ The axis labels state the marker and fluorochrome used (e.g. CD4-FITC).

☒ The axis scales are clearly visible. Include numbers along axes only for bottom left plot of group (a 'group' is an analysis of identical markers).

☒ All plots are contour plots with outliers or pseudocolor plots.

☒ A numerical value for number of cells or percentage (with statistics) is provided.

Methodology

Sample preparation	For FACS of hESCs carrying vinculin tension bionsensor: hESCs were dissociated with Accutase, pelleted and resuspended in FACS buffer (Hibernate-E low fluorescence (BrainBits) with 0.2% BSA and 20 µg/ml DNase I (Worthington)). Cells were passed through 35 µm mesh filter into round bottom tubes (Falcon). DAPI (Invitrogen) was added to cell suspensions at 5 µg/ml to stain dead cells. Cell suspensions were sorted by FACS to enrich for populations of live cells with highest CFP-YFP expression.<br><br>For Click-iT EdU Proliferation Assay: to assess the proliferative potential of hESCs and hNPCs, 3x10^5 cells were plated in 6 well plate, and after 3 days of culture, pulse-labeled with 10µM EdU (5-ethynyl-2-deoxyuridine, ThermoFisher), 20 min for hESCs and 90 min for hNPCs, and harvested using Accutase (BD Biosciences). Detection of EdU incorporation was performed with the Click-iT EdU Alexa Fluor 488 Cell Proliferation Assay Kit (ThermoFisher), according to the manufacturer's instructions. In brief, cells were washed twice in PBS/1% BSA, fixed in 100µl Click-iT fixative, and stored at 4°C overnight. In the next day, cells were washed twice in saponin-based permeabilization and wash reagent. The Click-iT EdU reaction cocktail was prepared according to the manufacturer's instructions and added to the cell pellet. Samples were incubated for 30 min at room temperature in the dark, and washed with saponin-based permeabilization and wash reagent. DAPI was added to cell suspensions at 5 µg/ml and incubated at room temperature in the dark. Cells were passed through 35 µm mesh filter into round bottom tubes as described above for FACS and analyzed by flow cytometry.
Instrument	We used BD FACSAria IIu for FACS and BD LSRIIA for flow cytometry experiments.
Software	The data was evaluated with FACSDiva 8.0.2 and FlowJo software.
Cell population abundance	For FACS of hESC carrying vinculin tension biosensor we recovered every cell that showed positive for CFP-YFP above the maximum fluorescence intensity detected for negative control cells.<br>For Click-iT EdU Proliferation Assay we registered 10.000 positive events for each group.

Gating strategy

For Click-iT EdU Proliferation Assay we established the gating based on the negative control using FlowJo software to define the % of cells in S, G0-G1 and G2-M phase. This approach was based on similar studies and following the same criteria for all groups.

☒ Tick this box to confirm that a figure exemplifying the gating strategy is provided in the Supplementary Information.
